# Comparison of Collaborative Goal Setting With Enhanced Education for Managing Diabetes-Associated Distress and Hemoglobin A_1c_ Levels: A Randomized Clinical Trial

**DOI:** 10.1001/jamanetworkopen.2022.9975

**Published:** 2022-05-04

**Authors:** LeChauncy Woodard, Amber B. Amspoker, Natalie E. Hundt, Howard S. Gordon, Brian Hertz, Edward Odom, Anne Utech, Javad Razjouyan, Suja S. Rajan, Nipa Kamdar, Jasmin Lindo, Lea Kiefer, Praveen Mehta, Aanand D. Naik

**Affiliations:** 1 Houston Center for Innovations in Quality, Effectiveness, and Safety, Michael E. Debakey Veterans Affairs (VA) Medical Center, Houston, Texas; 2 Department of Medicine, Baylor College of Medicine, Houston, Texas; 3 Humana Integrated Health System Sciences Institute, University of Houston, Houston, Texas; 4 Department of Health Systems and Population Health Sciences, University of Houston College of Medicine, Houston, Texas; 5 Department of Psychiatry and Behavioral Sciences, Baylor College of Medicine, Houston, Texas; 6 Jesse Brown Veterans Affairs Medical Center, VA Center of Innovation for Complex Chronic Healthcare, Chicago, Illinois; 7 Section of Academic Internal Medicine, Department of Medicine, University of Illinois Chicago; 8 Institute for Health Research and Policy, University of Illinois Chicago; 9 Department of Medicine, Loyola University Chicago, Stritch School of Medicine, Chicago, Illinois; 10 Division of Internal Medicine, Hines Veterans Affairs Hospital, Hines, Illinois; 11 Office of Veterans Access to Care, US Department of Veterans Affairs, Washington, DC; 12 Nutrition and Food Services, US Department of Veterans Affairs, Washington, DC; 13 Big Data Scientist Training Enhancement Program (BD-STEP), VA Office of Research and Development, Washington, DC; 14 Department of Management, Policy, and Community Health, School of Public Health, University of Texas Health Science Center, Houston; 15 Veterans Integrated Network 16 (Great Lakes VA Health System), Chicago, Illinois; 16 Consortium on Aging, University of Texas Health Science Center, Houston

## Abstract

**Importance:**

Type 2 diabetes is a prevalent and morbid condition. Poor engagement with self-management can contribute to diabetes-associated distress and hinder diabetes control.

**Objective:**

To evaluate the implementation and effectiveness of Empowering Patients in Chronic Care (EPICC), an evidence-based intervention to improve diabetes-associated distress and hemoglobin A_1c_ (HbA_1c_) levels after the intervention and after 6-month maintenance.

**Design, Setting, and Participants:**

This hybrid (implementation-effectiveness) randomized clinical trial was performed in Veterans Affairs clinics across Illinois, Indiana, and Texas from July 1, 2015, to June 30, 2017. Participants included adults with uncontrolled type 2 diabetes (HbA_1c_ level >8.0%) who received primary care during the prior year in participating clinics. Data collection was completed on November 30, 2018, and data analysis was completed on June 30, 2020. All analyses were based on intention to treat.

**Interventions:**

Participants in EPICC attended 6 group sessions based on a collaborative goal-setting theory led by health care professionals. Clinicians conducted individual motivational interviewing sessions after each group. Usual care was enhanced (EUC) with diabetes education.

**Main Outcomes and Measures:**

The primary outcome consisted of changes in HbA_1c_ levels after the intervention and during maintenance. Secondary outcomes included the Diabetes Distress Scale (DDS), Morisky Medication Adherence Scale, and Lorig Self-efficacy Scale. Secondary implementation outcomes included reach, adoption, and implementation (number of sessions attended per patient).

**Results:**

A total of 280 participants with type 2 diabetes (mean [SD] age, 67.2 [8.4] years; 264 men [94.3]; 134 non-Hispanic White individuals [47.9%]) were equally randomized to EPICC or EUC. Participants receiving EPICC had significant postintervention improvements in HbA_1c_ levels (*F*_1, 252_ = 9.12, Cohen *d* = 0.36 [95% CI, 0.12-0.59]; *P* = .003) and DDS (*F*_1, 245_ = 9.06, Cohen *d* = 0.37 [95% CI, 0.13-0.60]; *P* = .003) compared with EUC. During maintenance, differences between the EUC and EPICC groups remained significant for DDS score (*F*_1, 245_ = 8.94, Cohen *d* = 0.36 [95% CI, 0.12-0.59]; *P* = .003) but not for HbA_1c_ levels (*F*_1, 252_ = 0.29, Cohen *d* = 0.06 [95% CI, −0.17 to 0.30]; *P* = .60). Improvements in DDS scores were modest. There were no differences between EPICC and EUC in improvements after intervention or maintenance for either adherence or self-efficacy. Among all 4002 eligible patients, 280 (7.0%) enrolled in the study (reach). Each clinic conducted all planned EPICC sessions and cohorts (100% adoption). The EPICC group participants attended a mean (SD) of 4.34 (1.98) sessions, with 54 (38.6%) receiving all 6 sessions.

**Conclusions and Relevance:**

A patient-empowerment approach using longitudinal collaborative goal setting and motivational interviewing is feasible in primary care. Improvements in HbA_1c_ levels after the intervention were not sustained after maintenance. Modest improvements in diabetes-associated distress after the intervention were sustained after maintenance. Innovations to expand reach (eg, telemedicine-enabled shared appointments) and sustainability are needed.

**Trial Registration:**

ClinicalTrials.gov Identifier: NCT01876485

## Key Points

**Question:**

Is Empowering Patients in Chronic Care (EPICC), an evidence-based, collaborative goal-setting approach using peer coaching and individual motivational interviewing, effective at reducing hemoglobin A_1c_ levels and diabetes-associated distress among adults in routine primary care settings?

**Findings:**

In this randomized clinical trial involving 280 participants from 5 Veterans Affairs clinics in Illinois, Indiana, and Texas, the EPICC group had significant improvements in hemoglobin A_1c_ levels at 4 months post intervention, but improvements were not sustained at 10 months (maintenance) compared with the enhanced usual care group. Compared with usual care, EPICC demonstrated modest improvements in diabetes-associated distress post intervention that were sustained during maintenance.

**Meaning:**

These findings suggest that a patient-empowerment approach using collaborative goal setting, peer coaching, and motivational interviewing is feasible in primary care clinics and is modestly effective at reducing diabetes-associated distress, although it may not sustain improvements in glycemic control compared with usual care.

## Teaser

This randomized clinical trial evaluates the effectiveness of the Empowering Patients in Chronic Care intervention compared with usual care when delivered by clinicians engaged in routine care of patients with type 2 diabetes.

## Introduction

Type 2 diabetes is a prevalent condition that contributes to adverse outcomes, such as stroke, kidney failure, blindness, and heart diseases.^1^ Guidelines for diabetes control, measured by hemoglobin A_1c_ (HbA_1c_) levels, arise from clinical trials demonstrating lower morbidity and mortality with lowering of HbA_1c_ levels.^2^ Because type 2 diabetes is a chronic condition, achieving control requires patient activation and commitment with treatment planning, medications, and self-management.^3^ Lifestyle changes required to manage diabetes carry an emotional burden contributing to diabetes-associated distress.^4^ Diabetes-associated distress refers to the worries, fears, and threats arising from struggles with chronic diabetes care (ie, management, complications, and loss of function).^5^ Diabetes-associated distress diminishes diabetes self-care and is associated with higher HbA_1c_ levels.^6^

Interventions facilitating communication and collaboration between patients and clinicians that support self-management have the potential for improving diabetes-associated distress and glycemic control.^7,8^ Collaborative goal setting is an evidence-based strategy for improving self-care, trust, and clinical outcomes among primary care patients.^9^ Collaborative goal setting encourages patients and clinicians to share ideas and learn from each other, set patient-defined goals, and support goal achievement.^10^ We developed Empowering Patients in Chronic Care (EPICC) as a collaborative goal-setting intervention using coaching plus individual motivational interviewing to activate patients to explore what matters most,^11,12^ set measurable goals based on what matters,^13,14^ develop skills to communicate goals with clinicians,^8^ and negotiate action plans to achieve their goals.^15,16^ In a clinical trial comparing EPICC with usual diabetes care plus diabetes and nutrition education,^17^ participants in the EPICC group had significantly greater improvements in HbA_1c_ levels after enrollment. Improvements among participants in the EPICC group, mediated by enhanced self-efficacy, persisted at 12 months.^17^

Implementing health systems interventions, such as EPICC, that engage patients in routine primary care is challenging owing to administrative burdens, time constraints, and economic disincentives.^18^ Integrated health systems using interprofessional, team-based primary care can overcome these challenges.^19^ Within this context, we partnered with regional Veterans Affairs (VA) health networks to conduct a hybrid effectiveness-implementation study of EPICC within 5 primary care clinics. This study evaluates the effectiveness of EPICC when delivered by clinicians engaged in routine diabetes care.^20^ The present study evaluated (1) the clinical effectiveness of EPICC to improve diabetes control and reduce diabetes-associated distress and (2) the implementation of EPICC as a routine practice across several sites. We hypothesized that patients who received EPICC would experience significant improvements in HbA_1c_ levels and diabetes-associated distress compared with enhanced usual care (EUC) and would sustain improvements during a 6-month maintenance period. We also hypothesized that participants who could engage in more EPICC sessions (higher fidelity) would experience greater improvements in the primary clinical outcomes.

## Methods

### Study Design

This randomized clinical trial was conducted from July 1, 2015, through June 30, 2017, among patients with treated but uncontrolled type 2 diabetes (the trial protocol is available in Supplement 1).^21^ The VA central institutional review board and each clinic-based research and development committee approved the protocol. All participants provided verbal informed consent by telephone. The study conformed to the Consolidated Standards of Reporting Trials (CONSORT) reporting guideline. We completed follow-up by November 30, 2018, and final analyses by June 30, 2020. All analyses were based on intention to treat (ITT).

The study used a hybrid randomized trial design to evaluate EPICC effectiveness on diabetes outcomes in the context of implementation within 2 regional VA health systems.^22^ We first developed a research-practice partnership with practice leaders and 20 nonacademic health care professionals (ie, dietitians, nurses, pharmacists, and physicians)^23^ who provided usual care from 3 hospital-based and 2 community-based primary care clinics in Illinois, Indiana, and Texas to deliver EPICC within routine care. Partnership building facilitated practice and partner recruitment, training and validation of clinicians in EPICC protocols, and implementation within care workflows.^20,23^ We then randomized enrolled patients to a 3-month intervention with EPICC or to EUC, comparing the primary outcomes of postintervention change in HbA_1c_ levels and diabetes-associated distress and sustainment of treatment effects during a 6-month maintenance period.^24^

### Participants and Eligibility Criteria

**Figure 1.**
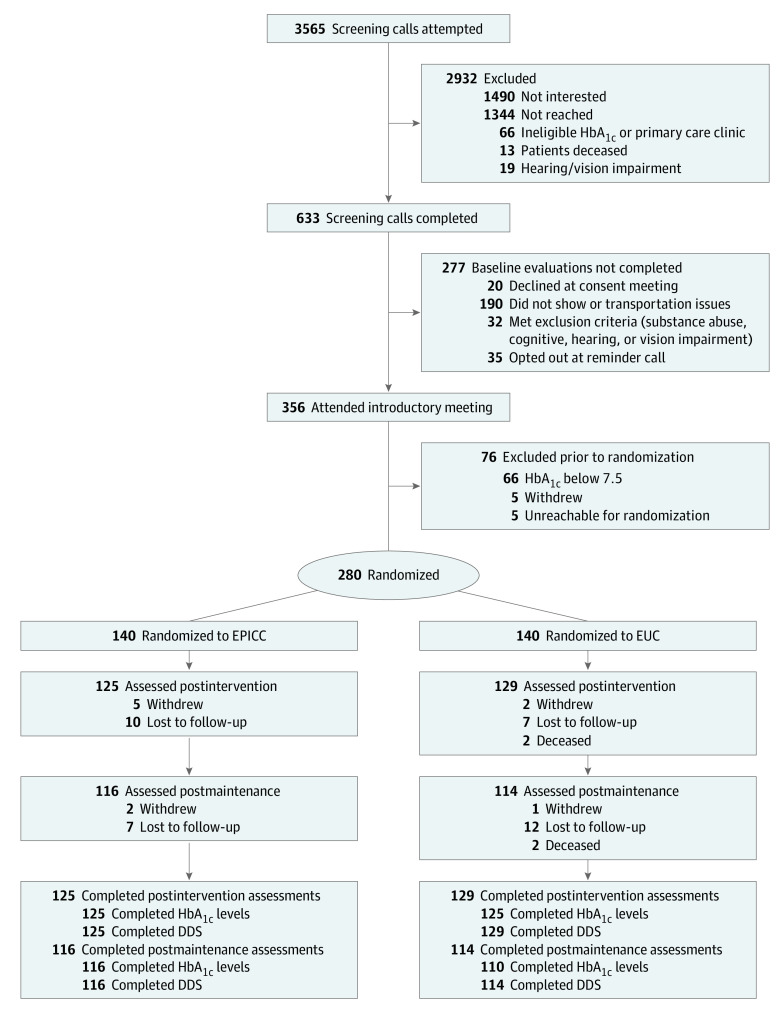
Study Flow Diagram DDS indicates Diabetes Distress Scale; EPICC, Empowering Patients in Chronic Care; EUC, enhanced usual care; and HbA_1c_, hemoglobin A_1c_.

To enhance intervention reach, we used the VA’s Corporate Data Warehouse to conduct a broad population screen for eligible patients (Figure 1). We sent invitation letters to 4198 participants with uncontrolled type 2 diabetes (defined by *International Classification of Diseases, Ninth Revision*, diagnosis code 250.XX or *International and Statistical Classification of Diseases and Related Health Problems, Tenth Revision*, diagnosis code E11.XX, with a mean HbA_1c_ level >8.0% in the prior 6 months [to convert to a proportion of total hemoglobin, multiply by 0.01]) who received primary care at participating clinics in the previous year. Exclusion criteria consisted of hearing or vision impairment, active substance use disorder (within 1 year), active bipolar or psychotic disorder, dementia, severe hypoglycemia (defined as a glucagon prescription), limited life expectancy (identified using a validated algorithm), or death.^25^ Among those who did not opt out, we called 3565 patients to assess interest and screen for conditions that limit participation with in-person group interactions (ie, hearing or vision loss, transportation barriers, significant cognitive impairment, or active substance abuse).^26^ Among those screened, 356 presented for an introductory meeting and baseline data collection. We excluded participants if their baseline HbA_1c_ level was less than 7.5% or if they were unwilling to participate in regular group sessions. We randomized the remaining 280 participants.

### Randomization and Blinding

To assign participants equally to EPICC or EUC, we randomized patients equally by site within random blocks of 4, 6, or 8 generated by using the ranuni function in SAS, version 9.4 (SAS Institute Inc). We chose to keep our block sizes small and use random sequences of block sizes to produce more balanced groups without augmenting the risk that the allocation process would be predictable. The project coordinators assigned participants to interventions. Clinicians delivering the intervention knew the participants’ assigned arm. However, the research assistants who enrolled participants and collected postintervention and maintenance data were blinded to assigned arms. At each time point, blinded staff scheduled participant follow-up assessments and HbA_1c_ measurements using standardized methods at local VA clinical laboratories.

### Study Arms

EPICC participants attended 6 bimonthly group sessions (duration of approximately 1 hour) based on collaborative goal setting and motivational interviewing theory during a 3-month period. eFigure 1 in Supplement 2 details the session structure and themes. A 3-hour training workshop prepared health care professionals (physicians, nurse educators, nurse practitioners, pharmacists, dietitians, and psychologists)^21^ to lead sessions and conduct 10-minute individual sessions immediately following group sessions with each participant. During the individual sessions, participants discussed their personal concerns and questions, set and adjusted collaborative goals, and reviewed changes to medications or other recommended care. EPICC-trained clinicians and participants used guidebooks throughout the intervention. Participants randomized to EUC received routine care that included diabetes management educational materials, nutrition counseling, medication management or weight loss support, a list of self-management resources routinely offered at their site (eg, traditional diabetes education), and communication with their primary care clinician indicating the desire for additional diabetes resources.

### Outcomes

We used RE-AIM (reach, effectiveness, adoption, implementation, and maintenance) to guide analyses for this study.^24^ Primary and secondary outcomes evaluated the clinical effectiveness of EPICC 4 months after enrollment (post intervention) and maintenance of intervention effects during the subsequent 6 months. For effectiveness*,* we measured change in HbA_1c_ levels (primary), diabetes-associated distress (secondary), adherence (secondary), and self-efficacy (secondary) from baseline to the postintervention evaluation. For maintenance*,* we examined whether change was maintained 10 months after enrollment (ie, during the 6-month period after the intervention). Levels of HbA_1c_ were measured at participating clinics’ respective laboratories using a standardized method of ion-exchange liquid chromatography. The Diabetes Distress Scale (DDS) is a validated, patient-reported scale for measuring distress attributable to diabetes care.^27^ The DDS is a 17-item instrument with high internal consistency, reliability (Cronbach α = 0.93), and correlation with self-care behaviors (*r* = 0.30 [*P* < .001]) and physical activity (*r* = 0.13 [*P* < .01]).^27^ Higher scores indicate greater reported distress. A DDS score greater than 2.0 (moderate distress) was considered clinically significant. The Morisky Medication Adherence Scale is an 8-item measure of medication adherence. Each item measures specific adherence behaviors of the respondent. The sensitivity is 93% (Cronbach α = 0.83). The Lorig Self-efficacy Scale is an 8-item instrument (Cronbach α = 0.83) that measures confidence in performing specific diabetes management tasks, with validation as a moderator of change in HbA_1c_ levels.^17^

Secondary implementation outcomes evaluated the remaining RE-AIM dimensions: (1) reach was the number of eligible patients who participated in the study; (2) adoption was the proportion of actual vs planned EPICC sessions conducted by trained clinicians; and (3) implementation was the number of group sessions attended per patient and how this number is associated with primary outcomes. We optimized fidelity to the EPICC protocol by audio recording all initial group sessions as well as a random 20% of subsequent sessions for review by the EPICC trainer. All clinicians had good adherence to the EPICC protocol and competency delivering the intervention.^21^ Using the VA Corporate Data Warehouse, we collected data on use of health care services (ie, number of primary care visits, hospitalizations and length of stay, and emergency department use) as exploratory outcomes comparing participants in the EPICC vs EUC groups from 4 months before enrollment to 10 months after enrollment.

### Power Calculations

To ensure 80% power to detect a small to medium between-group effect size of Cohen *d* = 0.40 at a 2-tailed α = .05 indicating statistical significance, we targeted 284 participants (equally randomized to EPICC and EUC). We accounted for dependency within groups and up to 15% attrition during the maintenance period.

### Statistical Analysis

Before conducting outcome analyses, we compared study completers, defined as those who completed the self-reported DDS at the maintenance assessment, with study noncompleters on pretreatment demographic variables and clinical characteristics using χ^2^ tests and independent-samples *t* tests. We then compared treatment arms (EPICC vs EUC) on the same variables using χ^2^ tests and independent-samples *t* tests. Variables that differed at baseline were subsequently included as covariates. To determine whether outcome models should account for dependency of patients within sites and/or cohorts, we examined intraclass correlation coefficients for each HbA_1c_ level and DDS score for site and cohort. Intraclass correlation coefficients of 0.05 or greater indicate sufficient between-group variance and warrant inclusion of the higher-level unit in multilevel models.^28^ The intraclass correlation coefficients for site were 0.12 for the DDS score and 0.35 for HbA_1c_ levels. The intraclass correlations for cohort were 0.13 for the DDS score and 0.02 for HbA_1c_ levels. Therefore, multilevel models accounted for dependency of patients (level 1) within cohorts (level 2) and sites (level 3).

Outcome analyses first examined group differences in primary and secondary outcomes after the intervention using PROC MIXED in SAS, version 9.4. For HbA_1c_ level, DDS score, adherence, and self-efficacy, the value after the intervention was the dependent variable, the treatment group was the independent variable, and the respective baseline value of the outcome served as a covariate. Analyses were based on ITT using PROC MI and MIANALYZE multiple imputation procedures in SAS, version 9.4, to address missing data. Analyses were then repeated to compare group differences in primary outcomes in the maintenance period. For HbA_1c_ level, DDS score, adherence, and self-efficacy, the value at maintenance was the dependent variable, treatment group was the independent variable, and the respective baseline value of the outcome served as a covariate.

Secondary analyses to address reach, adoption, and implementation were conducted within the EPICC subgroup*.* These analyses were generally descriptive in nature (ie, means [SDs] or frequencies [percentages]). To examine associations between the total number of group EPICC sessions attended and the outcomes of HbA_1c_ level and DDS score post intervention, 2 multilevel ITT models (using PROC MIXED as well as PROC MI and MIANALYZE) were conducted. The postintervention value was the dependent variable, the number of group sessions attended was the independent variable, and the respective baseline value of the outcome served as the covariate. We repeated analyses for HbA_1c_ levels and DDS scores with maintenance outcomes as dependent variables. We also conducted an exploratory analysis within the EUC arm to examine the correlation of change in HbA_1c_ level among participants in the EUC group who saw an EPICC clinician vs those who did not during the maintenance period to evaluate for contamination.

We examined group differences in 4 exploratory variables for use of health care services (ie, number of emergency department and/or urgent care visits, primary care clinician visits, hospitalizations, and length of stay [in days]) post intervention and during maintenance using PROC MIXED. The outcome variable for use of health care services either post intervention or during maintenance served as the outcome, with study arm and the baseline outcome value as a covariate.

## Results

### Participant Characteristics

The sample of 280 participants included 264 men (94.3%) and 16 women (5.7%), with a mean (SD) age of 67.2 (8.4) years. Race and ethnicity data were collected by self-report to better describe the relevance and generalizability of the study findings. The sample was diverse, with 107 Black participants (38.2%), 33 Hispanic (11.8%), 134 non-Hispanic White (47.9%), and 6 other (2.1%; including multiple races [American Indian, non-Hispanic White, and other] and not specified). Most participants were married or cohabitating (146 of 277 [52.7%]), had an annual income of less than $40 000 (155 of 258 [60.1%]), and had some college education (210 of 280 [75.0%]) (Table 1). Participants were recruited from 2 community-based (133 [47.5%]) and 3 hospital-based (147 [52.5%]) outpatient clinics. Baseline characteristics were similar between the EPICC and EUC groups with the exception of prior diabetes education, with a greater frequency among participants in the EUC group (93 of 140 [66.4%] vs 69 of 140 [49.3%]; *P* = .004). Overall, 26 participants (9.3%) withdrew, were lost to follow-up, or died during the intervention; another 24 (8.6%) withdrew, were lost to follow-up, or died during maintenance. No participant experienced harm. Participants receiving EPICC and EUC were equally likely (χ^2^_1_ = 0.10; *P* = .76) to be study completers. Study completers at maintenance were similar to noncompleters on baseline demographic and clinical characteristics (eTable in Supplement 2).

**Table 1.** Baseline Characteristics of Participants

Characteristic	Treatment group^a^		
	Total (N = 280)	EPICC (n = 140)	EUC (n = 140)
Site			
Community clinic			
A	79 (28.2)	41 (29.3)	38 (27.1)
B	54 (19.3)	27 (19.3)	27 (19.3)
Facility clinic			
C	64 (22.9)	29 (20.7)	35 (25.0)
D	45 (16.1)	23 (16.4)	22 (15.7)
E	38 (13.6)	20 (14.3)	18 (12.9)
Sex			
Women	16 (5.7)	9 (6.4)	7 (5.0)
Men	264 (94.3)	131 (93.6)	133 (95.0)
Age, mean (SD), y	67.2 (8.4)	67.4 (8.6)	66.9 (8.3)
Race and ethnicity			
Black	107 (38.2)	46 (32.9)	61 (43.6)
Hispanic	33 (11.8)	22 (15.7)	11 (7.9)
Non-Hispanic White	134 (47.9)	70 (50.0)	64 (45.7)
Other^b^	6 (2.1)	2 (1.4)	4 (2.9)
Educational attainment			
High school graduate or less	70 (25.0)	37 (26.4)	33 (23.6)
Some college or more	210 (75.0)	103 (73.6)	107 (76.4)
Annual income, $^c^			
<20 000	80 (31.0)	41 (31.3)	39 (30.7)
20 000-39 999	75 (29.1)	38 (29.0)	37 (29.1)
≥40 000	103 (39.9)	52 (39.7)	51 (40.2)
Employment^d^			
Any employment	40 (14.8)	18 (13.4)	22 (16.2)
Unemployed	214 (79.3)	109 (81.3)	105 (77.2)
Retired or disabled	16 (5.9)	7 (5.2)	9 (6.6)
Married or cohabitating^e^	146 (52.7)	71 (51.1)	75 (54.4)
Living alone^f^	89 (32.0)	44 (31.7)	45 (32.4)
Perceived health score, mean (SD)^g^	3.47 (0.84)	3.45 (0.85)	3.49 (0.84)
Prior diabetes education	162 (57.9)	69 (49.3)	93 (66.4)
HbA_1c_ level, mean (SD), %	9.08 (1.46)	9.11 (1.60)	9.06 (1.32)
Diabetes Distress Scale score, mean (SD)^h^	2.43 (1.03)	2.41 (1.05)	2.45 (1.02)
<2.0 (little to none)	104 (38.1)	58 (42.0)	46 (34.1)
2.0-2.9 (moderate)	97 (35.5)	44 (31.9)	53 (39.3)
≥3.0 (high)	72 (26.4)	36 (26.1)	36 (26.7)
Morisky Medication Adherence Scale score, mean (SD)^i^	3.56 (2.09)	3.53 (2.1)	3.59 (2.1)
Lorig Self-efficacy Scale score, mean (SD)^j^	5.68 (2.3)	5.50 (2.4)	5.86 (2.3)

Abbreviations: EPICC, Empowering Patients in Chronic Care; EUC, enhanced usual care; HbA_1c_, hemoglobin A_1c_.

SI conversion factor: To convert HbA_1c_ to a proportion of total hemoglobin, multiply by 0.01.

^a^ Unless otherwise indicated, data are expressed as number (%) of participants.

^b^ Includes multiple races (American Indian, non-Hispanic White, and other endorsed [n = 4]) and not specified (n = 2).

^c^ Available for 258 participants.

^d^ Available for 270 participants.

^e^ Available for 277 participants.

^f^ Available for 278 participants.

^g^ Scores range from 1 to 5, with higher scores indicating poorer health.

^h^ Available for 273 participants.

^i^ Available for 274 participants. Scores range from 0 to 8, with higher scores indicating lower adherence.

^j^ Available for 275 participants. Scores range from 1 to 10, with higher scores indicating greater self-efficacy.

### Primary Outcome

**Figure 2.**
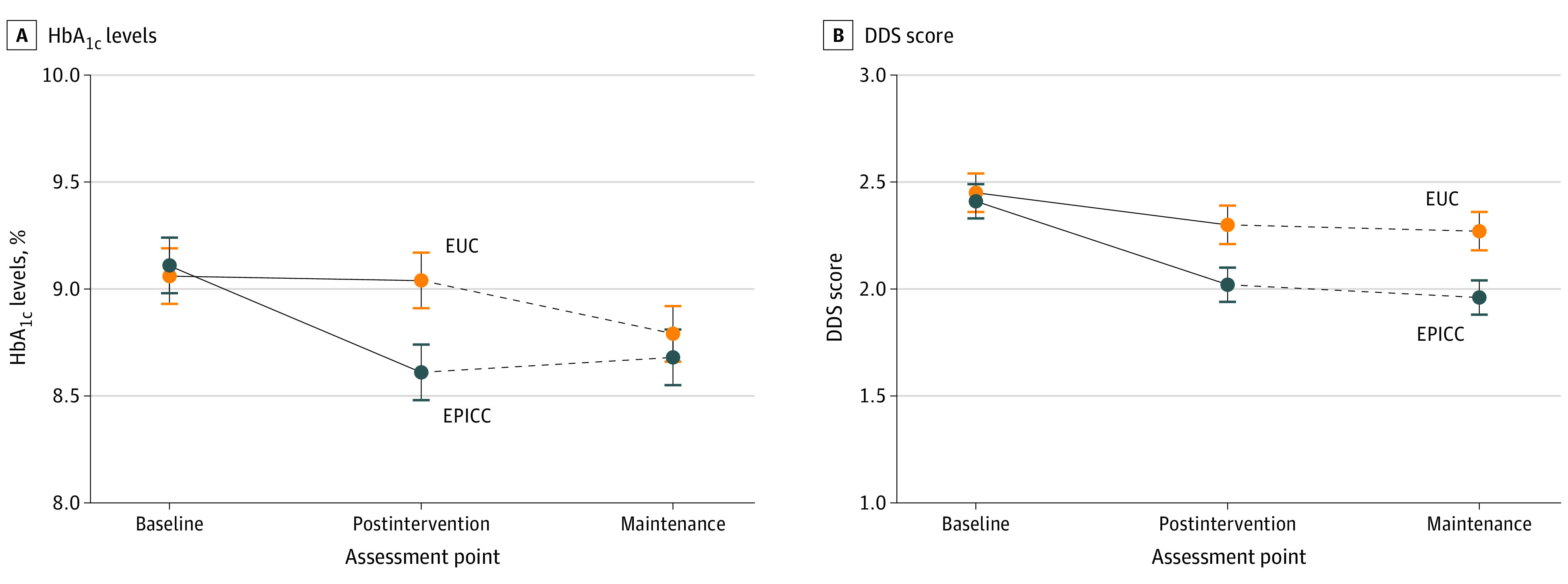
Hemoglobin A_1c_ (HbA_1c_) and Diabetes Distress Scale (DDS) Scores at Baseline, Post Intervention, and During Maintenance A, Among participants in the Empowering Patients in Chronic Care (EPICC) group, mean (SE) HbA_1c_ levels were 9.11% (1.60%) at baseline, 8.61% (1.27%) post intervention, and 8.68% (1.53%) during maintenance. Among participants in the enhanced usual care (EUC) group, HbA_1c_ levels were 9.06% (1.32%) at baseline, 9.04% (1.70%) post intervention, and 8.79% (1.55%) during maintenance. The treatment group effect was significant post intervention (*F*_1, 252_ = 9.12, Cohen *d* = 0.36 [95% CI, 0.12-0.59]; *P* = .003) but not at maintenance (*F*_1, 252_ = 0.29, Cohen *d* = 0.06 [95% CI, −0.17 to 0.30]; *P* = .60). B, Among participants in the EPICC group, mean (SE) DDS scores were 2.41 (1.05) at baseline, 2.02 (0.81) post intervention, and 1.96 (0.76) during maintenance. Among participants in the EUC group, mean (SE) DDS scores were 2.45 (1.02) at baseline, 2.30 (0.99) post intervention, and 2.27 (1.05) during maintenance. The treatment group effect was significant post intervention (*F*_1, 245_ = 9.06, Cohen *d* = 0.37 [95% CI, 0.13-0.60]; *P* = .003) and maintenance (*F*_1, 245_ = 8.94, Cohen *d* = 0.36 [95% CI, 0.12 to 0.59]; *P* = .003). Error bars indicate SEs.

Between-group comparisons of primary outcomes at both postintervention and maintenance are reported in Table 2 and Figure 2A. At the postintervention evaluation, ITT analyses indicated clinically and statistically significant improvements in HbA_1c_ levels among patients receiving EPICC compared with those receiving EUC. Furthermore, at 6 months after intervention completion (maintenance), ITT analyses indicated no difference in HbA_1c_ levels between those completing EPICC and EUC. To examine this finding, we assessed the number of patients in the EUC group who saw an EPICC-trained clinician and found that nearly half (64 [45.7%]) of the participants were seen by an EPICC-trained clinician at least once from postintervention to maintenance. Among the 104 participants in the EUC group with maintenance assessments, a greater number of encounters with an EPICC-trained clinician from postintervention to maintenance was associated with significantly lower HbA_1c_ level at maintenance (*r* = −0.22 [*P* = .02]) (eFigure 2 in Supplement 2).

**Table 2.** Observed Means for Each Outcome Over Time by Treatment Group and Between-Group Comparisons Post Intervention and at Maintenance

Treatment group	Assessment time, mean (SD)				Treatment effect by assessment period			
					Postintervention		Maintenance	
	Baseline	Post intervention	Maintenance	Difference, mean (95% CI)		*P* value^a^	Difference, mean (95% CI)	*P* value^a^
**Primary outcome**								
HbA_1c_ level, %								
EPICC	9.11 (1.60)	8.61 (1.27)	8.68 (1.53)	−0.46 (−0.72 to −0.20)		.003	−0.37 (−0.62 to −0.12)	.60
EUC	9.06 (1.32)	9.04 (1.70)	8.79 (1.55)	−0.04 (−0.23 to 0.15)			−0.28 (−0.55 to −0.02)	
**Secondary outcomes**								
DDS score^b^								
EPICC	2.41 (1.05)	2.02 (0.81)	1.96 (0.76)	−0.39 (−0.54 to −0.24)		.003	−0.41 (−0.57 to −0.25)	.003
EUC	2.45 (1.02)	2.30 (0.99)	2.27 (1.05)	−0.10 (−0.24 to 0.04)			−0.12 (−0.29 to 0.05)	
Morisky Medication Adherence Scale score^c^								
EPICC	3.53 (2.06)	3.13 (1.87)	2.98 (1.80)	−0.35 (−0.61 to −0.05)		.66	−0.48 (−0.85 to −0.11)	.35
EUC	3.59 (2.13)	3.32 (1.70)	3.30 (1.97)	−0.30 (−0.65 to 0.05)			−0.29 (−0.65 to 0.07)	
Lorig Self-efficacy Scale score^d^								
EPICC	5.50 (2.41)	6.50 (2.09)	6.84 (2.08)	0.97 (0.60 to 1.34)		.78	1.19 (0.80 to 1.58)	.32
EUC	5.86 (2.28)	6.57 (2.27)	6.68 (2.04)	0.64 (0.23 to 1.05)			0.85 (0.46 to 1.24)	

Abbreviations: DDS, Diabetes Distress Scale; EPICC, Empowering Patients in Chronic Care; EUC, enhanced usual care; HbA_1c_, hemoglobin A_1c_.

^a^ Each multilevel model controls for the baseline score of the given outcome as well as prior diabetes education.

^b^ Scores range from 1 to 6, with higher scores indicating higher levels of distress.

^c^ Scores range from 0 to 8, with higher scores indicating lower adherence.

^d^ Scores range from 1 to 10, with higher scores indicating greater self-efficacy.

### Secondary Outcomes

Between-group comparisons of secondary outcomes at both postintervention and maintenance are reported in Table 2 and Figure 2B. At the postintervention evaluation, ITT analyses indicated statistically significant improvements in DDS scores among patients receiving EPICC compared with those receiving EUC. Furthermore, at 6 months after intervention completion, ITT analyses indicated continued improvement in DDS scores among patients receiving EPICC compared to those receiving EUC. Although differences between groups were statistically significant both post intervention and during maintenance, effect sizes were small to medium. Furthermore, whereas DDS scores for the EUC group never decreased below the threshold for clinically significant DDS (ie, >2.00), DDS scores for the EPICC group decreased slightly below this criterion after maintenance. Furthermore, there were no differences between those who received EPICC and those who received EUC in either adherence or self-efficacy post intervention and during maintenance (Table 2).

Among all 4002 eligible patients with type 2 diabetes from the target clinics, 280 (7.0%) enrolled in the study (reach). Arising from our partnered implementation approach,^20^ all 5 participating facilities scheduled and conducted sessions as planned for study participants as part of their routine workflows (100% site adoption). Further, patients randomized to EPICC were scheduled for 6 group sessions, with a mean (SD) of 4.34 (1.98) sessions attended (implementation). Most participants (106 of 140 [75.7%]) received at least 4 sessions, with 54 (38.6%) receiving all 6 sessions (Table 3). Excluding the 13 participants who received zero sessions, the number of sessions was associated with improved HbA_1c_ levels during maintenance and improved DDS scores post intervention (HbA_1c_: b = −0.18 [*P* = .04]; DDS: b = −0.11 [*P* = .01]). The number of sessions was unrelated to HbA_1c_ level post intervention (*P* = .56) and DDS score during maintenance (*P* = .11).

**Table 3.** Sessions Each Participant in the EPICC Group Attended and Mean Improvement in HbA_1c_ Level for Each Number of Sessions

No. of sessions	No. (%) of participants (n = 140)	Improvement in HbA_1c_ level, mean (SD), %	
		Baseline to post intervention	Baseline to maintenance
0	13 (9.3)	0.26 (1.44)	0.26 (1.46)
1	7 (5.0)	−0.37 (1.00)	0.45 (1.22)
2	8 (5.7)	−0.57 (1.19)	−0.92 (1.10)
3	6 (4.3)	0.48 (0.88)	0.43 (1.24)
4	18 (12.9)	−0.36 (0.90)	−0.48 (1.55)
5	34 (24.3)	−0.30 (1.02)	−0.32 (1.11)
6	54 (38.6)	−0.57 (1.43)	−0.52 (1.44)

Abbreviations: EPICC, Empowering Patients in Chronic Care; HbA_1c_, hemoglobin A_1c_.

### Exploratory Outcomes

We found no significant differences in hospital, emergency department, or urgent care visits between the EPICC and EUC groups. There was a significantly greater increase in primary care visits from baseline to post intervention for the EPICC group (mean [SD], 2.89 [2.79] visits to 4.53 [3.82] visits) compared with the EUC group (mean [SD], 3.12 [2.71] visits to 2.88 [2.64] visits; *F*_1, 272_ = 22.90 [*P* = .01]; Cohen *d* = 0.57 [95% CI, 0.33-0.81]).

## Discussion

In this randomized clinical trial, an intervention (EPICC) using patient-driven goal setting and motivational interviewing delivered by usual care clinicians lowered HbA_1c_ levels and diabetes-associated distress post intervention among patients with uncontrolled type 2 diabetes compared with EUC. There were no significant differences in adherence or self-efficacy by study arm. At 10 months, participants in the EPICC group maintained modest improvements in diabetes-associated distress compared with those in the EUC group, but HbA_1c_ levels were not significantly different between groups. The narrowing of HbA_1c_ differences between the EPICC and EUC groups may be explained by improvements among participants in the EUC group during the maintenance period. The partnership design facilitated training and delivery of the intervention by usual care clinicians drawn from each participating clinic. Adoption and implementation of the EPICC intervention by clinicians were robust across sites, and adherence to EPICC sessions by participants contributed to improvements in DDS scores post intervention and HbA_1c_ levels during maintenance. Participants in the EPICC group had significantly more primary care encounters but not other health care encounters compared with those in the EUC group.

The EPICC intervention empowers patients to identify what matters most in their lives and transform those values into specific, realistic, and actionable outcome goals.^14^ Clinicians and patients tailor treatments and self-management plans to align with the identified collaborative goals. Aligning care recommendations with patients’ health priorities is an evidence-based approach to improve patient-centered outcomes of adults with chronic illnesses.^29,30,31^ Similarly, the Institute for Healthcare Improvement describes identifying what matters most to older adults as a central pillar to building age-friendly health systems.^32^ In response to Medicare’s advocacy of value-based care, several integrated care systems implemented programs focused on the “whole” patient, shifting from a disease-focused model to one that prioritizes health and well-being.^33^ Within the VA program, system-wide implementation of the whole health approach is transforming care to ensure that veterans receive “personalized, proactive, patient-driven care” to address their physical, emotional, and social well-being.^34^ The present study provides additional evidence supporting the effectiveness of a patient-centered, whole health approach. Further research should evaluate whether a whole health approach helps persons with diabetes achieve a broader range of outcomes that reflect what matter most.

### Strengths and Limitations

A strength of the present study is the hybrid effectiveness-implementation design that builds from partnerships with usual care clinicians at each study site. The study was conducted at 5 clinics drawn from hospital and community settings in 3 states. Training, adoption, and implementation of EPICC among usual care clinicians at involved clinics were robust. A common criticism of using patient-defined goals to guide care is the fear that disease guidelines will be ignored and patients will be exposed to harm and adverse outcomes.^35^ In contrast, the EPICC approach demonstrated improvements in a patient-reported outcome (diabetes-associated distress) and a disease biomarker (HbA_1c_ level) without increases in adverse outcomes such as emergency department visits or hospital admissions.

Despite these strengths, this study has some limitations. Participants were largely male veterans. The VA integrated health system has a patient-centered medical home infrastructure that provides a fertile environment for the EPICC approach that may not be available in other settings. Study participants were not blinded to treatment arm allocation. Due to the limitations of our study design, some diabetes professionals trained to conduct EPICC had exposure to participants in the EUC group during the maintenance period as part of their routine clinical duties. This limitation may have contributed to contamination of EPICC concepts into EUC during the maintenance period. Despite robust adoption by study-enrolled participants (clinicians and patients), overall reach among all eligible patients was low (7.0%).

## Conclusions

The findings of this study suggest that among adults with treated but uncontrolled type 2 diabetes, a patient-centered approach using collaborative goal setting and motivational interviewing may help to reduce diabetes-associated distress while maintaining glycemic levels. These findings also suggest that the EPICC approach is feasible in primary care but requires dedicated resources and staffing, including a variety of health care disciplines that may limit generalizability. EPICC may improve diabetes-associated distress and HbA_1c_ levels post intervention but may only reach a population that can participate in longitudinal group sessions. Future work should explore methods to enhance reach, such as telemedicine-enabled shared appointments and sustainable, empowerment-based approaches.

## Notes

Supplement 1.
Trial Protocol

Supplement 2. **eFigure 1.** Framework for Empowering Patients in Chronic Care (EPICC) **eFigure 2.** Hemoglobin A_1c_ Over Time for Enhanced Usual Care (EUC) by Exposure to an EPICC Clinician Between Postintervention and Maintenance **eTable.** Differences in Baseline Characteristics of Participants by Completer Status

Supplement 3.
Data Sharing Statement
