# Supplementary material for: Comparison of Collaborative Goal Setting With Enhanced Education for Managing Diabetes-Associated Distress and Hemoglobin A1c Levels: A Randomized Clinical Trial
Source: JAMA Netw Open. 2022 May 4;5(5):e229975. doi: 10.1001/jamanetworkopen.2022.9975 (PMC9069258; doi:10.1001/jamanetworkopen.2022.9975)
Supplement: Supplement 1. — Trial Protocol [file jamanetwopen-e229975-s001.pdf]

IIR 12-426: Point- of- care Health Literacy and Activation information to improve diabetes care (Phase 2)

VA HSR&D 12-426

Principal Investigator/Study Chair: **Aanand Naik, MD**

Version Date: July 16, 2019

0.0 Abstract

[Note: The current Central IRB application refers to Phase 2. Grey shaded elements refer to Phase 1 which was approved by the Institutional Review Board for Baylor College of Medicine and Affiliated Hospitals (IRB of record for the Michael E. DeBakey VA Medical Center, Houston, TX) and the Houston VA Research & Development Committee. See Protocol Number H-33772 approval letter (Attachment 1)]

0.0.1. Background: Diabetes mellitus is a highly prevalent chronic condition, affecting one in five Veterans who use the Veterans Affairs (VA) health care system. Self-management skills are critical for controlling diabetes and reducing its cardiovascular sequela. Providing diabetic patients with effective self-management training and support can be challenging due to time constraints at primary care encounters and limited clinician training with behavior change. We have previously demonstrated that a group-based, VA primary care intervention to help patients set highly effective, evidence-based diabetes goals had a positive impact on both diabetes self-efficacy and hemoglobin (Hb) A1c levels. This study aims to evaluate the process of implementing a collaborative goal-setting intervention personalized to patient activation and health literacy levels (i.e. Empowering Patients in Chronic Care [EPIC]) into routine PACT care and to evaluate the effectiveness this intervention relative to usual care.

0.0.2. Objectives: Specific Aim 1: Assess effective processes for and costs associated with implementing a collaborative diabetes goal-setting intervention personalized to patient activation and FHL (i.e., EPIC) into the routine workflows of PACTs. H1: Formative measures within the PARIHS framework (evidence, context, facilitation) will be associated with implementation of EPIC (defined by reach, adoption, cost effectiveness, and fidelity measures) into routine PACT care. Specific Aim 2: Evaluate the effectiveness of delivering collaborative goal-setting personalized to patient activation and FHL on clinical (HbA1c) and patient-centered (Diabetes Distress Scale) outcomes among enrolled eligible patients. H2: Patients receiving collaborative goal-setting personalized to activation and FHL levels will have significant improvements in a) HbA1c and b) Diabetes Distress Scale levels, respectively, post-intervention (4-months) compared with patients receiving enhanced usual care. H3: Patients receiving collaborative goal-setting personalized to activation and FHL levels will maintain significant improvements after a maintenance period in a) HbA1c and b) Diabetes Distress Scale levels at 10 month follow-up, respectively, compared with patients receiving enhanced usual care.

0.0.3. Methods: In Phase 1 of the study, we will conduct a formative evaluation that includes 33-48 key informant interviews with VISN 12 and Houston-based leadership, clinicians, and staff. This evaluation will identify how group and one-on-one sessions of EPIC can best be implemented into routine workflows of PACT. In Phase 2, we will conduct a randomized clinical trial enrolling Veterans with poorly controlled diabetes defined by average hemoglobin A1c of ≥ 8% to receive EPIC or enhanced usual care. To meet a minimum target of 284 Veterans to be randomized for analysis, an estimated population of 428 Veterans will be enrolled, including screen failures, from across participating facilities (approximately 160 from Hines, 200 from Jesse Brown, and 68 between Houston and Lovell). Randomized subjects will be allocated evenly between EPIC and enhanced usual care (EUC). EPIC consists of six 1-hour

groupsessions focusing on 1Your Health, Your Values, 2) Diabetes ABCs, 3) Setting Goals and Making Action Plans, 4) Communication with Your Health Care Provider, 5) Staying Committed to Your Goals, and 6) Reviewing and Planning for the Future. After each group session, a one-on-one session between a designated PACT member and patient participants will focus on collaborative goal-setting. Designated PACT members will be trained to personalize goal-setting using patient-reported activation and health literacy data. We will collect laboratory and survey data at baseline, post-intervention, and post-maintenance phase. We will evaluate the effectiveness of personalized goal-setting compared to enhanced usual care on clinical (e.g., hemoglobin A1c) and patient-centered (e.g., Diabetes Distress Scale) outcomes.

72 List of Abbreviations

AHRQ	Agency for Healthcare Research and Quality
ANCOVA	Analysis of Covariance
Ask Me 3	Patient education program designed to improve communication between patients and health care
Atlas-ti	Qualitative data analysis software
BCM	Baylor College of Medicine
CBOC	Community- Based Outpatient Clinic
CBT	Cognitive Behavioral Therapy
CERT	Center for Education and Research on Therapeutics
CREATE	Collaborative Research and Enhance and Advance Transformation an Excellence Initiative
CSQ-8	Client Satisfaction Questionnaire
DDS	Diabetes Distress Scale
CDW	Corporate Data Warehouse
Delphi	Structured communication technique created by RAND
Deyo	Comparative studies of comorbidity and multimorbidity measures
DSME	Diabetes Self-Management Education
DSM-IV	Diagnostic and Statistical Manual of Mental Disorders
EPIC	Empowering Patients in Chronic Care
EQ-5D	Standardized instrument for use as a measure of health outcome
EUC	Enhanced Usual Care
FHCC	Federal Health Care Center
FHL	Functional Health Literacy
GET-D	Goal-Setting Evaluation Tool for Diabetes
HbA1c	Gylcated hemoglobin
HPDP	Health Promotion and Disease Prevention
HSR&D	Health Services Research and Development
ICC	Intra-class correlation
ICD-9-CM	International Statistical Classification of Disease and Related Health Problems, 9th Revision
ICER	Incremental cost-effectiveness ratio
IIR	Investigator Initiated Research
IRB	Institutional Review Board
ISO	Information Security Officer
IQuEST	Center for Innovations in Quality, Effectiveness and Safety
JBVAMC	Jesse Brown VA Medical Center
MEDVAMC	Michael E DeBakey VA Medical Center
MINANALYZE	Analyze imputations and generates valid statistical inferences

MINI	Short structured interview used to identify mental health conditions
MIRECC	Mental Illness Research, an Clinical Center
MPlus	Statistical Software
ORCA	Organizational Readiness to Change Assessment
P.A.R.T.	Prepared, Ask, Repeat, Take Action
PACTs	Patient-Aligned Care Teams
PAM	Patient Activation Measure
PARIHS	Promoting Action on Research in Health Services
PCP	Primary Care Provider
PEPPI	Perceived Efficacy in Patient-Physician Interactions Questionnaire
PHI	Protected Health Information
PI	Principal Investigator
PII	Personally Identifiable Information
Proc MI	Performs multiple imputation of missing data
Proc Mixed	Enables use of fitted models to make statistical inferences about the data
QUERI	Veteran Affairs Diabetes- Quality and Enhancement Research Initiative
RAND	Research and Development
RCS	Records Control Schedule
RCT	Randomized Control Trial
RE-AIM	Dimensions of Reach, Efficacy, Adoption, Implementation, and Maintenance
REALM	Rapid Estimate of Adult Literacy in Medicine
SAS	Statistical Analysis Systems
SKILLD	Spoken Knowledge In Low Literacy in Diabetes Scale
S-TOFLA	Test of Functional Health Literacy in Adults
SQL	Structured Query Language
UNIX	Multi-user computer operating system
VA	Veterans Affairs
VAMC	Veteran Affairs Medical Center
VINCI	VA Informatics and Computing Infrastructure
VISN	Veterans Integrated Service Network

Contents

Protocol Title: 7
1.0 Study Personnel 7
2.0 Introduction 8
3.0 Objectives 123
4.0 Resources and Personnel 14
5.0 Study Procedures 19
5.1 Study Design 19
5.2 Recruitment Methods 37
5.3 Informed Consent Procedures 39
5.4 Inclusion/Exclusion Criteria 40
5.5 Study Evaluations 41
5.6 Data Analysis 43
5.7 Withdrawal of Subjects 48
6.0 Reporting 46
7.0 Privacy and Confidentiality 50
8.0 Communication Plan 52
9.0 References 55

Protocol Title: Point-of-care Health Literacy and Activation Information to improve Diabetes Care (Phase 2)

1.0 Study Personnel

Principal Investigator/Study Chair:

LeChauncy Woodard, MD, MPH
MEDVAMC, 580
(713) 440-4441 (office)
(713) 748- 7359 (fax)
Woodard.lechauncy@va.gov

Co-Investigators:

Aanand Naik, MD, MS
MEDVAMC, 580
(713) 794-8541 (office)
(713) 748- 7359 (fax)
Aanand.naik@va.gov

Amber Amspoker, PhD
Biostatistician
MEDVAMC, 580
(713) 440-4446 (office)
(713) 748- 7359 (fax)
Amber.amspoker@va.gov

Howard Gordon, MD
Physician
JBVAMC
(312) 569-7473 (office)
Howard.gordon@va.gov

Brian Hertz, MD
Physician
Edward Hines Jr. VA Hospital
(708) 492-3915 (office)
Brian.hertz@va.gov

Commander David Damstra, MD
Physician
James A. Lovell Federal Health Care Center
(847) 688-1900 x 87761
David.Damstra@va.gov

Jennifer Arney, PhD
Qualitative Methodologist
University of Houston Clear Lake
(713) 440-4496 ext: 10374
Jennifer.arney@va.gov

Natalie Hundt, PhD
Research Psychologist
MEDVAMC, 580
(713) 440-4450 (office)
(713) 748- 7359 (fax)
Natalie.hundt@va.gov

2.0 Introduction

Diabetes mellitus affects one in five Veterans who use the Veterans Affairs (VA) healthcare system.[1] Serious cardiovascular diseases, like stroke and myocardial infarction, arise in many diabetic

patients and account for most of the mortality attributed to diabetes.[2] Self-management skills are critical for controlling diabetes and reducing its cardiovascular sequela.[3;4] Providing diabetic patients with effective self-management training and support can be challenging due to time constraints at primary care encounters and limited clinician training with behavior change.[5] We have previously demonstrated that a group-based, VA primary care intervention to help patients set highly effective, evidence-based diabetes goals had a positive impact on both diabetes self-efficacy and hemoglobin (Hb) A1c levels.[6] This study applied collaborative goal-setting theory.[7-9] to empower patients to make diabetes self-management goals and to facilitate goal attainment at subsequent group visits.[6;10] Unlike most educational programs that demonstrate regression to the mean at 4-months, participants in the goal-setting treatment arm sustained HbA1c improvements for nine months after the active intervention.[11] However, ongoing improvements in goal-setting quality were not seen when participants returned to routine primary care and the maintenance of goal-setting activities remained modest at 1-year among intervention participants, suggesting the need to further refine the collaborative goal-setting program.

The effectiveness and maintenance of goal-setting interventions may be enhanced by incorporating VA staff into the collaborative goal-setting process. With appropriate training, existing VA personnel can enhance diabetes outcomes by integrating personalized information about patients' reported self-care capacity (i.e., functional health literacy [FHL]) and motivation (i.e., patient activation measure) into the collaborative goal-setting process.[12;13] In an HSR&D-funded pilot study, we demonstrated that brief measures of FHL and patient activation synergistically predicted HbA1c levels.[14] Thus, assessing patients' FHL and level of activation within the VA PACT context may allow PACTs to better personalize goal-setting among Veterans with diabetes. While validated, practical measures of FHL and activation levels exist; they have not been effectively integrated into routine PACT practice and shown to impact patient outcomes. If such measures were integrated at the point of care (i.e., when primary care providers and patients are developing collaborative diabetes goals), PACT clinicians could personalize goals and action plans within patients' particular limitations and preferences for involvement.

2.A. Background and Conceptual Model

2.A.i. Self-management training and support are key to improving the health outcomes of Veterans with treated but uncontrolled diabetes. At any given time, over one million Veterans are receiving health care services for diabetes, and many suffer adverse vascular outcomes, such as myocardial infarction, blindness and peripheral artery disease.[1] Diabetes control, characterized by reductions in hemoglobin (Hb)A1c, blood pressure, and cholesterol levels, is directly associated with lower morbidity and mortality.[15] Because diabetes is a self-managed condition, achieving diabetes control requires patient involvement in most aspects of treatment planning and management.[16] As a result, self-management training and support is a cornerstone of evidence-based treatment for diabetes in primary care; this practice is endorsed by national standards from the American Diabetes Association,[17] the VA-Department of Defense Management of Diabetes Mellitus Clinical Practice Guidelines,[18] and the VA Diabetes-Quality Enhancement Research Initiative (QUERI).[19]

2.A.ii. Delivering self-management training and support in routine primary care can be difficult, and traditional education programs are handicapped by outdated methods. Most prior self-management interventions have focused on didactic education rather than personalized treatment-planning and development of problem-solving skills.[20] The traditional primary care visit is not an ideal setting to develop or support self-management skills due to time constraints and the need for team-based approaches.[21] The move towards patient-centered medical homes (referred to as Patient-Aligned Care Teams [PACT] within VA primary care)[22] provides an excellent opportunity to efficiently and effectively integrate diabetes self-management training and support into primary care.[23] The goal of VA PACTs is to provide integrated, comprehensive, Veteran-centered primary care tailored to individual characteristics, values, and goals.[22]

2.A.iii. Empirically supported, theory-driven methods of diabetes self-management exist, but more data are needed on wide-spread dissemination and integration into primary care. Collaborative Goal-Setting (Figure 1) is an empirically-supported theory for enhancing human effort, motivation, and persistence toward an outcome. It encourages development of skills and problem-solving strategies for overcoming obstacles when challenges arise.[7;8;24] When adapted to a chronic illness context (main pathway in Figure 1), collaborative goal-setting between patients and clinicians results in greater performance of self-management action plans and improved clinical and patient-centered outcomes.[9;11;25;26] Recent clinical trials have firmly established the clinical effectiveness of diabetes self-management training and support based on goal-setting theory.[6;27-29] However, there is considerable variability across studies, and an Implementation Science approach is needed to resolve gaps in our understanding of how large-scale goal-setting interventions can be effectively implemented into routine workflows and processes of busy health care providers.[11]

One of these critical gaps is how best to integrate self-management training and support into the routine structure of VA PACTs. We have developed and tested a collaborative goal-setting intervention in a trial of two diabetes group clinic interventions: 1) standard diabetes and nutrition education and 2) our collaborative goal-setting approach.[6] The goal-setting approach focused on setting high quality self-management goals and action plans linked to diabetes clinical outcomes (Figure 1). Participants were also taught communication skills to elicit feedback and support about their action plans. The methods used in this study evolved from prior work developing our model of patient empowerment and goal-setting.[30-33] The intervention provided patients with training (group sessions) and support (one-on-one sessions) with diabetes goal-setting. Participants randomized to collaborative goal-setting had clinically significant improvements in HbA1c levels post-intervention and at 1-year follow-up compared to those randomized to the education group. These outcomes were mediated by improvements in self-efficacy related to diabetes self-management tasks.[6]

Figure 1. Theory of Collaborative Goal-Setting

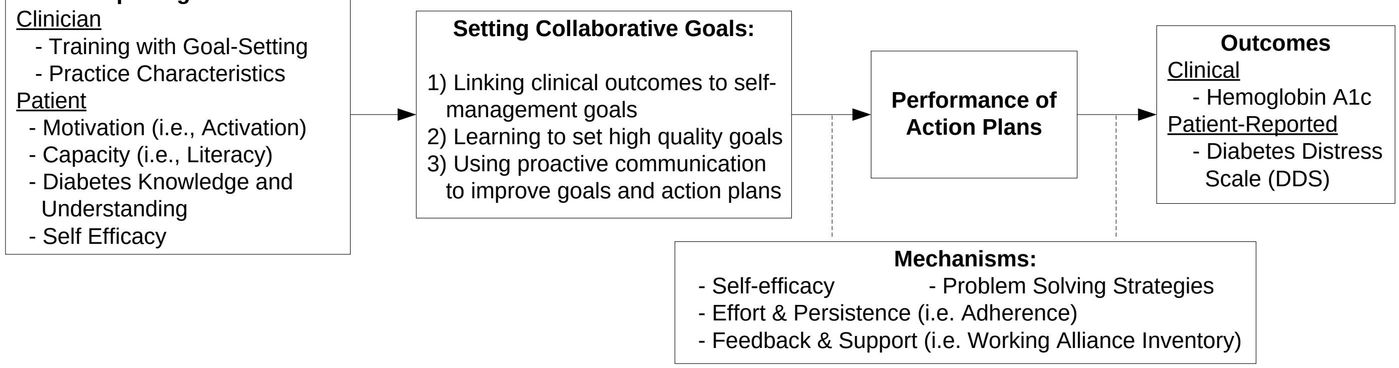

2.A.iii.a Despite these important successes, our prior collaborative goal-setting intervention had limitations. First, ongoing improvements in diabetes self-efficacy and outcomes were not seen when participants returned to routine primary care after the intervention. Second, the maintenance of goal-setting behaviors remained modest at 1-year among participants. These limitations may reflect the fact that we relied on trained research staff to conduct the full intervention and patients' primary care providers had little involvement in the goal-setting process. In additon, the prior study occurred prior to the widespread rollout of PACTs across VA. The proposed study addresses these limitations by implementing the intervention into routine PACT care and using a PACT members to set personalized goals.

2.A.iv. VA PACT realignment creates an opportune setting for involving primary care providers in collaborative goal-setting. The core team or "teamlets" within PACT consist of the Veteran patient, a primary care provider, a nurse care manager, a clinical associate (e.g., licensed practical nurse or health technician) and a clerk.[22] Several teamlets work closely with a larger multidisciplinary team that includes pharmacists, social workers, nutritionists, specialist providers, and staff, including behavioral health specialists. These specialists assist patients with self-management goals and developing problem-solving action plans (i.e., health coaching). Goal-setting and action plans are key elements of effective diabetes self-management.[20;31;34;35] Indeed, the objectives of diabetes goal-setting are completely consistent with the patient-centered mission of PACT. Given realignment of VA primary care towards PACT, dissemination of an evidence-based method for delivering collaborative goal-setting is the right intervention at the right time to improve patient-centered and clinical outcomes for diabetes care.

2.A.v. Further enhancements of collaborative goal-setting can be achieved by integrating personalized information about patients' activation and health literacy levels. The success of goal-setting (see predisposing patient factors in Figure 1) is influenced by patient's motivation (possessing the skills, beliefs, activation and confidence to manage one's health), and capacity (the ability to process and understand basic health information and carry out health decisions). From a conceptual perspective, motivation and capacity can be measured using scales of patient activation and functional health literacy (FHL), respectively.[12;13]

Both FHL and activation play critical roles in achieving diabetes control. Patients with uncontrolled diabetes tend to be passive (low activation levels)[36] and have limited FHL.[37] Studies show that diabetic patients with inadequate FHL are less likely to achieve glycemic control[37] and experience greater difficulty with self-management tasks necessary for diabetes control.[38] Similarly, patients with lower levels of activation also have poorer diabetes self-management and medication adherence.[12] In a prior study, a literacy-focused diabetes intervention was effective in improving glycemic control and self-efficacy in patients with uncontrolled diabetes.[39] Another study found that tailoring self-management coaching to activation levels in diabetic patients was associated with improvements in activation, blood pressure, and low density lipoprotein control.[12]

In an HSR&D-funded pilot study (Woodard, PI), we demonstrated that brief measures of FHL and patient activation can be elicited among diabetic patients, and those with high scores on both measures had significantly lower HbA1c levels ($p<.005$).[14] In another study, our team explored how FHL and activation impact preferences for collaborative decision making among chronically ill Veterans and demonstrated that these preferences are potentially mutable when clinicians consider FHL.[40] Given these findings, personalizing diabetes goal-setting using **both** activation and FHL is an important next step in improving collaborative goal-setting between patients and PACT members. We anticipate that addressing both activation and FHL will have a synergistic effect, leading to higher quality goals, action plans and ultimately, better diabetes outcomes.

2.A.vi. Delivering FHL and activation information and training PACT members to personalize goal-setting using this information can improve diabetes outcomes. Health care providers frequently have difficulty identifying patients with limited FHL;[41;42] therefore, delivering information about FHL to providers during patient-provider encounters may enhance communication and decision-making. However, work in this area is limited. In a study by Seligman et al.,[43] physicians who were notified of their diabetic patients' limited FHL prior to a visit reported greater use of strategies to improve communication about disease management, but were less satisfied with encounters due to feelings of inadequacy about using FHL information. Importantly, participating physicians received little education about how to use FHL information to guide interactions.[43] Our team has experience training research and PACT members in the process of collaborative goal-setting,[11] and we are currently testing a telephone delivered intervention with PACT members trained to use goal-setting in Veterans with diabetes and depression (Naik, IIR 10-135). We posit that personalized FHL and activation

information provided at the point of care (i.e., when PACT members evaluate the data and have goal-setting discussions) can improve the effectiveness of goal-setting if PACT members are appropriately trained on how to best integrate this personalized data into the collaborative goal-setting process. Further research is needed to explore the impact of personalized, collaborative goal-setting on clinical and patient-centered outcomes.

2.B. Significance and Relevance to Veterans' Health and the VA PACT Initiative

This study will provide patient-reported FHL and patient activation information to PACT members to improve collaborative goal-setting in patients with treated but uncontrolled diabetes and ultimately, improve clinical and patient-centered outcomes.

2.B.i. We will use an innovative strategy that brings together three elements to improve the quality and responsiveness of VA PACT care to the needs of over 1,000,000 Veterans with diabetes. First, the study seeks PACT clinical team members' input on barriers and facilitators to the delivery of patient-reported FHL and activation measures to PACTs and then evaluates processes for implementing an innovative diabetes goal-setting intervention personalized to patients' activation and FHL levels across PACTs. Second, the study trains PACT members to use FHL and activation information to better personalize collaborative goal-setting. Most importantly, the study evaluates the clinical effectiveness of this personalized, collaborative goal-setting intervention on clinical and patient-centered diabetes outcomes, relative to enhanced usual care (EUC).

2.B.ii. Our protocol delivers FHL and activation measures at the point of care to personalize collaborative diabetes goal-setting-consistent with the PACT mission. When delivered at the point of care,[44] measures of FHL and activation can influence how PACT members engage in collaborative goal-setting. Considering patient-reported levels of FHL and activation allows for a personalized process of goal-setting, resulting in:

- more specific, personalized feedback shaped by their awareness of patients' activation and FHL,
- higher quality self-management goals and action plans, which in turn promote greater self-efficacy, and
- ultimately, better diabetes clinical and patient-centered outcomes.

This study uses a hybrid type 1 design in which the primary focus is on testing the effectiveness of personalized goal-setting versus enhanced usual care on diabetes outcomes (aim 2), while also collecting some implementation data. Our objective is to test the personalized collaborative goal-setting intervention with a randomized controlled trial (Phase 2) within the constraints of PACT workflows using real-world PACT members instead of research staff. The implementation aim (Phase 1) includes a formative evaluation intended to faciliate integration of the personalized goal-setting intervention within routine PACT workflows and a summative evaluation that measures aspects of implementation. Work on Phase 1 is already underway and is approved under the auspices of the Baylor College of Medicine IRB, the local IRB of record for the Michael E. DeBakey VA Medical Center in Houston, TX.

3.0 Objectives

The overall goals of this hybrid type I effectiveness/implementation trial are to 1) evaluate the process of implementing a collaborative (i.e., patient and PACT member) goal-setting intervention

personalized to patient activation and FHL (i.e., Empowering Patients in Chronic Care [EPIC]) into routine PACT care; and 2) test the effectiveness of this intervention relative to enhanced usual care. In Phase 1(Aim 1), we used the Promoting Action on Research in Health Services (PARIHS) framework to evaluate the feasibility of potential implementation processes into routine PACT care . In Phase 2 (Aim 2), we will assess the effect of delivering personalized goal-setting on clinical (e.g., HbA1c) and patient-centered (e.g., diabetes-related distress) outcomes among Veterans with uncontrolled diabetes. We anticipate that delivering personalized goal-setting involving patients and their PACTs will lead to improvements in diabetes care.

3.B. Specific Aim 1: Assessed effective processes for and costs associated with implementation of a collaborative diabetes goal-setting intervention personalized to patient activation and FHL (i.e., EPIC) into the routine workflows of PACTs.

H1: Formative measures within the PARIHS framework (evidence, context, facilitation) will be associated with implementation of EPIC (defined by reach, adoption, cost effectiveness, and fidelity measures) into routine PACT care.

3.C. Specific Aim 2: Evaluate the effectiveness of delivering collaborative goal-setting personalized to patient activation and FHL on clinical (HbA1c) and patient-centered (Diabetes Distress Scale) outcomes among eligible patients.

H2: Patients receiving collaborative goal-setting personalized to activation and FHL levels will have significant improvements in a) HbA1c and b) Diabetes Distress Scale levels, respectively, post-intervention compared with patients receiving enhanced usual care.

H3: Patients receiving collaborative goal-setting personalized to activation and FHL levels will maintain significant improvements in a) HbA1c and b) Diabetes Distress Scale levels at 1-year follow-up, respectively, compared with patients receiving enhanced usual care.

4.0 Resources and Personnel

4.A. Location of Research, Phase 1 and Phase 2

All data analysis for Phase 1 and Phase 2 will occur at the Houston VA IQuEST (see **§ D 5.6** Data analysis)

4.A.i. Study Team Roles, Phase 1 and Phase 2

<u>Houston, TX Personnel</u>

- <u>LeChauncy Woodard, MD, MPH</u>: (Principal Investigator). Dr. Woodard is a staff Physician at the Houston VA Medical Center and an Assistant Professor of Medicine at Baylor College of Medicine, Houston, TX. She is a core investigator at the Houston VA IQuEST. Dr. Woodard has particular expertise in the design of facility and clinician performance measures as well as methods for enhancing the precision and clinical relevance of performance measurement. This expertise has a strong practical as well as theoretical grounding with Dr. Woodard’s twelve-year partnership with VISN 12. She has used those skills in a VA HSR&D pilot study to identify high-risk primary care patients with co-existing diabetes, hypertension, and ischemic heart disease as well as in her

ongoing quality measurement contract work with VISN 12. As PI of the study, Dr. Woodard will provide primary oversight on all aspects of the project. She will be responsible for the overall research design and implementation, overall project management, lead preparation of project deliverables, assist with the data analysis and interpretation of findings. She will monitor subject recruitment and retention, human subjects' protections and provide intervention and analysis oversight. Dr. Woodard will provide oversight for all aspects of training and supervision of research personnel, conduct project meetings, and be responsible for the scientific progress of the research including manuscripts and reporting of study results. She will have access to protected health information.

- Aanand Naik, MD, MS (Co- Investigator): Dr. Naik is a staff Physician specializing in Geriatrics at the Houston VA Medical Center and an Assistant Professor of Medicine at Baylor College of Medicine. He is a core investigator at the Houston VA IQuEST. Drs. Woodard and Naik have collaborated extensively over the past years evaluating quality of care in chronic diseases. Dr. Naik is currently conducting a hybrid effectiveness-implementation study of a diabetes and depression telehealth intervention also using goal-setting methodology funded by VA HSR&D (IIR 10-135). Dr. Naik also has expertise in applied qualitative research methods. As Co-PI of this study, Dr. Naik will ensure the scientific integrity and overall progress of the goal-setting intervention. Specifically, he will assist Dr. Woodard in all aspects of the study, including recruitment and retention of participants, human subject protections, and intervention and assessment related to diabetes constructs. He provided more direct oversight on the applied qualitative methods and implementation elements in Phase 1. He worked closely with Drs. Woodard, Arney and Amspoker on the data analysis and interpretation of findings for Phase 1. He will provide oversight on the analysis for the summative evaluation of implementation. He will also assist Drs. Woodard and Hundt with training the research staff to conduct the EPIC group sessions.

- Amber Amspoker, PhD (Co-Investigator): Dr. Amspoker is a social psychologist and a member of the Methodology and Statistics Core at the Houston VA IQuEST. She has experience with and knowledge of VA databases and statistical methods. She is highly skilled in using SAS and specializes in database management and analyses. She will be responsible for data management, all analyses, and will materially contribute to manuscript, presentation, and deliverable preparation. She will be responsible for leading the analytical work evaluating the study intervention. She will also assist with the writing of final reports and manuscripts describing the methodological approaches used in this study.

- Natalie Hundt, PhD (Co-Investigator): Dr. Hundt is a clinical psychologist with expertise in behavioral health interventions. She serves as a Co-investigator on a hybrid effectiveness-implementation study of a diabetes and depression telehealth intervention also using goal-setting methodology funded by VA HSR&D (IIR 10-135; PI: Naik). For that project, Dr. Hundt co-developed the patient education materials and the coach training program. She delivers the training, mentors coaches and provides fidelity ratings for the intervention sessions. On this project, Dr. Hundt will use her expertise in behavioral health change to develop the intervention materials, training, and fidelity programs.

- Jennifer Arney, PhD (Qualitative Methodologist): Dr. Arney is an Assistant Professor of Sociology at the University of Houston Clear Lake and has an adjunct appointment with Baylor College of Medicine in the Health Services Research Section. Her primary expertise is in qualitative methods. She teaches qualitative research methods at University of Houston Clear Lake and a mini-course in qualitative research as part of the Education and Training Core's Foundations in HSR curriculum at the Houston VA IQuEST. She provided consultation on qualitative methods (study design,

participant sampling, interview guide development, coding and thematic analysis, and reporting of study results) for Phase 1. She also conducted training of project staff to serve as interviewers and secondary coders on Phase 1 data analysis.

- Lea Kiefer, MPH (Research Coordinator): Ms. Kiefer will be responsible for coordination among the research team, updating research findings, and assisting in the development of materials for presentations, manuscripts or publications. She has a long-standing relationship working with Dr. Woodard as a project manager. She will conduct weekly project meetings and serve as the point of contact for all project-related correspondence. In addition, with Dr. Woodard, she will be responsible for ensuring that the project follows the proposed timeline. Ms. Kiefer will meet weekly with the study team to discuss oversight of the project and as needed with Dr. Woodard between team meetings to discuss other project issues. Ms. Kiefer will be located at the Houston VA IQuEST and supervised by Dr. Woodard. She will have access to the data, including protected health information, and will be involved in recruiting subjects, obtaining informed consent, administering survey/interview procedures, and will be directly involved in the data analysis.

- Sha'Tia Safford, MPH, BA (Research Assistant): Ms. Safford will be sited at the Houston VA IQuEST and will fulfill the local site regulatory responsibilities. Ms. Safford will work directly with Ms. Kiefer to assist with day-to-day recruitment of patients, coordination of phone conferences and meetings, preparation of the adapted EPIC training material for research staff and PACT members, and data collection/entry. Ms. Safford will have access to PHI data during all phases of the study. She will be responsible for developing and implementing an overall recruitment plan for study subjects in the clinical trial as well as recruiting subjects, obtaining informed consent and administering survey/interview procedures. She may assist with dissemination of products.

- Suzette Stine, MBA (Research Assurance & Data Security (RADS) Coordinator): The cost of a research compliance coordinator is shared by all investigators at the Houston VA IQuEST. The coordinator directs, coordinates, and supervises the administrative functions of research compliance at IQuEST. The coordinator audits and monitors all IQuEST research, and aids in the reporting of compliance issues. The coordinator also provides education to investigators and staff regarding regulations, policies, and other VA and federal requirements related to research compliance.

- Alex Chau, BS (Data Management Specialist): Mr. Chau will manage the computing resources needed for timely completion of the project. His duties include hardware and software maintenance and upgrades on Windows servers and UNIX servers, performing backups, and restoring data including disaster recovery on a daily basis on all project folders, and management of user/project accounts, including providing secure accesses to team members. **This is a non-2210 IT employee.**

- Charnetta Brown, MA, BA (Research Assistant) Ms. Brown will be sited at the Hines VA and will fulfill the local site regulatory responsibilities. Ms. Brown will be supervised by Houston staff and work directly with Ms. Kiefer to assist with day-to-day recruitment of patients, coordination of phone conferences and meetings, preparation of the adapted EPIC training material for research staff and PACT members, and data collection/entry. Ms. Brown will have access to PHI data during all phases of the study. She will be responsible for developing and implementing an overall

recruitment plan for study subjects in the clinical trial as well as recruiting subjects, obtaining informed consent and administering survey/interview procedures. She may assist with dissemination of products.

- TBD (Research Assistant): The research assistant to be named will be sited at one of the Chicago-area facilities and will fulfill the local site regulatory responsibilities. The Research Assistant will work directly with Ms. Kiefer to assist with day-to-day recruitment of patients, coordination of phone conferences and meetings, preparation of the adapted EPIC training material for research staff and PACT members, and data collection/entry. The Research Assistant will have access to PHI data during all phases of the study. S/he will be responsible for developing and implementing an overall recruitment plan for study subjects in the clinical trial as well as recruiting subjects, obtaining informed consent and administering survey/interview procedures. S/he may assist with dissemination of products.
- TBD (Research Assistant): The research assistant to be named will be sited at one of the Chicago-area facilities and will fulfill the local site regulatory responsibilities. The Research Assistant will work directly with Ms. Kiefer to assist with day-to-day recruitment of patients, coordination of phone conferences and meetings, preparation of the adapted EPIC training material for research staff and PACT members, and data collection/entry. The Research Assistant will have access to PHI data during all phases of the study. S/he will be responsible for developing and implementing an overall recruitment plan for study subjects in the clinical trial as well as recruiting subjects, obtaining informed consent and administering survey/interview procedures. S/he may assist with dissemination of products.

Jesse Brown VAMC, Chicago, IL Personnel

- Howard Gordon, MD (Co-Investigator): Dr. Gordon is a medical internist and clinician researcher at the Jesse Brown VAMC. He is also Associate Professor of Medicine at the University of Illinois at Chicago and a core investigator at Hines VA HSR&D Center of Excellence. Dr. Gordon has extensive research experience in doctor-patient communication and produced the video that we will use in the EPIC session "How to Talk to Your Doctor". Dr. Gordon will assist the research team with study coordination at the Chicago VA sites and will provide clinical insight during the study related to VISN 12 and study procedures.

Hines VAMC, Hines, IL Personnel

- Brian Hertz, MD (Co-Investigator) Dr. Hertz is the Associate Chief of Staff for Ambulatory Care and a primary care physician at the Edward Hines VA in Hines, IL. He has worked closely with Dr. Woodard for several years on projects examining quality of care in VISN 12. In addition, he has worked closely with Dr. Woodard throughout the development of this project, providing clinical and practical insight on implementing the study in the VISN 12 PACT setting. Dr. Hertz will assist the research team with study coordination at the Hines VA and will continue to provide clinical and implementation insight during the study.

James A. Lovell FHCC, North Chicago, IL

- Commander David Damstra, MD (Co-Investigator): Commander Damstra is a DOD Family Practitioner at James A. Lovell FHCC. He has a WOC appointment with the VA as part of the integrated James A. Lovell FHCC. He has worked closely with Dr. Woodard through development of this project, providing clinical and practical insight on implementing the study in a unique VISN 12 PACT setting (VA/DOD patients). Commander Damstra will assist the research team with study coordination at James A. Lovell FHCC and will provide clinical insight during the study related to VISN 12 and study procedures.

Table 4.A.i.: Summary of Study Team Roles for Phase 2

**Name**	**Location**	**Role**	**Access to PHI?**	**Subject Recruitment and Consent**	**Survey/Interview Procedures**	**Perform data analysis?**
**KEY PERSONNEL**						
Woodard, L.	MEDVAMC	PI	Yes	No	No	Yes
Naik, A.	MEDVAMC	Co-I	Yes	No	No	Yes
Amspoker, A.	MEDVAMC	Co-I, Biostatistician	Yes	No	No	Yes
Arney,J.	MEDVAMC	Co-I	No	No	Yes	Yes
Hundt, N.	MEDVAMC	Co-I	No	Yes	No	Yes
Gordon, H.	JBVAMC	Co-I	Yes	No	No	Yes
Hertz, B.	Hines MVA	Co-I	Yes	No	No	Yes
Damstra, D.	Lovell FHCC	Co-I	Yes	No	No	Yes
**STUDY STAFF**						
Kiefer, L.	MEDVAMC	Research Coordinator	Yes	Yes	Yes	Yes
Safford, S.	MEDVAMC	Research Assistant	Yes	Yes	Yes	No
Stine, S.	MEDVAMC	Assur. Coor.	No	No	No	No
Chau, A.	MEDVAMC	Data Manager	No	No	No	No
Brown, C	Hines MVA	Research Assistant	Yes	Yes	Yes	No
TBD		Clinical Res. Staff	Yes		Yes	No

4.A.ii. Services Provided by Contractors

Not applicable: no contractors were involved in Phase 1 or will be involved in Phase 2.

4.A.iii. Memoranda of Understanding (MOU) or Data Use Agreements (DUA)

Phase 1 required no DUA or MOU. For any databases used in Phase 2 that require Data Use Agreements or Memoranda of Understanding, we will complete all required DUA or MOU paperwork.

Databases that require a DUA include:

- Corporate Data Warehouse (CDW): we will complete DUA to access VINCI

In addition, if a DUA or MOU is needed for use of other databases controlled by VA partners, we will complete that paperwork as well, prior to using the database for research.

5.0 Study Procedures

5.1 Study Design

5.1.A. Overall Study Design

The study will be conducted over four years in two phases. In Phase 1, we implemented our personalized collaborative goal-setting intervention into routine VISN 12 PACT care. To facilitate implementation, we conducted a formative evaluation of VISN 12 PACTs guided by the PARIHS framework. This method of evaluation consisted of key informant interviews with providers, staff, and facility leadership to identify: a) how well PACT members embrace our training/fidelity program for personalized goal-setting (PARIHS evidence), b) how the intervention sessions can be best embedded into routine workflows of PACT at a local level (PARIHS context), and c) local PACT members to assist with recruitment (PARIHS facilitation) of VA staff to conduct the intervention. Phase 1 began during the first year of the study. In the final year of the study, we will also conduct a summative evaluation of overall intervention implementation success based on the RE-AIM measures (see table 5 below) of reach, adoption, and implementation (i.e., cost-effectiveness and fidelity to intervention).

In Phase 2, (see Figure 2) we will conduct a randomized controlled clinical trial to assess the effectiveness of personalized goal-setting in improving clinical and patient-centered outcomes compared with EUC. The unit of randomization will be at the patient level with patients enrolled into the personalized goal-setting intervention (EPIC) versus EUC. Outcome assessments will be conducted at baseline, immediately post-intervention (4 months), and 10 months post-randomization after a maintenance phase.

With the assistance of PACT members and using strategies identified in Phase 1 (formative evaluation), we recruited VA staff who regularly participate in diabetes care to serve as group leaders and individual session providers for the intervention. In Phase 2, we will also recruit and randomize Veterans across eligible facilities to participate in our trial.

Figure 2: Empowering Patients in Chronic Care (EPIC) Study Design (Effectiveness Phase)

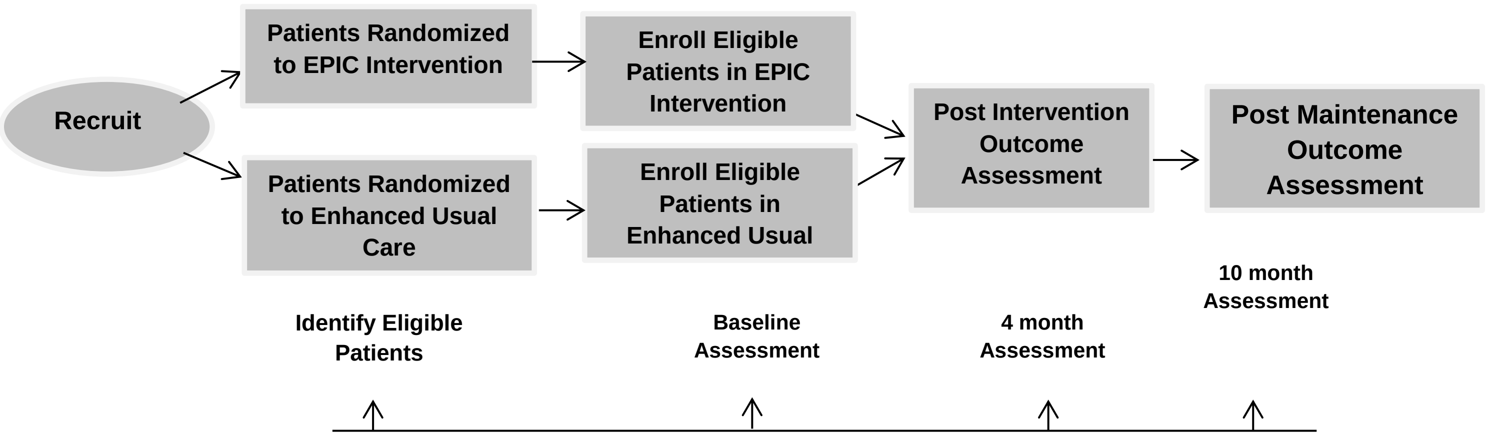

Patient-participants will be recruited from PACT patient panels. Patient panels will be screened for eligibility criteria, and all eligible patients will be approached for informed consent to participate in the study using an opt-out approach and a structured telephone screening and recruitment process.

The study intervention, EPIC (see Figure 3), will consist of six sessions conducted over a maximum of 6 months. Each session will include a group visit followed by a one-on-one personalized goal-setting visit. The personalized goal-setting sessions will incorporate FHL and activation information, allowing the designated study team member to collaborate with patients at their desired levels of engagement to develop diabetes self-management goals.

Patients enrolled in the EPIC intervention will participate in a group session, followed by an individual, collaborative goal setting session. Group sessions will be run by a group leader, a VA staff member who is a regular provider of diabetes care in VISN12. The group leader will be trained to use the EPIC protocol to empower patients in diabetes goal-setting, action planning, and proactive communication with PACT members.[11]

Figure 3. Organization of Personalized Goal-Setting Intervention

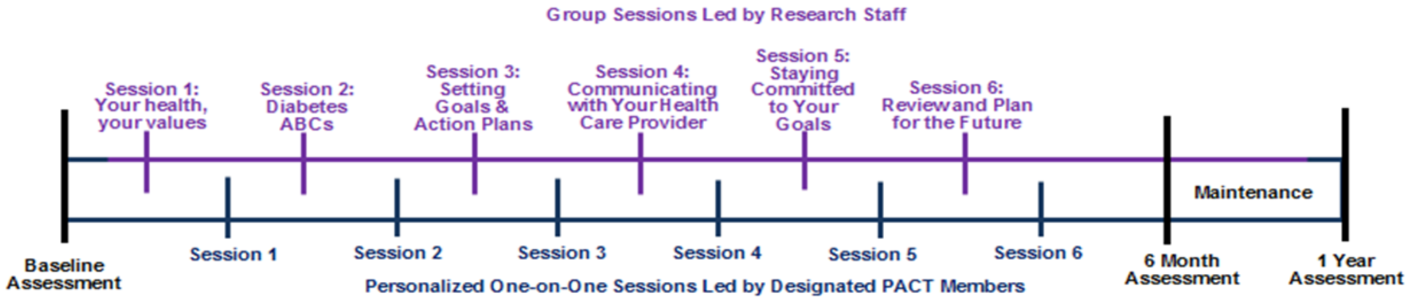

Following the group sessions, participants will receive individual, collaborative goal-setting sessions with an individual session provider, another VA staff member who is a regular provider of diabetes care in VISN12, who is trained by the study staff to lead these goal-setting sessions. Individual sessions will follow the group sessions at a mutually convenient time. The individual session provider will be trained to a) conduct collaborative goal-setting for diabetes self-management and b) understand how to use measures of patient activation and FHL personalized to each patient-participant to enhance the collaborative goal-setting process. Designated VISN12 staffers undergoing EPIC training will be consented as research subjects and must complete an intervention fidelity assessment prior to qualification for the active intervention (**§ 5.1.F.**).

Patients enrolled in the EUC arm will receive routine PACT visits and "enhanced usual care" (EUC). Patients randomized to EUC will be referred to the PACT RN Care Manager for diabetes management , and will also receive a packet of educational materials regarding diabetes management, including a letter delineating the diabetes management resources available at their facility. The PACT RN will be directed to provide care as usual. Patients enrolled in EUC will not receive group or individual goal-setting information defined by the EPIC protocol and their PACT teamlets will not receive personalized information about patient activation or FHL.

5.1.B. Phase 2 design overview

Table 6: VISN 12 facilities	
Facility	Number of patients with HbA1c ≥ 8%
Jesse Brown VAMC Chicago, IL	1133

Lovell FHCC North Chicago, IL	515
Hines VA Hospital Hines, IL	1353
Adam Benjamin, Jr. CBOC, Crown Point, IN	785
Total	3776

In Phase 2, we will conduct a cluster randomized controlled trial with patients serving as the unit of randomization to compare the personalized EPIC intervention with EUC. The EPIC intervention will be delivered by VA staff members who regularly deliver diabetes care, but who are consented as research subjects specifically to collect implementation data on the EPIC intervention.

Using data generated in Phase 1, we have recruited a group of VA staff who regularly participate in diabetes care to serve as the group leaders of the EPIC intervention, as well as the individual session providers., In Phase 2, we will consent and enroll them as research subjects. Following consent, we will train staff on a rolling basis to lead the EPIC group sessions and to perform the personalized, collaborative goal-setting aspects of the intervention. In Phase 2, we will also recruit interested patients to participate in the EPIC trial. Simultaneous with training, we will use the Corporate Data Warehouse to screen VISN 12 patient panels to identify eligible patients using the criteria below (**§ 5.1.E.ii.**). We anticipate enrolling 428 patients for the intervention (including screen failures who do not participate) and 34 VA staff members as group leaders and/or individual session providers. This number is highly feasible given the number of eligible PACTs and patients in our targeted VISN 12 & Houston facilities (see Table 6). A blinded research staff member will collect baseline laboratory, clinical, and survey data at the time of enrollment. An un-blinded research staff member with assistance from PACT staff will schedule patients randomized to the EPIC arm to attend six group clinic sessions. These EPIC group sessions will be conducted by a trained group leader over no more than a six month period. Individual session providers will receive information on FHL and activation for patients assigned to the EPIC group. These providers, who have received training in collaborative goal setting, will then conduct individual personalized goal-setting sessions following each of the EPIC group sessions. The goals and action plans generated during goal-setting sessions as well as any medication-related or other issues raised by patient participants will be communicated to the rest of the PACT team using the preferred methods elaborated in study Phase 1. Providers conducting the individual goal-setting session will work with patients to resolve common issues regarding medications, communicate those issues to the prescribing PACT clinician, and subsequently ensure that modifications to medication regimens are implemented by patients. For subjects randomized to the EUC arm, an un-blinded research staff member with assistance from PACT staff will provide a referral to the PACT RN Care Manager for diabetes management. The un-blinded research staff member will also mail to the patient the EUC materials. A blinded staff member will obtain all clinical and survey data at baseline, post-intervention (4 month follow up assessment) and post-maintenance phase (10 month follow-up assessment) for all enrolled patient-participants.

5.1.C. PACT Setting.

VISN 12 PACT Setting. We will conduct this study in facilities in VISN 12: the Lovell Federal Health Care Center in North Chicago, IL, the Edward Hines VA Hospital in Hines, IL, and the Jesse Brown VAMC in Chicago, IL, including a satellite clinic of the Jesse Brown VAMC, the Adam Benjamin, Jr. clinic in Crown Point, IN. The facilities are located within 50 miles. All facilities have fully implemented PACT. We have targeted two geographic regions (the greater Chicago area and the region of Crown Point, IN) to cluster the organization of our research staff and local PACT members who conduct the EPIC intervention to better ensure implementation success. In addition, we will leverage available resources from our Houston CREATE-VISN 12 partnership, i.e. shared research staff, to facilitate implementation. We will target PACTs with the largest number of eligible patients to maximize recruitment potential.

Houston PACT Setting. Given concern with the availability of staff participants to run the intervention at the approved VISN 12 sites, Houston will serve as an additional enrollment site to ensure that we meet

the approved Veteran sample recruitment size requirement. The Houston VA has fully implemented PACT as well and, because the site serves as the PI/SC site, a supporting research staff is in place.

5.1.D. Study Population

5.1.D.i. EPIC group leaders. The six EPIC group sessions will be delivered by a group leader, a VA staff member who regularly delivers diabetes care. Specifically, the group leaders will be responsible for introducing the concepts in each of the six sessions and for facilitating group discussion; both responsibilities will fall within their normal job duties. These EPIC group leaders will undergo a standardized training program specific to EPIC conducted by the research staff (**§ 5.1.F.**). Each staff member will have time dedicated to complete our training program for the EPIC intervention. Group leaders will participate in fidelity assessments to ensure internal validity (**§ 5.1.F.**). The leaders of the EPIC group sessions at each facility were identified during Phase 1. Diabetes educators and health promotion disease prevention (HPDP) specialists were identified by network PACT leadership as being ideally suited to conduct the intervention. They routinely conduct diabetes self-management classes and are trained in motivational interviewing, which will enhance their effectiveness as leaders of the EPIC group sessions. Given the implementation focus of the research and shifting staffing patterns at each facility, all interested VA staff members at participating facilities who provide diabetes care as part of their regular job duties will be eligible to participate as group leaders.

Prior to training in Phase 2, we will consent and enroll the group leaders as research subjects. Group leaders will be consented as research subjects specifically to collect implementation data on the EPIC intervention. We expect to enroll 3-7 group leaders at each facility, for a maximum total of 34 subjects.

5.1.D.ii. EPIC individual session providers. The collaborative goal-setting sessions designed to follow the EPIC group sessions will be delivered by an individual session provider, a VA staff member who regularly delivers diabetes care. In these individual meetings, staff will assist the Veteran to develop and personalize a self-management goal and an action plan to reach that goal. Individual session providers will be drawn from the local population of staff who have experience with goal-setting and action-planning as a part of the standard diabetes care that they provide. These EPIC individual session providers will undergo a standardized training program specific to EPIC conducted by the research staff (**§ 5.1.F.**). Each staff member will have time dedicated to complete our training program for the EPIC intervention. Individual session providers will participate in fidelity assessments to ensure internal validity (**§ 5.1.F.**).The individual session providers of the EPIC goal-setting intervention were identified during Phase 1 at each facility. Dietitians, pharmacists, diabetes educators and health promotion disease prevention (HPDP) specialists were identified by network PACT leadership as being ideally suited to conduct the intervention. They routinely conduct individual counseling sessions and are trained in motivational interviewing, which will enhance their effectiveness as participants in the EPIC intervention. Given the implementation focus of the research and shifting staffing patterns at each facility, all interested VA staff members who provide diabetes counseling as part of their regular job duties will be eligible to participate as individual session providers.

Prior to training in Phase 2, we will consent and enroll the individual session providers as research subjects. Individual session providers will be consented as research subjects specifically to collect implementation data on the EPIC intervention. We expect to enroll 3-7 individual session providers at each facility, for a maximum total of 34 subjects.

5.1.D.iii. Patient-participants. Inclusion criteria: Using the Corporate Data Warehouse, we will identify active patients at participating facilities meeting the study inclusion criteria: 1) ICD-9-CM codes

indicating diabetes, and 2) average HbA1c level $\geq$ 8% in the prior 6 months. From data preparatory to research, we found a total of 3,776 patients who met those inclusion criteria. All of those records will be screened for the following exclusion criteria to determine eligibility. Exclusion criteria: We will use a medical record review to exclude potential participants with the following clinical conditions that would render participation in a group clinic inappropriate: 1) metastatic cancer or receiving hospice care, 2) limited life expectancy (as identified using a validated algorithm developed in our prior work [see Attachment 2]),[47] 3) clinician recommendations to not titrate therapy due to prior history of significant hypoglycemic events, 4) age <18 years, 5) active bipolar or psychotic disorder, 6) documented active substance abuse, or 7) documented dementia. We estimate that 20% of records will be excluded at chart review, resulting in approximately 3,020 letters sent to Veterans. We will exclude participants at the time of screening who report to study staff that they 6) cannot attend bi-weekly group clinic sessions due to transportation or availability barriers, 7) have significant cognitive impairment (three or more errors on an established six-item screening exam),[63] 8) have active substance-abuse disorders, or 9) are not comfortable discussing their health and health care in a peer-group setting.

Patients will be secondarily excluded if their HbA1C level falls below 7.5% at baseline. Patients whose baseline levels fall below 7.5% may have limited ability for meaningful HbA1c change without significant concerns for hypoglycemia.

5.1.D.iv. <u>Protocol for Randomization into Intervention Groups.</u> Enrolled Veterans will be randomly assigned to EPIC or EUC using random numbers generated in SAS PROC PLAN. We estimate that half of the expected sample of 284 veterans will be randomized to the intervention and half will be randomized to the enhanced usual care arm. We will utilize the steps described below in **§5.2 and § 5.3** to identify, recruit, consent, and enroll patient participants. With the assistance of PACT staff, un-blinded research staff will coordinate the scheduling of participants to EPIC group intervention sessions and EUC referrals.

5.1.E. Study Procedures

5.1.E.i. <u>EPIC Group leader roles and responsibilities.</u> The EPIC collaborative goal-setting intervention consists of six, one-hour group clinic sessions followed by one-on-one, collaborative goal-setting sessions. The intervention is structured to provide patients with training (group sessions) and support (one-on-one sessions) with diabetes goal-setting. Group leaders will be trained by the research staff according to the standardized training program (**§5.1.G**), but will have experience with group diabetes education and/or goal-setting and action planning as a part of the standard diabetes care that they provide. With the aid of the clinician manual (Attachment 3) , group leaders will be responsible for conducting all 6 of the group training sessions over the course of 3 months, but no more than 6 months. When necessary and appropriate, group leaders may also assist Veterans with the development of collaborative diabetes-management goals.

5.1.E.ii. EPIC Individual Session Providers roles and responsibilities. The EPIC collaborative goal-setting intervention consists of six, one-hour group clinic sessions followed by one-on-one, collaborative goal-setting sessions. The intervention is structured to provide patients with training (group sessions) and support (one-on-one sessions) with diabetes goal-setting. Individual session providers will be trained by the research staff according to the standardized training program (**§5.1.G**), but will have prior experience with goal-setting and action-planning as a part of the standard diabetes care that they provide. Individual session providers will be responsible for conducting the one-on-one, personalized goal-setting sessions that will follow each group session at a time of mutual convenience to patient and provider.

Table 7 describes the VA staff involved in conducting the EPIC intervention and their specific roles and responsibilities.

Table 7. PACT personnel roles and responsibilities for EPIC interventions

Personnel	Roles and responsibilities
Group Leaders (with background in diabetes education or health promotion/ disease prevention)	• Participate in EPIC training to cover: 1) review of individual and group EPIC session content and objectives, 2) theory-driven health coaching techniques, 3) setting collaborative goals and action plans, and 4) personalizing goal-setting and action planning based on FHL and activation levels<br>• Conduct the regular group clinic sessions at VA primary care facilities
Individual session provider<br><br>(e.g., dietitian or pharmacist)	• Participate in EPIC training to a) improve collaborative goal-setting skills, b) review patient-reported activation and FHL measures, c) use these measures to personalize goal-setting with patients<br>• Participate in one-on-one collaborative goal-setting sessions with patients randomized to EPIC intervention<br>•
PACT teamlet (e.g. physician, NP/PA, nurse)	• Will play important role in working with EPIC interventionists to integrate patients' goals/action plans with their diabetes treatment plan
PACT clerical staff	• Work with study team to schedule individual and group sessions and order HbA1c tests at 6- and12-months

5.1.E.iii. Procedures for conducting the EPIC intervention. A blinded research staff member will call subjects prior to randomization to collect verbal health literacy and activation information, as well as a short personal history of prior exposure to diabetes management resources. Following that data collection, the randomization status will be revealed to both the Veteran and the research assistant. The research assistant will then explain the next steps for continued participation. Working with VISN 12 PACT clerical staff, un-blinded research personnel will then schedule subjects randomized to the EPIC intervention to attend six group clinic sessions. The groups will consist of 5-8 individuals. The goal is to keep members of a group consistent over the full length of the intervention period to promote peer-to-peer support.[68] Participants in the EPIC intervention will arrive at the facility at the designated group meeting time. They will receive a patient workbook (Attachment 4) at the first session.

EPIC group sessions consist of 6 one-hour group sessions (see Figure 3) occurring over no more than a 6-month period. The group sessions cover the topics described in Figure 4 below (see also Attachments 3 and 4). Group sessions have a consistent structure involving didactic discussion on the topic of interest (20 minutes), a problem-based group discussion (20 minutes), and a group discussion about applying the topic into the patients' lives (20 minutes). Each patient will receive an EPIC manual that guides the content of the group sessions (see Attachment 4). Manuals are designed to ensure that the materials are easily understandable for all participants, including those with limited health literacy.

EPIC one-on-one support sessions will follow each group session. Patient-participants will meet with an individual session provider for 10-15 minutes to personalize goals and action plans. In Phase 1, we developed a menu of 2-3 options that providers can select for conducting the one-on-one sessions (e.g., in-person right after group sessions, in-person at another time, telephone based). Each individual session provider will have the freedom to choose the option that best fits their usual workflow and scheduling process. In preparation for one-on-one sessions, the session provider will receive information on their patients' activation and FHL levels at the start of the intervention. We used the key informant interviews from Phase 1 to inform our process for delivering these patient-reported measures to participating VA staff. In particular, we developed a succinct and actionable format for presenting these data and will train the individual session providers on how to integrate the information into goal-setting (**§ 5.1.F.)**The individual session provider will use this information to better personalize the development of high quality, collaborative goals and action plans. At the conclusion of the individual session, the provider will convey the specified goals and action plans discussed, as well as any medication-related or other issues raised by patient participants to the PACT teamlet via a CPRS note. Given the importance of medication management in the original EPIC study, we developed standardized procedures in Phase 1 for medication management, including medication reconciliation, dose titration, and addition/initiation of alternate medications. The goal is for the individual session provider to work with the Veteran in the course of the individual goal-setting session to resolve common issues regarding medications, communicate those issues to the prescribing PACT clinician, and subsequently ensure that modifications to medication regimens are implemented by patients.

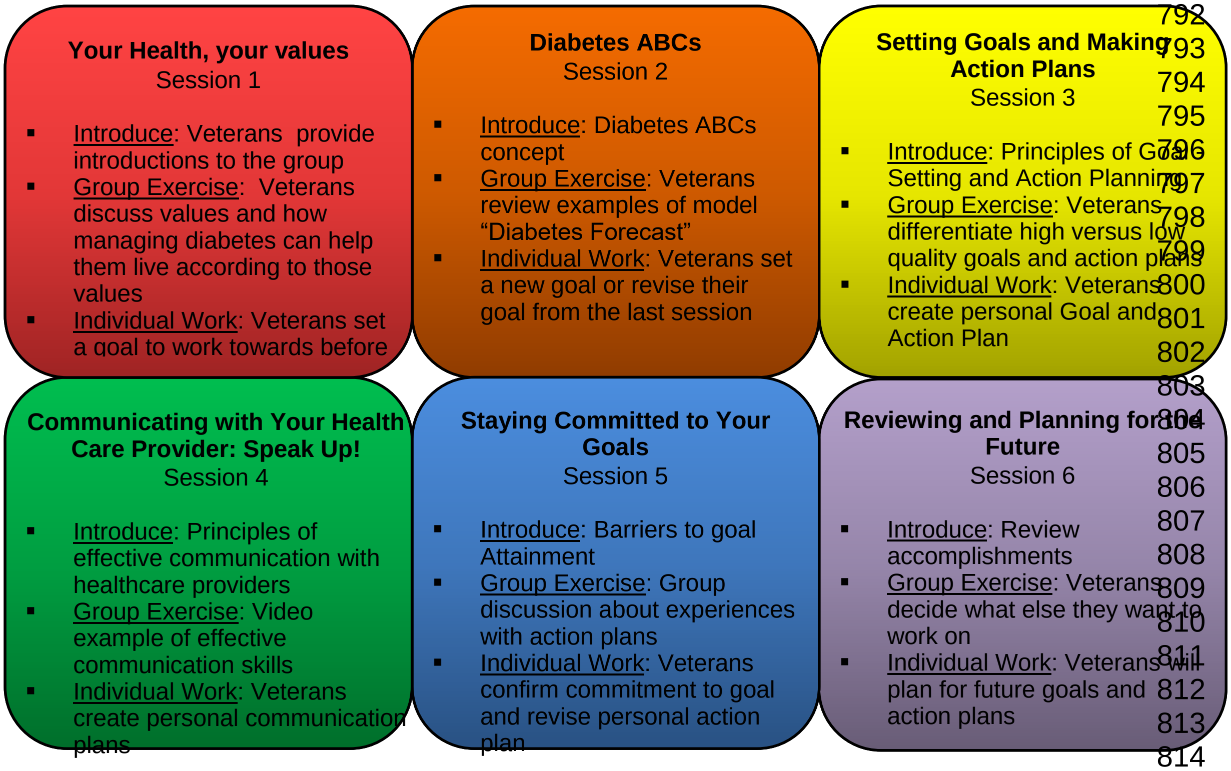

Figure 4. Empowering Patients in Chronic Care Program Structure

Research staff will contact EPIC patient-participants to schedule post-intervention and post-maintenance follow up assessments and HbA1c collection.

5.1.E.iv. Procedures for handling EUC. The full EUC intervention includes:1) a referral to the PACT RN Care Manager,2) a packet of educational materials about diabetes management (Attachment 5), and 3) a letter from the research staff delineating the diabetes management resources

available at their facility and encouraging them to speak to their PACT teamlet about these resources (Attachment 6).

Patient-participants randomized to the EUC intervention will be notified by telephone. A blinded research staff member will call subjects and prior to randomization will collect verbal health literacy and activation information, as well as a short personal history of prior exposure to diabetes management resources. Following that data collection, the randomization status will be revealed to both the Veteran and the research assistant. The research assistant will then explain the next steps for continued participation. After randomization, a mailing to include the educational materials and letter from the research staff will be sent to the EUC patients. Working with PACT clerical staff, unblinded research staff will then refer patients randomized to EUC to the PACT RN care manager for diabetes care management. Research staff will also encourage patients to schedule routine visits with their PACT provider during the six-month active intervention. PACT RN Care managers treating those subjects randomized to EUC will not receive personalized information about activation and FHL levels for their patients.

Research staff will contact EUC patient-participants to schedule post-intervention and post-maintenance follow up assessments and HbA1c collection.

5.1.F. Training of staff personnel to conduct the EPIC intervention

5.1.F.i. Overview of training of EPIC group session leaders and individual session providers. To ensure internal validity, we will train group session leaders and individual session providers to conduct the EPIC intervention. They will be selected from a pool of diabetes care professionals, including education experts and health promotion/disease prevention (HPDP) specialists. We will train each group leader and individual session providers following our established training protocol.[6] The training will cover: 1) intervention objectives; 2) basic clinical skills in motivational interviewing and goal setting; 3) overview of the EPIC protocol; and 3) listening to audiotaped examples of the skills used and participating in role plays and interactive exercises followed by feedback from the study team. At the initial workshop, manuals to guide them through the EPIC intervention (see Attachment 3) will be provided. The manual was designed by our study team and was used successfully in our previous collaborative goal-setting intervention. It contains the contents of the patient manual with specific notations and instructions for leading patients through the group session manual. Following the initial training workshop, the study team will conduct ongoing consultation teleconferences with the group session leaders and individual session providers. The sessions will be led by members of the research team and will focus on reinforcing workshop content and addressing other issues encountered during group sessions and one-on-one goal setting sessions.

5.1.F.ii. Training components The training will include four components: 1) Review of individual and group EPIC session content and objectives; 2) Theory-driven health coaching techniques; 3) Setting collaborative goals and action plans; and 4) Personalizing goal-setting and action planning based on FHL and activation levels. The formal training will last a maximum of 4 hours.

The **first component** provides an overview of EPIC including the overall structure, roles and responsibilities of the group session leaders and the individual session providers , the intervention materials (i.e., patient-participant and clinician manuals), and session objectives. During this session, we will also review the fidelity items on which the designated PACT member will be expected to demonstrate familiarity following the training **(§ 5.1.F.)** and prior to conducting an actual patient session.

The **second component** emphasizes the collaborative coaching nature of goal-setting, including techniques to build rapport and establish trust (e.g., reflective listening, motivational interviewing techniques to resolve ambivalence about change). When combined with goal-setting and action planning (see component three below), use of these techniques is associated with

improvements in clinical parameters including HbA1c, lipid control, and weight loss among diabetic patients.[69-71] Further, this training will capitalize on the motivational interviewing training that is standard for PACTs. We will use the stages of change model to discuss readiness to change and techniques to move patient-participants from one stage of readiness to change to the next stage (e.g., contemplation to preparation or preparation to action) during this component of training.[72;73] To reinforce learning in the context of coaching, trainees will hear audiotapes of brief, scripted vignettes created by our research team and practice these techniques through brief provider-patient role plays.[74;75] Group discussion following role plays will focus on identifying clinical skills appropriate to use in each situation.

The **third component** will focus on how to set high quality collaborative goals and action plans. After participants learn the aspects of high quality goals (i.e., specific, realistic, deadline oriented), they will proceed through goal-setting and action planning role plays with a fellow trainee or local research staff. Following this exercise, the trainer will lead the group in a discussion to clarify the lessons from the role play; this discussion will incorporate the health coaching techniques discussed in training component two. This training sequence has been developed, tested, and modified by Bodenheimer and colleagues[76] to train health professionals in goal-setting and action planning to facilitate diabetes-related behavioral change.

With this foundation, participants will learn strategies to personalize goal-setting and action planning in **the fourth component** of the training session. First, we will introduce the concepts of patient activation, (i.e., possessing the knowledge, skills, beliefs, and confidence to manage one's health) and health literacy (use of "conversational language" (e.g., "sugar" for glucose). We will emphasize how these constructs relate to the patient's motivation to participate in diabetes self-management activities and how to improve communication strategies for patients with low literacy levels. (see Table 8). We will discuss characteristics associated with the spectrum of activation levels ranging from low to high.[12] Patients with low activation are often overwhelmed and not prepared to actively participate in their health care. Conversely, patients with high activation are goal-oriented and have developed effective self-management and problem-solving skills. However, despite high levels of activation, these patients may have difficulty maintaining healthy behaviors when faced with life stressors. Next, participants will learn specific strategies to assist patient-participants at different levels of activation. For example, with lower activation levels, we will instruct participants to focus on single goals that are important to the patient while providing extra encouragement to help build self-confidence, and reinforce the importance of participation. With patients at high activation levels, we will train employee participants to center their interactions with patients on maintaining self-management behaviors, effective problem-solving to prevent relapse, and adding to existing action plans.

Table 8: Patient Activation and Health Literacy Goal Setting Tool

		**Activation** – *Having the knowledge, skill, and confidence for chronic disease self-mana*	
**Health Literacy** - *The ability to perform basic*		**Low Activation**	**High Activation**
	**Low Literacy**	**<u>Description of Veteran:</u>**<br>• Believes someone else will manage diabetes<br>• Has limited ***knowledge*** and ***skills*** regarding self-care and diabetes management	**<u>Description of Veteran:</u>**<br>• ***Ready*** to work on making changes, but ma unsure about what changes to make<br>• May have difficulty understanding comple messages

		• Lacks ***confidence*** in ability to manage diabetes<br>• Focused on the ***present*** more than long term consequences<br>• May have difficulty understanding complex health messages<br>• May suffer from depression<br><br>**<u>Provider Actions to Take:</u>**<br><br>• Ask about what ***motivates*** Veteran<br>• Set smaller, specific ***goals***, walk through steps to achieve goals and reinforce each ***achievement***<br>• Ensure ***understanding*** by asking Veteran to repeat back information<br>• Present essential information first if in written format<br>• Consider referral for depression screening	**<u>Provider Actions to Take:</u>**<br><br>• Ask about what is currently ***motivating*** th<br>Veteran and reinforce positive actions<br>• Help Veteran identify and overcome barri<br>challenges that are preventing self-manag<br>• Evaluate ***knowledge gaps*** by asking patie<br>his or her understanding of diet and medi<br>• Present essential information first if in wri<br>format<br>• Ensure ***understanding*** by asking Veteran t<br>back information<br>• Help patient create tools with visual cues<br>diabetes management (ex: medication ch<br>specific times and pictures instead of phr<br>twice daily)
	**High Literacy**	**<u>Description of Veterans:</u>**<br><br>• ***Overwhelmed*** and lacking in self-efficacy to make changes<br>• Not empowered to gain or use ***knowledge*** and ***skills*** for self-care and diabetes management<br>• Focused on the ***present*** more than long term consequences<br>• May suffer from depression<br><br>**<u>Provider Actions to Take:</u>**<br><br>• Ask about what ***motivates*** Veteran<br>• Set smaller, specific ***goals*** and reinforce each ***achievement***<br>• Ask Veteran how he/she will find new information or develop new skills for care<br>• Emphasize how diabetes can improve the patient's life ***<u>now</u>*** (i.e. more energy, etc)<br>• Consider referral for depression screening	**<u>Description of Veteran:</u>**<br><br>• May have experienced an event or insight<br>convinced him or her to take action<br>• Believes ***diabetes is important*** and that h<br>has the ability to manage it<br>• Has the ***background*** to help learn skills to<br>diabetes<br>• Veteran may be ready for challenging goa<br>his/her expectations may not be realistic<br><br>**<u>Provider Actions to Take:</u>**<br><br>• Ask about what is currently ***motivating*** th<br>Veteran and reinforce positive actions<br>• Help set realistic goals<br>• Ask the Veteran how they will maintain g<br>times of stress<br>• Focus on "***relapse prevention***" efforts. If h<br>has a setback, normalize this and help the<br>restore his or her source of motivation.

To personalize goal-setting and action planning around levels of health literacy (see Table 8), participants will learn widely advocated interactive communication strategies for patients with low literacy levels.[77] Strategies will include the use of “conversational language” (e.g., “sugar” for glucose) and simple techniques such as making eye contact to promote patient understanding. Participants will also learn and practice the “teach back” technique to verify patients’ understanding of the information discussed in the one-on-one sessions.[77] They will be instructed to assess and re-assess understanding until the patient demonstrates comprehension by correctly repeating the content back to the PACT member each time a new topic is introduced or a new goal is set. Using “teach back” has been shown to improve glycemic control among diabetes patients with low literacy levels.[77] To personalize goal-setting based on literacy, participants will learn how to simplify specific goals (e.g., using the plate method vs. reading food labels) within a general category (e.g., diet) for patients with limited FHL (Table 8).

5.1.F.iii. Fidelity measures. We will use three strategies to assess fidelity to the conduct of the EPIC intervention. We used these strategies in our previous trials[79;80] to ensure that the intervention is conducted as intended:

1) Number of treatment sessions: We will track the number of treatment sessions that each patient-participant actually receives compared to the prescribed number of sessions (i.e., six group sessions and six one-on-one sessions). This is the only measure that will be applied to both individual and group sessions.

2) Objective ratings of fidelity along two dimensions: intervention adherence and intervention proficiency. Members of our study team have previously developed and tested a fidelity measure[79; 80] to objectively rate how well an individual has followed a behavioral or self-management support protocol during a one-on-one encounter with a patient. For the current study, the fidelity measure assesses adherence of the participant to the prescribed personalized goal-setting intervention protocol and the participant’s proficiency, or rather, their skillfulness (e.g., building rapport and creating a therapeutic environment) in conducting the group sessions and/or the personalized goal-setting. These ratings are for the purpose of ensuring internal validity to the research. They will not be shared with participants’ supervisors or negatively affect their job in any way. 3) We will also ask patient-participants to provide a self-report of their relationship with the PACT-member conducting their collaborative goal-setting sessions. We will use an Exit Interview survey (modified Client Satisfaction Questionnaire CSQ-8) (see Attachment 7)[81; 82] to determine patient-participants’ perceptions of satisfaction with the service received from the study provider at the last EPIC session. Fidelity ratings of adherence and proficiency have been used in our previous trials along with the CSQ.[81; 82] Greater description of our fidelity ratings and CSQ measurements are provided below **(§5.1.G.a.)**.

5.1.G. Study Variables

5.1.G.a. Fidelity Measures. We will also measure, as described in **§ 5.1.F.iii.**, fidelity to the intervention in the domains of adherence and proficiency. 1. Objective ratings for individual session providers. For individual session provider, adherence and proficiency will be rated after providers have completed the training, prior to the first personalized goal-setting session, in the form of a role-play assessment. Providers who fall below an acceptable level of adherence and proficiency will receive consultation by the study team to address concerns and will be asked to repeat the role-play exercise until an acceptable level is achieved. 2. Objective ratings for group leaders. For group leaders, we will determine adherence ratings based on how closely they adhere to the manual structure and whether or not they cover specific session content. Adherence items will clearly delineate the objectives for each session discussed in the second training component above. Proficiency scores will be based on group leaders’ skillfulness in building rapport with the patient-participant and establishing a therapeutic environment conducive to the development of collaborative goals and action plans (e.g., used language that the patients could follow and understand, answered patient’s questions and concerns). The measure also assesses skillfulness in the use of procedural techniques that are consistent with the

objectives of the intervention (e.g., identified examples and assignments that matched the patient's needs. Group sessions will be audio-recorded when patient-participants agree to allow for fidelity ratings. Research staff will listen live via telephone to those group sessions where consent for audio-recording was not attained by all group participants. Group leaders who fall below an acceptable level will receive consultation by the study team to address areas of concern. No further patients will be assigned to these providers until these individual providers improve. We will provide verbal feedback to staff participants based on performance3. Perceptions of client satisfaction with treatment. Patient-participants will rate their perceptions of client satisfaction with their group leader following the last group session using an exit interview survey, The self-reported paper survey will ask all of the CSQ-8 items (rated on a 4-point Likert scale designed to measure client satisfaction with the services received), as well as additional questions about the EPIC experience. The exit interview survey also asks about interest in future follow up about satisfaction with the EPIC experience to identify a potential sample for future study (see Attachment 7). In addition to overall perceptions of client satisfaction, the exit interview provides a perception of the perceived value of service received; agreement between patient and provider about treatment goals and tasks; and the effective quality of their bond. The CSQ-8 measure has adequate internal consistency and overall scores (Cronbach's α = .92-.93) for 8-item scale.[81; 82]

5.1.H. Data Collection Strategy

Blinded research staff will collect data from patient-participants after all assessments (baseline, post-intervention, and post-maintenance follow-ups). Data to be collected include self-reported measures (see Table 10) and an HbA1c level. Participants will receive $25 for completing the assessment at each time period, for a total of $75 throughout the course of the study.

5.1.H.i. Baseline Data Collection and Assessment. Baseline data collection will occur in person following informed consent at the introductory meeting. A research assistant will be present to distribute the self-reported measures and to answer any questions that participants may have. The self-reported measures will be completed on paper following consent and collected by the research staff. The research staff will review for incomplete measures to guard against missing data. Paper data will be entered by research staff into an Access database for analysis.

Following the introductory meeting, participants will visit the lab to have blood drawn for a baseline HbA1c level. Blinded research staff will coordinate HbA1c collection with PACT team assistance.

Additional verbal baseline measures of functional health literacy, activation and prior exposure to diabetes management resources will be collected by research staff via telephone. These measures will be collected verbally during the randomization call because subjects with limited health literacy may not be able to read or fully comprehend a written measure.[50; 90]

5.1.H.ii. Data Collection at Follow Up Assessments. Post-intervention follow up assessments will be targeted for collection at 5-months after the date of randomization, with assessments occurring no earlier than 4 months after randomization and no later than 6 months after randomization. The assessment following the maintenance phase (Figure 3) will be targeted for collection at 10-months after the date of randomization. Self-reported measures at follow up assessments will be collected by central research staff via telephone using a structured data collection tool. To guide completion of the telephone interview, participants will be mailed blank assessment packets for reference. Blinded research staff will be trained to administer questionnaires by telephone at follow-up assessments and to instruct participants on how to accurately respond to questionnaires. A structured guide will steer participants through response options. We have implemented these procedures in previous studies to

improve data collection and reduce missing data. Patient assessments will not be audio-recorded during the study.

Study staff, working with PACT clerical staff, will schedule a lab visit for HbA1c within 2 weeks of the target data collection time. When a clinical HbA1c lab value is available within the data collection window, it will be used for the research analysis.

Study staff will also perform chart abstraction of patient-level characteristics and clinical or PACT/facility variables that may account for confounding. The patient-level characteristics will include: weight, body mass index, Deyo comorbidity score, receipt of other related treatments (e.g. diabetes education), and primary care visits in the last 12 months.

5.1.H.iii. Attrition/Retention Estimates. Given the benefits of the computerized patient record and our prior experience with VA participants, we expect that rates of missing data for primary outcomes will be <15%. While we may experience a lower adherence with EPIC group sessions, it is reasonable to anticipate having primary outcomes data for ≥85% of participants, as reflected in our sample size and power estimates. To handle missing data, we will conduct sensitivity analyses using tests for data missing completely at random and tests for nonrandom missing-ness. These analyses will allow us to evaluate whether the reasons for loss to follow-up at the various time periods are related to the observed values of the outcome variables. Additionally, we will plot the data over time to visually assess changes in outcomes from baseline to 1-year and to indicate whether additional terms are needed in the models to account for nonlinearity over time.

5.1.I. Study Variables

5.1.I.a. Screening Interview. The screening interview will be conducted over the phone and will identify exclusionary variables by self-report that would render participation in a group clinic inappropriate: 1. Substantial hearing or vision loss, such that participation with the materials and group exercises would not be possible. 2. Transportation or availability barriers, such that would prevent the participant from presenting in person on a regular basis. 3. Unwillingness to discuss their health and health care in a peer-group setting. 4. Cognitive functioning. Cognitive functioning will be assessed using a six-item screening tool that has been validated for telephone use.[83] 5. Current active substance abuse. We will administer modules from the MINI, a short structured interview used to identify mental health conditions including substance abuse according to DSM-IV.[84] It is appropriate for telephone screening (Attachment 9 and 10).[85]

5.1.I.b. Primary Outcomes. 1. Diabetes Control Measure. HbA1c is an established measure of diabetes control and a strong predictor of subsequent health outcomes related to diabetes. There is consensus that levels >7% should be treated because of their association with both cardiovascular risk and microvascular end-organ damage (e.g., kidney failure).[86] Our eligibility criteria of HbA1c of ≥8% at baseline allows for detection of a clinically significant change without limiting enrollment to only those with very poor control or other selective groups. 2. Diabetes-related Distress Scale (DDS). DDS, a17-item instrument that assesses psychological burden specific to diabetes care (see Attachments 7, 8, and 11),[87] has high internal consistency, reliability ($\alpha = 0.93$) and validity with self-care behaviors ($r = .30$, $P < .001$) and physical activity ($r = .13$, $P < .01$). DDS scores correlate with HbA1c levels and are a robust measure of other clinically significant diabetes self-management endpoints.[88] Phase 2 study measures and the data collection timeline are outlined in Table 10.

5.1.I.c. Baseline Covariates. 1. Patient-Level Characteristics (Self-report): We will collect date of birth, gender, race, education, living situation [alone or not], social support, VA copay status, employment status, and prior receipt of related treatments. 2. Patient-Level characteristics will be obtained by chart review and from the Corporate Data Warehouse. (Chart review) A trained research

assistant will conduct a structured chart review to extract data on relevant weight, body mass index, Deyo comorbidity score, receipt of other related treatments (e.g., diabetes education), and primary care visits in the prior 12 months. *(*Corporate Data Warehouse) We will ascertain adherence to refills of prescribed medications (medication possession ratios for all diabetic medications including insulin) for enrolled patients. 3. Health System / Clinic Characteristics*:* We will collect facility, primary care, and PACT characteristics from the Corporate Data Warehouse to account for potential confounding. 4. Patient self-management knowledge and understanding of diabetes will be assessed using a validated 13-item measure that has demonstrated adequate internal consistency (α = 0.68) and correlation with HbA1c values.[89] 5. Patient-reported measures: We will assess levels of FHL and patient activation at baseline. These measures will be collected verbally during the randomization call because subjects with limited health literacy may not be able to read or fully comprehend a written measure.[50; 90] These measures will be reported to the EPIC interventionists and blinded for those in the EUC arm. **A) Functional Health Literacy:** We will use three questions developed by Chew et al and the eight question SKILLD survey, developed by Rothman et al(see Attachment 12).[50; 90; 91] They have been validated across multiple VA samples to correlate with expanded measures of health literacy including the Rapid Estimate of Adult Literacy in Medicine (REALM) and Test of Functional Health Literacy in Adults, short form (S-TOFLA).[50;91] These items require less than three minutes to complete and have been validated among patients with diabetes.[92] **B) Patient Activation:** The Patient Activation Measure (PAM) assesses patients' skill, confidence, and knowledge in managing issues related to their healthcare (see Attachment 12).[12] This 13 item scale can be completed in less than ten minutes. PAM scores have been associated with diabetes outcomes in primary care samples.[36]

5.1.I.d. Predictors and Mediators of Intervention Outcomes. 1. Self-Efficacy for Diabetes Self-Management is an eight-item instrument (Cronbach's α = 0.83) that measures confidence in performing specific diabetes management tasks with a per item mean of 6.87±1.8. It has demonstrated correlation with HbA1c levels.[89] 2. Medication Adherence. We will measure adherence to prescribed diabetes medications using pharmacy refill records from the Corporate Data Warehouse. For each identified medication we will calculate medication possession ratios and refill gaps (See Attachment 7, 8, and 11). We will also capture by self-report the Morisky Medication Adherence scale. This scale allows for identification of patients at highest risk for poor outcomes due to non-adherence as well as recognition of barriers to medication compliance. Responses are scored using a dichotomous scale (yes = 0; no = 1) with higher scores reflecting better medication adherence. The scale has been shown to have good concurrent and predictive validity as well as high internal consistency, indicating good reliability (Cronbach's α = 0.83).[93] 3. Depression Symptoms. The PHQ-8 is an eight-item instrument (Cronbach's α = 0.83) that measures depressive symptoms.[94;95] 4. Exercise. The Lorig Exercise scale is a six item instrument that measures exercise behavior during a typical week. No Internal reliability reported; test re-test for stretching and strengthening r =.56; test-retest for aerobic exercise r =.72.[96] 5. Diet. The Diet scale is a ten-item (Cronbach's α = 0.73) instrument developed as part of the Diabetes Self-Care Activities survey, a 25- item instrument that measures perceived adherence to diabetes self-care recommendations.[97] 6. Goal-Setting Evaluation Tool for diabetes (GET-D) is an objective rater scale developed and validated for scoring the quality of goals and action plans articulated by patients in our prior goal-setting studies (see Attachment 7, 8, and 11). 5. Treatment Fidelity. We will also use a measure, described in **§ 5.1.F.iii.**, to objectively rate staff member fidelity to the intervention.

Table 10. Measures	Screen	Baseline	4 M	10 M	Measure	Baseline	4 M	10 M
Screening protocol	X				Intervention Mediators and Moderators			
Primary outcome variables					PHQ-8<br>Diabetes Self-Care Self-Efficacy	X	X	X
HbA1c levels	X	X	X	X	Diet/Exercise	X	X	X
Diabetes Distress Scale		X	X	X	Pharmacy refills (database)	X	X	X
Baseline Covariates					Goal-Setting Evaluation Tool	X	X	X
Patient Activation Measure		X	X	X	Attendance in group visits		X	X
Functional Health Literacy measure		X			Post-intervention Implementation variables			
The Spoken Knowledge in Low Literacy in Diabetes (SKILLD) Knowledge Assessment Scale		X			Patient exit interviews (Attachment 7)		X	
Patient Socio-demographics		X			Clinician exit interviews (Attachment 13)			X
Baseline clinical characteristics		X			Summative implementation variables			
PACT and facility characteristics		X			Reach and Adoption measures	X	X	X
Patient knowledge & understanding of DM<br>*EQ-5D*		X<br>X	X<br>X	X<br>X	Fidelity measures	X	X	X

5.1.J. Potential Risks

The potential risks of harm to study participants are low for all phases of this study. In Phase 1, the key informant interviews solicited information on how best a) to adapt EPIC to include point-of-care information on patient activation and functional health literacy; and b) to integrate the intervention into routine work flows. The primary risk to clinician and staff participants was loss of time and potential breach of confidentiality.

The risks for staff participating in Phase 2 of the study are also considered minimal for this project because diabetes care is part of their regular clinical duties. There is a small possibility for loss of confidentiality, although participants will be assigned unique, study ID#s, and all analyses will be blinded.

For patient-participants in Phase 2, this trial poses minimal risk; however, there are still some potential risks associated with the proposed tests to assess the impact of the intervention, as well as the intervention itself. Risks associated with the assessments are low given that the items assessed are normal daily activities including blood draws that are conducted as part of the standard of care. There is also a small risk for breach of confidentiality, but patient-participants will also be assigned unique,

study ID#s for analysis and all results will be reported in aggregate. With our eligibility criteria and multi-gated recruitment approach, we should be able to effectively screen-out any individuals for whom this intervention is contraindicated. However, because this intervention aims to improve patients' management of their health through assisting in the implementation of self-management changes, a small risk remains that some patients may experience hypoglycemia after successfully making these modifications. We will closely monitor these potential symptoms and have developed a protocol for interceding whenever hypoglycemia symptoms manifest. We have successfully utilized this protocol in prior studies.

5.1.K. Protection Against Risk

To ensure protection against potential risks, Phase 1 was approved by the Baylor College of Medicine Institutional Review Board and the Michael E. DeBakey VA Medical Center Research and Development Committee. The protocol for Phase 2 will be approved by, the VA Central IRB. In addition, we will obtain approval from the appropriate local VISN 12 and Houston-based VA R&D committees.

The following precautions will be taken with both staff and patient-participants to address possible apprehension with disclosing health care related information. Study participants will be assured that:

- Participation is voluntary;
- They do not have to answer any questions with which they are uncomfortable;
- They can discontinue study participation at any time; and
- Participation will in no way affect the care that patients receive at the VA or employment status for VISN12 or Houston staff members.

In addition, the following precautions will be taken to minimize the risk of loss of confidentiality for all participants in the study:

- All paper patient data will be coded by study ID without identifying information, and any personal identifiable data will be stored separately behind two locks in a cabinet within the PI's office. Access to these files will be restricted to study personnel.
- All electronic data will be maintained on IQuEST's secure and fully backed up UNIX data server, with appropriate ID, password, and data access restrictions.
- All study results and accompanying publications will be anonymous, and will not contain identifiable information.

Some patients may experience clinically significant symptoms of hyper or hypoglycemia during the course of the intervention. We will have protocols for addressing this risk by:

a) First alerting study PIs and then participants' PACT provider when symptoms are more than minor,
b) Assisting participants to develop communication action plans with clinicians when symptoms are mild but warrant discussion with clinicians, and the
c) Reporting significant adverse events to the Institutional Review Board (IRB) when emergent care is required.

Each step will have a protocol specific to each facility's workflows and regulation, and we will then train local study staff, group leaders and individual session providers on the implementation of the protocols accordingly.

Our protocols for hypoglycemia were developed from the original EPIC study as well as adaptations from a current VA MERIT study (PI: Naik) involving behavioral coaching for Veterans with diabetes and depression. These protocols were approved by the PACT leadership at the Michael E. DeBakey VA

and the Houston R&D committee and we will develop a similar procedure for the EPIC study at each participating facility.

5.1.L. Potential Benefits of the Proposed Research and Importance of the Knowledge to be Gained

This study will provide valuable information regarding use of patient-reported measures and a goal-setting intervention integrated into routine care to guide treatment goals and development of action plans to improve care in high risk patients. The use of patient-reported measures of activation and functional health literacy to inform treatment decisions in routine care has not been previously assessed. Further, although the goal-setting intervention that will be adapted for this study has been demonstrated to improve outcomes in a clinical trial setting, it has not been assessed when incorporated into routine care. Thus, we anticipate that the information garnered from this work will not only improve outcomes, but will also inform more patient-centered approaches to chronic illness care.

The study will also generate important data on the readiness, process, and success of implementation of a widely disseminated diabetes collaborative goal-setting intervention and its impact on diabetes outcomes. In addition to facilitating the local implementation of study protocols, we believe important generalizable knowledge will be generated from this work that can be applied to future intervention dissemination and implementation.

As a benefit of participation, patient-participants may also develop skills to set high-quality treatment goals and action plans targeting diabetes self-care. This intervention may potentially improve participants' overall health, and self-management behaviors for diabetes.

Staff participants will be trained in providing effective behavioral health coaching, which will contribute to their professional development and may provide benefit for their clinical practice outside of this intervention.

5.1.M. Protections for vulnerable populations

No potential participant will be excluded based on gender or minority status. Prior work has shown a 2% to 5% recruitment rate for women in this age group. We expect to have a comparable female population for this study. The racial ethnic composition of patients with the study conditions receiving care within VISN 12 is approximately 77% non-Hispanic white, 14% non-Hispanic black, and 9% other. Our goal is to achieve a similar racial ethnic distribution in our study cohort of patient participants.

To guard against any undue influence or coercion by the study on the administrating institution's employee participants, the consent process will emphasize the voluntary nature of the research by including the following statements in the consent form: Participation in this study is voluntary and will not affect your current or future employment status. There is no penalty for refusing to participate and you may withdraw your participation in the study at any time. Additionally, your identifying information and any opinions, insights or information you share will be kept strictly confidential.

Given that our study focuses on diabetes and risk factors for cardiovascular disease in Veterans, children, adolescents and pregnant women will not be included.

5.1.N. Data and Safety Monitoring

The project's data and safety monitoring board will be chaired by Dr. Drew Helmer, Director of War-Related Illness and Injury Study Center at the East Orange VA Medical Center in New Jersey. Dr. Helmer has experience directing PACT teams and will be responsible, along with Dr. Woodard, for directing the data safety and monitoring for the proposed project. Dr Amspoker will serve as project

statistician and methodologist. She will have primary responsibility for preparing the data and safety monitoring plan, ensuring that monitoring is timely and effective, and responding to recommendations and findings that emanate from monitoring activities. Monitoring will be performed throughout the proposed study via quarterly in-person meetings or teleconferences. At each of these meetings, the team will review the status of data collection and monitoring, as well as the clinical status/progress of research participants.

At each quarterly meeting, the project coordinator will provide the following information: number of participants entering the study, status with respect to meeting recruitment targets, percentage of patients assessed who enter the study, number of drop-outs, reasons for dropping out, percentage of patients at each stage of the project, and percentage of assessments completed at each assessment point. Information about any adverse events (including IRB reporting of short- and long-term remedies) also will be presented. By examining this information, the data and safety monitoring team will keep abreast of critical issues regarding recruitment and data integrity.

On a weekly basis, Dr. Woodard will meet with study staff to provide supervision and review the clinical status of all participants. Study staff also will notify at least one supervisor immediately if at any point a patient shows the need for urgent treatment (e.g., hypoglycemic symptoms). This type of information will be communicated immediately, with timely consultation about an appropriate course of action.

Annual feedback will also be provided to the VA Central IRB Data Safety and Monitoring Board, as well as the local Research and Development Committees of participating facilities, including the Michael E. DeBakey VA Medical Center Research and Development Committee.

All unanticipated serious adverse events (U-SAEs) will be reported to the VA Central IRB within five business days. U-SAEs will be reported to VA Central IRB regardless of their relationship to the research. Additionally, all hospitalizations related to a hypoglycemic event (SAE) will be reported to the VA Central IRB within five business days (**§ 6.0**) . All protocol deviations, violations, and/or noncompliance will be reported to the VA Central IRB within five business days of the reporting individual becoming aware of the occurrence.

5.2 Recruitment Methods

5.2.A. Staff participants' eligibility criteria. The leaders of the EPIC group sessions at each facility, as well as individual session providers were initially identified and recruited during Phase 1 which was approved by the local Institutional Review Board for Baylor College of Medicine and Affiliated Hospitals (Protocol Number H-33772). In the event we need to identify and recruit additional interventionalists after Phase 1, we will reach out to recruit eligible providers in VISN 12 and in Houston with an opt-out email (Attachment 17).

Diabetes educators and health promotion disease prevention (HPDP) specialists were identified by network PACT leadership as being ideally suited to lead the group intervention. They routinely conduct diabetes self-management classes and are trained in motivational interviewing, which will enhance their effectiveness as leaders of the EPIC group sessions. These two classes of employees, along with dietitians and clinical pharmacists, were identified as being ideally suited as providers of the collaborative, individual goal-setting sessions. These employees routinely conduct individual counseling and sometimes goal-setting with diabetic patients. Given the implementation focus of the research and shifting staffing patterns at each facility, all interested employees at participating facilities who provide diabetes care as part of their regular job duties will be eligible to

participate as group leaders. Prior to training in Phase 2, we will consent and enroll the staff members as research subjects identified during Phase 1. Group leaders and individual session providers will be consented as research subjects specifically to collect implementation data on the EPIC intervention. We expect to enroll 2-4 group leaders at each facility and 3-6 individual session providers at each facility, for a maximum total of 40 subjects.

5.2.B. Patients' eligibility criteria. Inclusion criteria: Using the Corporate Data Warehouse, we will identify active patients at participating facilities meeting the study inclusion criteria: 1) ICD-9-CM codes indicating diabetes, and 2) average HbA1c level $\geq$ 8% in the prior 6 months. We will not use preparatory to research data. We will conduct a data search under approved waivers to identify eligible patients. Exclusion criteria: We will use a medical record review to exclude potential participants with the following clinical conditions that would render participation in a group clinic inappropriate: 1) metastatic cancer or receiving hospice care, 2) limited life expectancy (as identified using a validated algorithm developed in our prior work [see Attachment 1]),[47] 3) clinician recommendations to not titrate therapy due to prior history of significant hypoglycemic events, 4) age <18 years, 5) active bipolar or psychotic disorder, 6) documented active substance abuse, or 7) documented dementia. We estimate that 20% of records will be excluded at chart review, resulting in approximately 3,020 letters sent to Veterans. We will exclude participants at the time of screening who report to study staff that they 8) have substantial hearing or vision loss, such that participation with the materials and group exercises would not be possible,9) cannot attend bi-weekly group clinic sessions due to transportation or availability barriers, 10) have significant cognitive impairment (three or more errors on an established six-item screening exam), [63] 11) have active substance-abuse disorders, or 12) are not comfortable discussing their health and health care in a peer-group setting.

Patients will be secondarily excluded if their HbA1C level falls below 7.5% at baseline. Patients whose baseline levels fall below 7.5% may have limited ability for meaningful HbA1c change without significant concerns for hypoglycemia.

We will notify all participants identified as having uncontrolled diabetes but who do not meet the final eligibility criteria (i.e., whose HbA1c drops below 7.5% at baseline) of their results. A note will be placed in their medical record indicating this finding and they will be withdrawn from the research.

5.2.B.i. Identification of Patient- Participants and Recruitment Strategies.

1) Identify potentially eligible patient-participants in VISN 12 and Houston using data from the Corporate Data Warehouse. To ensure accurate ascertainment of diabetes diagnosis, we will identify patients with at least 2 outpatient or 1 inpatient ICD-9 code for diabetes mellitus. We will extract HbA1c values from the prior 6 months. Patients with mean HbA1c $\geq$ 8.0% will be eligible for Step 2.
2) We will perform a standardized medical-record review to verify the diagnosis of diabetes and evidence of any exclusion criteria. We will use a step-wise approach to the medical-record review, adapted from our prior work, in blocks of 100 patients. Patient blocks will be organized by PACT team. We will send opt-out letters (Attachment 15) to patients that remain eligible for study participation. To ensure timely responses to patients and realistic work load, opt-out letters will not be sent until we have attempted to contact 3/4ths of the prior block sample.
3) We will then recruit all potentially eligible patients via an opt-out letter sent on behalf of the PACT team mailed to their home address. Letters written at a sixth-grade reading level will direct patients to call an opt-out number if they do not wish to be contacted about the study. A toll-free telephone number answered by voice mail will be available for those with questions or who want to leave an opt-out message. Unless the patient requests that he/she not be contacted, research personnel will contact the patient after ten days or after the first telephone response from the same batch of letters is received, whichever comes sooner. This protocol was previously approved by VA Institutional Review Boards.

4) We will then call potential subjects to introduce the study objectives and procedures and to obtain verbal consent to administer a screening protocol. All potential participants who express interest in the study and who do not meet the exclusion criteria will be invited to a group introductory meeting. Time permitting, a letter detailing the date, time and location of the introductory meeting will be mailed to the patient (Attachment 16).Research staff will provide a reminder call to invited patients before the introductory meeting to ensure adequate group numbers and to answer any remaining questions or concerns in a private conversation.
5) The full informed consent process will be performed at the introductory meeting. Following consent, the baseline paper surveys will be completed by participants.
6) Baseline HbA1c lab draws will be ordered for immediately following the introductory meeting. If the values for the baseline HbA1c level fall below 7.5%, patients will no longer be eligible for randomization and will be withdrawn. Patients who still meet eligibility criteria (i.e., their HbA1c level did not drop below 7.5% at baseline) will be randomized in the study.
7) We will then randomize eligible consented participants to either the EPIC or EUC arm. Staff members will inform participants by phone to which arm they have been assigned. During this call, research staff will also verbally collect information on activation, health literacy and prior exposure to related diabetes treatment (e.g. diabetes education).

B.2.B. ii.. Patient-participant Compensation.

Participants will receive $25 after the completion of each assessment, for a total of $75 if the patient completes all assessments.

5.3 Informed Consent Procedures

To address the potential risks to participation and utilize data for the purpose of creating generalizable knowledge, we obtained informed consent for all participants in Phase 1. In Phase 2, we will consent both staff participants (group leaders and individual session providers) and patient-participants. Study participants will be recruited for the study in collaboration with VISN 12 and Houston-based and facility-level PACT leadership. We will use two recruitment approaches corresponding to our two subject populations.

5.3.A. Staff participants.

Prior to training, local research staff will consent all staff-participants (group leaders and individual session providers) using a written consent form for participation in Phase 2 All staff participants will be given an opportunity ask and have questions answered before agreeing to participate. The voluntary nature of the research will be clearly stated, including specific provisions that job status will be unaffected by the decision to participate. The confidential nature of the research will also be emphasized. Research data, including fidelity measures, will not be shared with supervisors or anyone outside the research team. All data generated by the research will be de-identified at publication.

5.3.B. Patient-Participants.

Patient-participants will be identified using the structured recruitment protocol **(§5.2.B.i.)**. Eligible patients who do not opt out of study participation will be contacted by study personnel to introduce the study objectives/procedures, and to obtain verbal consent to administer a screening protocol. Research staff conducting the telephone screening will give the patient an opportunity to ask and engage in a discussion on the merits of participation. If the initial screen indicates the patient may be interested and eligible for the study, the patient will be invited to attend a face-to-face introductory meeting, where a written consent to participate in the study will be offered. The patient will be encouraged to discuss participation with family and/or friends before the introductory meeting. The full consent process will be undertaken at the introductory meeting. Patients will be given another opportunity to ask and have

questions answered. Attendance at the introductory meeting will not require participation in the study. Patients will be free to leave the introductory meeting without enrolling in the research. Patients may also take the unsigned informed consent document home with them for further consideration (but should they return with a signed consent form desiring to participate, baseline data collection may be delayed depending on the availability of an EPIC group). After all questions have been addressed, patients will have the option to sign the informed consent document at the meeting.

Eligible patients will also be notified that they may be asked to sign form 10-3203 in the future to allow for voice recordings of a group session for the purpose of conducting fidelity assessment. Form 10-3203 will be presented to subjects at a later date when the need for a fidelity assessment is certain. Consent to voice recording will not be required to participate in the EPIC intervention. Should a subject not agree, the group session will not be recorded.

5.4 Inclusion/Exclusion Criteria

5.4.A. Staff Participants

Inclusion criteria: We will recruit VA staff who regularly provide diabetes care as group leaders and/or individual session providers.

Exclusion criteria: We will exclude staff who: 1) do not have a VA appointment, and 2) do not regularly provide diabetes-related care.

5.4.B. Patient Participants

Inclusion criteria: Using the Corporate Data Warehouse, we will identify diabetic VISN 12 and Houston-based patients meeting the study inclusion criteria: 1) ICD-9-CM codes indicating diabetes and 2) average HbA1c level ≥ 8% in the prior 6 months.

Exclusion criteria: We will use a medical record review to exclude potential participants with the following clinical conditions that would render participation in a group clinic inappropriate: 1) metastatic cancer or receiving hospice care, 2) limited life expectancy (as identified using a validated algorithm developed in our prior work [see Attachment 1]),[47] 3) clinician recommendations to not titrate therapy due to prior history of significant hypoglycemic events, 4) age <18 years, 5) active bipolar or psychotic disorder, 6) documented active substance abuse, or 7) documented dementia.

We will exclude participants at the time of screening who report to study staff that they 8) have substantial hearing or vision loss, such that participation with the materials and group exercises would not be possible,9) cannot attend bi-weekly group clinic sessions due to transportation or availability barriers, 10) have significant cognitive impairment (three or more errors on an established six-item screening exam), [63] 11) have active substance-abuse disorders, or 12) are not comfortable discussing their health and health care in a peer-group setting.

Patients will be secondarily excluded if their HbA1C level falls below 7.5% at baseline. Patients whose baseline levels fall below 7.5% may have limited ability for meaningful HbA1c change without significant concerns for hypoglycemia. We will notify all participants identified as having uncontrolled diabetes but who do not meet the final eligibility criteria (i.e., whose HbA1c drops below 7.5% at baseline) of their results. A note will be placed in their medical record indicating this finding and they will be withdrawn from the research.

5.5 Study Evaluations

5.5.A. Summative Evaluation of Implementation

We will conduct a summative evaluation of EPIC implementation after completing study Phase 2. We will characterize successful implementation along three (dependent) variables related to elements of the RE-AIM framework (reach, adoption, and implementation). The aims of Phase 2 will address the remaining two elements of RE-AIM (effectiveness and maintenance). We will evaluate RE-AIM along the measurement model described in Table 5 below.

5.5.A.i. RE-AIM Measures for the Summative Evaluation. We will assess reach by comparing the characteristics of enrolled study participants to those of all eligible patients participating. We will evaluate adoption among PACTs at the facility level by evaluating PACT team-level characteristics that differ among those with members who agree to participate versus others in a given facility. We will also calculate the total number of personalized goal-setting sessions that occur following a scheduled EPIC group session divided by the total number of EPIC group sessions patient-participants attended. Finally, we will collect descriptive information about adoption such as frequency and percentage of different types of professional disciplines of PACT members who participate in the personalized goal-setting. For implementation, we will evaluate the proportion of group sessions attended per patient, with the total possible number of group sessions (i.e., six) as the denominator and the proportion of individual sessions attended per

Table 5. RE-AIM Elements and Corresponding Measures

RE-AIM Elements (Phase 2)	Proposal's Corresponding Measures
**Reach:** Representativeness of patients who are willing to participate in the intervention	Characteristics of enrolled study participants from a given PACT patient panel compared to those of all PACT patients meeting eligibility criteria from that panel
**Effectiveness:** Intervention's impact on important outcomes, including negative effects like diabetes distress	Differences in HbA1c and DDS between EPIC and EUC study arms at 4 months (post-intervention)
**Adoption:** Representativeness of settings & intervention agents willing to initiate a program and their actual use of program or intervention components	1) Characteristics of PACT teams with participating members<br><br>2) Timing and frequency of one-on-one sessions following each group sessions
**Implementation:** The intervention agents' fidelity to the various elements of an intervention's protocol; patients' use of the intervention strategies; and the costs and cost-effectiveness of the intervention	1) Proportion of group sessions attended (out of six) for each enrolled patient<br><br>2) Proportion of individual sessions attended (out of six) for each enrolled patient<br><br>3) Objective ratings of individual session providers' fidelity to the collaborative goal-setting methodology using a structured fidelity rating process completed by a behavioral coaching expert on the study team<br><br>4) Patients' perceptions of goal-setting engagement by providers in both the intervention and EUC arms<br><br>5) Objective ratings of goal and action plan quality using our validated GET-D tool by trained research staff blinded to random assignment<br><br>6) Cost-utilization and cost-effectiveness of EPIC compared with EUC arms.
**Maintenance:** Long-term effects of a program on outcomes 6 or more months after the most recent intervention contact	Differences in HbA1c and DDS between EPIC and EUC study arms to measure intervention persistence at 10 months

patient (i.e., 0-6) as the numerator. We will also examine fidelity ratings of all VA staff

trained to lead the EPIC group sessions and those trained as individual session providers, who conduct personalized, collaborative goal-setting; these ratings will be performed after training by a behavioral coaching expert on the study team. The study team will then measure patient-participants' self-reported ratings of how much their group leader and individual session provider(s) engaged them in goal-setting using a validated measure, [61; 62] and objective ratings of goal and action plan quality using our previously validated rating GET-D tool.[48] We will assess cost-effectiveness of this study from a perspective of the VA health care system using a comprehensive cost-based database system. We will use a micro-costing approach to track and record all expenses related to the EPIC and EUC components and non-research related resource consumptions such as the educational materials and staff time spent on both study arms. We will retrieve medical utilization and cost data from the National Patient Care Database and the Decision Support System. We have experience working with each of these data sources in our prior HSR&D funded work. The National Patient Care Database includes outpatient and inpatient clinical, demographic, and utilization data (e.g., patient age, race, diagnosis and procedure codes, clinic location where care is provided, and the provider of care). The Decision Support System, a managerial cost accounting system, produces National Data Extracts that provide cost and utilization information for a range of health care activities, including laboratory, pharmacy, radiology, outpatient services, and inpatient treating specialty units. Unit cost of personnel time will be based upon the actual salary rate and fringe. Unit cost of other resources such as supplies and facilities will be derived from the VA accounting system. Total costs for each patient will be the summed products of quantities of resources used multiplied by the unit cost for those resources. All costs will be adjusted to constant US dollars in 2016. For cost-effectiveness, we will use two measures: 1) number of study patients with clinically significant improvements in HbA1c; and 2) number of quality adjusted life-years using the validated EQ-5D instrument to derive health utility weights.[63] The utility score (weight) of each individual patient at each observational interval over the trial period (baseline to 4 months to 10 months) will be calculated according to the scoring algorithms provided by the EQ-5D developers.[64] The primary end-point measures of cost-effectiveness are: 1) the incremental cost per additional number of study patients whose HbA1C are significantly improved and 2) the incremental cost per additional quality adjusted life-year gained, of the intervention relative to the control group.

5.5.B. Data Collection Strategy § 5.1.H.

5.5.C. Study Variables § 5.1.I.

5.6 Data Analysis

5.6.A. Sample Size Calculation/Sample size determination

Sample size is calculated according to the estimated intervention effect size at post-intervention. We then estimate power to detect treatment effects at the post-maintenance (10-month) follow-up as well as power to detect treatment differences in linear change across the three time points for a 3-level cluster-randomized trial with repeated assessments. We will adjust models for baseline covariates of study patients. All tests will be two-sided with an alpha of 0.05. In our recent RCT, differences in HbA1c change between EPIC versus enhanced group education indicated medium treatment effects at post-treatment and at 1-year (Cohen's d = 0.48 and 0.42, respectively). A similar trial [65] revealed a treatment difference between a glucose self-monitoring protocol and an active control group in DDS scores that correspond to large effects of treatment at 1–year (all pre-post ds > 0.80). To capture treatment effects for both clinical and patient centered outcomes in this implementation trial, a conservative small-to-medium effect size of d = 0.40 (which is 16.67% smaller than the effect found for

HbA1c in the prior trial) was used to calculate sample size. Assuming no intra-class correlation (ICC) within PACTs, 100 patients in each treatment arm (i.e., EPIC and EUC) will allow for 80% power to detect small-to-medium effects and 98% power to detect medium effects ($d = 0.50$). To account for the dependency among patients within a PACT, the Design Effect (Deff) was applied, following the approach of Schnurr et al.[66] The sample size was inflated using the formula, $Deff = 1 + (n-1)\rho$, where n is the average number of patients per PACT and ρ is the ICC for PACTs. In our preliminary work, we identified an ICC for PACTs of 0.0183, an average of 27 eligible patients per PACT, and expect that an average of 12 patients per PACT will participate, which yields $Deff = 1 + (12-1) * 0.0183 = 1.2013$. Applying this adjustment, the minimum number of patients in the clustered design is 100 x 1.2013 = 120 in each treatment group. Further adjusting for a maximum of 15% attrition, 142 patients will be recruited for each treatment group (total N = 284). Therefore, the minimum number of PACTs to be sampled for this nested analysis is technically 11.8, (i.e., 142/12), which will be rounded up to 24 PACTS. This is highly feasible, representing just 32.5% of the total number of PACTs (75) at all study sites. Treatment group effect sizes as small as $d = .40$ can be detected with 80% power at 1-year given a total of 284 patients (an average of approximately 12 patients randomized to either EPIC or EUC from within 24 PACTs sampled), even after accounting for maximum attrition and estimated dependency within PACTs. A sample size of 284 participants is adequate for repeated measures analyses as well. Optimal design software estimated power to detect treatment group differences in linear change across all three assessments.[67] Prior data indicated a main effect of treatment (EPIC versus enhanced group education) for linear change in HbA1c of 0.20, and between – and within – PACT variance in linear change of 0.018 and 0.206, respectively. These values indicate a small-to-medium between-groups effect size of 0.42. A total of 284 participants allows for 80% power to detect a slightly larger effect size ($\delta = .53$) for repeated measures analyses of linear change over time. Furthermore, there is 98% power to detect a medium effect size of $\delta = .75$.

5.6.B. Data Collection Strategy § 5.1.H.

5.6.C. Data Analysis

Specific Aim 1: H1 Analysis (Summative Evaluation). We will first calculate descriptive statistics such as frequencies, proportions, means, and standard deviations for reach, adoption, and implementation measures for the overall sample (i.e., VISN 12 and Houston) and for each specific facility. We will determine cost-utilization of resources within both study arms and the incremental cost-effectiveness ratio (ICER), which is the difference in the estimated mean cost between the intervention and control groups divided by the difference in the estimated mean effectiveness between the two study arms. The base-case will be the control group. We will estimate two ICERs: 1) the incremental cost per additional number of study patients with clinically significant HbA1C reductions, and 2) the incremental cost per additional quality adjusted life-year gained, of the intervention arm over the study period respectively. We will calculate ICER as a ratio of the difference in the estimated mean total cost between the EPIC and EUC groups divided by the difference in the estimated mean number of patients whose HbA1c levels are significantly improved between the two study arms. Similarly, we will calculate ICER of the intervention in terms of the quality adjusted life-year as a ratio of the difference in the estimated mean total cost between the intervention and control groups divided by the difference in the estimated mean total number of quality adjusted life-year between the two study arms. We will use a commonly used threshold, $50,000 per quality adjusted life-year gained, as a reference point to determine if the intervention is cost effective.

Because cost data are typically right-skewed and also subject to bias due to death and/or attrition, we will directly model the logarithm of costs using generalized linear modeling with a logarithmic link function and inverse probability weight to adjust for these potential biases. We will control any baseline imbalance between groups with respect to the cluster and study population characteristics in the calculations of expected mean cost and effectiveness. The estimated value of

cost and quality adjusted life-year will not be discounted given a relatively short follow-up period in the study.

We will conduct exploratory analyses to examine associations between implementation measures (RE-AIM elements in table 5) and study outcomes following the conclusion of Phase 2. For example, for all eligible patients, within each PACT demographic characteristics will be compared between enrolled and non-enrolled patients using chi-square tests and independent samples t-tests. Fisher's Exact Test and the Wilcoxon Mann-Whitney tests will be used where appropriate. An index of reach representativeness will be calculated for each PACT which will then be correlated with post-intervention outcomes, controlling for respective baseline values. Similarly, for all PACTs sampled, PACT characteristics (e.g., panel size) will be compared between sampled and non-sampled PACTs using chi-square tests and independent samples t-tests. An index of adoption representativeness will be calculated for each PACT which will then be correlated with post-intervention outcomes, controlling for respective baseline values. Additionally, for patients receiving the EPIC intervention within each PACT, post-intervention HbA1c will be separately regressed on 1) the proportion of group sessions attended, 2) the proportion of individual sessions attended, and 3) baseline objective ratings of the group leader's fidelity. These models will control for baseline HbA1c and will be conducted using ANCOVA methods. Predictors that are significant at $p < 0.25$ will be included in a multiple linear regression to examine both collective and unique predictors of post-intervention HbA1c levels (once again controlling for baseline HbA1c). Similar univariate and multivariate models will be formed to predict post-intervention DDS.

Specific Aim 2: The distributional nature of all variables will be assessed, and nonparametric tests (e.g., Fisher's Exact Test; Mann-Whitney test), data transformations (e.g., log linear), or other alternate methods (e.g., weighted least squares regressions) will be conducted where appropriate. First, we will compare baseline demographic, clinical, and patient-centered variables (including medication use) between EPIC and EUC with chi-square and independent samples t-tests. Variables with p-values < 0.25 will be included as control variables or propensity scores in subsequent models[101.] We will then compare baseline demographic, clinical, and patient-centered variables between those who complete the study and those who do not using chi-square and independent samples t-tests. Outcome analyses at both post-intervention and post-maintenance will be intention-to-treat and will use the multiple imputation procedures Proc MI and MINANALYZE in SAS Version 9.3 to estimate missing observations[101.] We will evaluate the degree of dependency between patients within a given group session, between patients in a given PACT, and between PACTs within each of the five sites (by examining Intra Class Coefficients). It is likely that significant dependency will exist, and if so, we will accordingly take these into account in analyses (i.e., patients will be nested within PACTs which will in turn be nested within sites). Random regression methods using SAS Proc Mixed will be employed to account for clustering of data.

<u>5.6.C.i. H2 Analyses (Effectiveness)</u> We will employ Analysis of Covariance (ANCOVA) to examine treatment differences in outcomes immediately post-intervention (at 4 months). We will conduct two models: one with HbA1c at post-intervention as the outcome and one with DDS at post-intervention as the outcome. Models will include treatment group (i.e., EPIC versus EUC) as a predictor and respective HbA1c and DDS baseline scores and any demographic, clinical, or patient-centered variables that differed between the study arms at baseline as covariates. We will calculate treatment effect sizes immediately post-intervention.

<u>5.6.C.ii. H3 Analyses (Maintenance)</u> Analyses for examination of maintenance of treatment effects will be similar to those for immediate treatment effects post-intervention. We will again employ ANCOVA to examine treatment differences in outcomes at the post-maintenance (10-month) assessment. We will conduct two models: one with HbA1c at 10-months as the outcome and one with DDS at 10-months as the outcome. Models will include treatment group (EPIC versus EUC) as a predictor and respective HbA1c and DDS post-intervention scores and any demographic, clinical, or

patient-centered variables that differed between the study arms at baseline as covariates. We will calculate treatment effect sizes at the 10-month assessment.

5.6.C.iii. Exploratory Analyses (Implementation and Effectiveness) We will use a mixed-model approach to conduct separate repeated-measures analyses for HbA1c and DDS simultaneously using all three assessment time points. We will employ growth curve analyses using SAS Proc Mixed to examine overall group differences in improvements or decrements in outcomes over the year, maximize participant data, and account for dependency between patients within a given group session, PACT, and site. Conditional models will contain fixed terms for the intercept, treatment (EPIC or EUC), assessment time period, treatment by time period interaction, and previously identified variables that differ between treatment groups. Modeled random effects will include between-patient variation in baseline scores (i.e., the intercept where baseline assessments are scored 0) and variation in the slopes for time. With three assessments, the focus will initially be on linear patterns of change, although we will evaluate the relative fit of a quadratic pattern of change using the likelihood ratio test. These analyses will allow us to examine the immediate impact of treatment at post-intervention as well as retention, improvement, or decay in outcomes post-maintenance period. The treatment effect will assess differences between the two groups at baseline, the fixed effect of time will measure the average change over time in the outcome (collapsing across the two treatment groups), and the time by treatment interaction will indicate whether change over time (in slopes) differs between EPIC and EUC.

Several variables will be examined as separate mediators of the relationship between intervention group (EPIC versus EUC) and post-intervention outcome variables: 1) patients' perceptions of goal-setting engagement by the designated PACT member (CSQ), 2) objective ratings of goal and action plan quality (GET-D), 3) self-efficacy for diabetes self-management, 4) diabetes self-management adherence, and 5) mediation adherence. For each mediator, we will conduct three separate models to test for mediation between intervention group and each outcome: 1) the first model will regress post-intervention HbA1c levels on treatment group and baseline HbA1c levels, 2) the second model will regress the mediator on treatment group and baseline HbA1c levels, 3) the third model will regress post-interventionHbA1c levels on treatment group, baseline HbA1c levels, and the mediator. Parallel analyses will be conducted to predict change in DDS. We will use bootstrapping methods to calculate the unstandardized estimate of the indirect effects as well as unbiased confidence intervals.[99] Significance will be established if the 95% confidence interval of the indirect effect does not include zero. Bootstrapped analyses will be performed using MPlus Version 6. [100]

5.6.D Data Analysis Logistics

Phase 2: VA administrative data will be accessed and stored on the VA's centralized and secure Information and Computing Infrastructure (VINCI). VINCI is a major informatics initiative of the Department of Veterans Affairs (VA) that provides a secure, central analytic platform for performing research and supporting clinical operations activities. It is a partnership between the VA Office of Information Technology (OI&T) and the Veterans Health Administration Office of Research and Development (VHA ORD). VINCI includes a cluster of servers for securely hosting suites of databases integrated from select national VA data sources. VINCI servers for data, applications, and virtual sessions are physically located at the VA Austin Information Technology Center (AITC), located in Austin, Texas. This secure data storage enclave has multiple layers of security and disaster recovery to prevent data loss. To ensure the protection of Veteran data, VINCI maintains compliance with the guidelines set forth by Veterans Health Administration (VHA) Handbook 1200.12. Accesses to VINCI resources are approved in accordance with the requirements of National Data Systems (NDS), "VHA Handbook 1200.12, Use of Data and Data Repositories in VHA Research", and all other applicable VA and VHA policies and regulations. Study data stored on VINCI servers are located at the Austin Information Technology Center, 1615 Woodward St., Austin, TX 78772-0001.

Data necessary for recruitment will be imported into a study database stored on the local drive. Recent experience has shown that, at the moment on the VINCI platform, access and computing is very slow compared to the local servers. Storing the database locally will provide broader, faster access to research staff who are delegated to use the database. Accordingly, we will house the MS Access database for recruitment and data collection on the local server. As MS Access is not feasible to use on VINCI, research staff will prepare a limited data set which meets HIPAA standards which can then be downloaded via secure FTP from VINCI to a local VA secure server located at the Houston VA HSR&D IQuEST. This limited data set will then be imported to the MS Access database stored on the Houston VA HSR&D IQuEST secure server. Preparation of the limited data set to be downloaded to the Houston VA IQuEST shared drive will occur only within the VINCI secure platform. Copies of other data sources will be uploaded to the Project folder within VINCI from the location of current storage after appropriate approvals with the data custodians are established.

Data analyses will take place with a number of statistical programs including SAS, and potentially Microsoft SQL Server (T-SQL), Stata, and/or R. All these resources are available to research staff on the VINCI secure computing platform, reducing the need for large data transfers to local VA secure servers. However, one resource that is lacking at the moment on the VINCI platform is the software which will be used to statistically analyze the constructed cohort files. Mplus is a versatile and commonly used structural equation modeling software application which has been approved and tested by VA OI&T for use within the VA. This software will be used to complete the final inferential statistical analyses in this protocol. Current software applications on the VINCI system (e.g., SAS, Stata, R) do not yet contain procedures/packages which can accommodate the inferential statistical analyses outlined in this protocol. As Mplus is not available on VINCI yet, research staff will prepare a limited data set which meets HIPAA standards which can then be downloaded via secure FTP from VINCI to a local VA secure server located at the Houston VA HSR&D IQuEST. This limited data set will then be analyzed from the Houston VA HSR&D IQuEST secure server.
Preparation of the limited data set to be downloaded to the Houston VA IQuEST shared drive will occur only within the VINCI secure platform. Analyses with VA data that do not involve the structural equation models described in this protocol will be completed in the VINCI workspace and secure computing resources provided by VINCI staff (e.g., SAS, MS SQL Server, Stata, or R). However, once the cohort files have been constructed and are suitable for structural equation modeling, preparation of the limited data set will then involve removing all patient identifiers. For this protocol, patient identifiers include the VA's scrambled SSN (SCRSSN), real SSN, dates, and zip codes. The limited data set will then be stripped of these patient identifiers in the following process before transfer from the VA's VINCI platform to the Houston IQuEST local secure server:

1) Real SSN will be completely deleted immediately from the limited data set.
2) PatientSID and PatientICN variables from CDW will be completely deleted from the limited dataset,
3) SCRSSN will first be sorted randomly in the dataset and then encoded to anonymous numbering (i.e., 1, 2, 3, 4…N) unique to this limited data set. This procedure anonymizes the records with respect to individual VA patient identification, but preserves the essential nesting structure of multiple non-independent records nested within participant in the limited dataset. SCRSSN will then be completely removed from the limited date set prior to download from VINCI FTP to the local Houston VA IQuEST secure server.
4) Similar to SCRSSN, dates will be encoded such that the same dates in the limited data set retain the same ordering, but values will not be identified as dates. For example, SEP272013 might be codes as 74 with SEP282013 coded as a 75, and so on. This approach preserves the order and parametric qualities of former date variables, but does not allow any identification of actual dates of care in the limited data set. Actual date values in the entire limited data set will then be deleted prior to download from VINCI FTP to the local Houston VA IQuEST secure server.

5) As with the process of anonymizing SCRSSN and deleting this variable, zip codes will be encoded such that the zip code variable will first be sorted randomly and then encoded to non-identifying numbers unique to this limited dataset. For example, zip code 55555 might be codes as 1, 72468 might be coded as 2, 56912 might be coded as 3, etcetera. This step will again be completed in VINCI prior to secure FTP download to the Houston VA IQuEST's secure server. After encoding zip code, zip code will be deleted from the limited dataset.
6) Final checks that all identifying information has been removed from the dataset will be made, and
7) The limited data set will be transferred from VINCI to Houston VA's IQuEST secure server for analysis with VA approved Mplus software (once again, as this software is not available on the VINCI platform, but approved by VA OI&T).
8) No means of linking VA data stored in the VINCI project workspace with values in the limited data set will be available outside of VINCI.

It is important to note that VINCI has an audit function built in such that review of FTP downloaded data does not violate HIPAA or VA policies. The 8 step approach outlined above, along with this audit/data download monitoring function that VINCI maintains will ensure that PII/PHI remain securely protected and confidential.

The primary person(s) processing and analyzing data will be the Houston Data Analyst(s). The Houston-based investigators (Woodard, Naik, Amspoker, Arney, and Hundt) will assist with data analysis when needed. Dr. Woodard will have primary responsibility for oversight of all data analysis work.

5.7 Withdrawal of Subjects

5.7.A. Group Leaders and Individual Session Providers.

5.7.A.i. Investigator termination of subject participation: The investigator does not anticipate any circumstances under which subjects will be withdrawn from the research without their consent.

5.7.A.ii. Consequences of withdrawal: If a participant decides to withdraw, there are no foreseeable consequences. A replacement will need to be identified, consented and trained to complete study enrollment.

5.7.A.iii. Procedure for orderly termination of participation by the subject: The subject must notify the investigator, or Research Coordinator, by telephone or written correspondence of their desire to withdraw from the study. When possible, the subject will finish working with the current cohort of subjects before terminating participation.

5.7.B. Patient Participants

5.7.B.i. Investigator termination of subject participation: The investigator does not anticipate any circumstances under which subjects will be withdrawn from the research without their consent unless the participant develops a condition on the exclusion criteria that will put them at risk.

5.7.B.ii. Consequences of withdrawal: If a participant decides to withdraw prior to the completion of the baseline assessment, the only consequence to the subject would be not receiving study compensation (because they would not have completed baseline assessment as required). If a participant decides to withdraw at any point after baseline, there are no foreseeable consequences.

5.7.B.iii. Procedure for orderly termination of participation by the subject: The subject must notify the investigator, or Research Coordinator, by telephone or written correspondence of their desire to withdraw from the study.

6.0 Reporting

All unanticipated serious adverse events (U-SAEs) and unanticipated serious problems (UAPs) will be reported to the VA Central IRB within five business days. U-SAEs will be reported to VA Central IRB regardless of their relationship to the research. All protocol deviations, violations, and/or noncompliance will be reported to the VA Central IRB within five business days of the reporting individual becoming aware of the occurrence.

Safety information, including SAEs,that will be collected:

Occurrences of events resulting in a participants' death, life threatening experience, hospitalization, prolonged hospitalization, or persistent or significant disability related to hypoglycemia will be defined as a Serious Adverse Event and documented. Any occurrence of an event that results in the need for medical or other interventions to prevent any of the above listed outcomes will be documented as well. As such, any participants identified as having an immediate physical health issue will be referred to care as appropriate.

Frequency/methods of safety-related data collection:

Collection of safety information will commence when the first participant is enrolled in the study; this is anticipated to occur during Spring 2015. Safety information may be collected either 1) during baseline and follow up assessments, 2) during EPIC sessions, or 3) during telephone contacts with participants made for purposes of scheduling assessments and/or treatment sessions. Also, the Research Coordinator or RA will periodically contact patients to schedule study-related safety appointments. The participants or other informants may report information related to their safety at those times.

Conditions that would trigger an immediate suspension of the research:

This intervention will compare a brief, structured goal-setting intervention with usual care practices in VA facilities. The active treatment (EPIC) utilizes an empirically-supported theory to enhance patients' self-management of diabetes. No invasive procedures or untested techniques will be used. As such, this protocol is judged to be of low risk. We do not anticipate the occurrence of events that would necessitate the immediate suspension of research because of 1) the low probability of adverse events from the intervention in either arm of the study, 2) all participants will continue to receive usual care services within the VA, and 3) treatment for any VA services will not be withheld from any participants.

Specify procedures to determine when and how to notify individual participants or their health care providers of findings that may affect the participant's health or welfare:

The decision to contact a patient and/or their health care provider regarding patient welfare can be made in two ways. First, the Project Coordinator or research staff will conduct routine checks on participants' safety and well-being during baseline and follow up assessments. The study personnel will notify the patient and/or their healthcare provider as necessary.

Second, data and safety monitoring is expected to be conducted at both the local and national levels. At the local level, the study PI (Woodard), site PIs (Damstra, Hertz, Ryan), co-investigators (Naik, Amspoker, Hundt, Arney) will work with the study programmer and statistician to review data and safety issues regularly during monthly investigator meetings or more immediately as needed. Data and safety monitoring will occur for any identified adverse events as well as including a regular monitoring schedule of participant longitudinal data. Any participants identified as having an immediate physical health issue will be referred to care as appropriate. All participants, regardless of treatment, with a 20% increase in symptoms (relative to baseline) will be called to ensure safety and encourage the participant to obtain care if desired.

At the national level, we anticipate participating in the VA's Data and Safety Monitoring Board (DSMB). We will provide the national DSMB with comprehensive annual and semi-annual reports, as directed, for formal independent review of study safety and recruitment practices.

7.0 Privacy and Confidentiality

7.0.A. Privacy and Confidentiality

To minimize the risk of unintentional disclosure of personal information, all electronic and paper data collected for this study will be kept in secure storage. Access to data with individual identifiers will be restricted. Data for all participants will be identified by study ID number only. Links between the study ID and personal identifying information will be maintained separately. Neither the participant's name nor any other identifying information will be connected to any information they provide. Extensive measures are taken to maximize privacy and confidentiality of data, as described next. A Certificate of Confidentiality will not be obtained.

7.0.B. Data security protocols for Houston VA HSR&D IQuEST Computing Center users

All project staff is required to have undergone significant training on the protection of human subjects, research methods and the importance of integrity in the research process. Houston VA HSR&D IQuEST Computing Center also requires all project staff to review the Data Security Compliance Agreement which describes the center's data security protocol. Each project staff member must sign an acknowledgement that they have reviewed the policy and agree to follow the policy before accessing data. The Houston VA HSR&D IQuEST Computing Center data security policy conforms to current VA policies and has been reviewed and approved by the MEDVAMC Chief Information Officer, Information Security Officer, and Privacy Officer.

No individual-specific data from the secondary data analyses will be released to anyone except the VA research team members with data access privileges (**§5.6.**). All findings will be presented as aggregated results. No individual-specific data from the qualitative data interviews or data analysis will be released to anyone except the approved qualitative interview study team members**.** All findings will be presented as aggregated results.

The main risk of this project is unauthorized access to the patient data. We have a multi-layered system in place to prevent unauthorized access to the data.

1. The computer system at the Houston VA HSR&D IQuEST is behind the VA firewall. The system servers are behind a locked door with access limited to IT personnel. During non-business hours, the servers are behind 3 locked doors. IQuEST has restricted physical access and is not a patient-care facility. The servers are backed up automatically each night.
2. The physical address of the servers is Houston VA Medical Center, HSR&D Center for Innovation in Quality, Effectiveness and Safety, 2450 Holcombe Blvd, Suite 01Y, Houston, TX 77021, Room 166.
3. The computer server that this project will use for data analysis is configured to limit access. Users must be logged on to the VA internal network to access the server.
4. All HSR&D IQuEST research projects that use confidential data have project-specific directories configured so that project staffs are the only system users that can access the directory.
5. The data files for this project will be encrypted and will reside in password-protected electronic folders that will be maintained by the HSR&D IQuEST Computing Center in accordance with all VA data security measures.
6. VA HSR&D IQuEST issues login accounts only to VA research staff who can demonstrate need to use the secure server. The Principal Investigator must sign a Delegation of Authority form for each study team member who is requesting access to the secure project directory. The Delegation of Authority form must be approved by the HSR&D IQuEST Research Assurance and Data Security (RADS) Coordinator, who will in turn submit a request to the center's IT Manager to add the individual study team member to the approved access list for the project's electronic directory.
7. Within 24 hours of an individual leaving the study team, the PI or the Research Coordinator will submit a request to the IT Manager (with a copy to the RADS Coordinator) to remove the individual from electronic access to the project directory on the VA server.
8. A "shared drive" will be established on the Houston VA HSR&D IQuEST secure server behind the national VA firewall for the purpose of providing access to approved study team members or investigators at other VA locations. Those individuals must be logged in to the VA internal network to access the server.

7.0.C. Data security during transfer of data between VA facilities (data with Real SSNs and Scrambled SSNs as identifiers)

This study, which involves analyses of databases, requires data transfers between the Houston VA HSR&D IQuEST and other VA facilities which are pulling data for us on subjects in the VA cohort finder file that we send to them (e.g., for CDW data from VINCI).

Scrambled SSN will be used as the patient identifier for linkage with VA databases residing at other VA facilities wherever possible; however, real SSN/names will be required for some finder files. Any needed transfers of data between VA facilities will occur via one of the following VA-approved mechanisms for secure transfer (in password-protected files encrypted with VA-approved standard of encryption):
1. Direct file transfer over VA server behind the national VA firewall.
2. Direct file transfer using SFTP (secure FTP) to move file from server at one VA to server at another VA (this will require a VA data analyst at the recipient VA to remotely log onto the Houston VA HSR&D secure server to download the data to his/her VA server, and vice versa).

We will work with our Houston VA HSR&D IQuEST IT Manager and with the VA entity that serves as the data owner (e.g., VINCI) to assure that our data transmission approach meets the most up-to-date national and local VA standards.

No data access will be provided to anyone outside the study team, except that a finder file of either scrambled SSNs or Real SSNs of patients in our cohort (along with any other data elements necessary for matching, including sex and date of birth) will be sent to the centralized VA repositories (e.g., VINCI) so that they can pull necessary data elements for us. Only study staff that needs access to the data to perform their research functions will have access to PHI. Paper data containing baseline patient-level data will be stored securely within the office space of the local site investigator behind 2 locked doors. Any temporary print-out copies of record-level data elements printed to facilitate inspection of the data **will not** contain scrambled SSNs, Real SSNs, or provider identification numbers. Any data printouts will be stored in a locked cabinet in a locked research room when not in use, and will be securely shredded as soon as inspection is complete. Individual-level PHI will never be reported in any presentation of the data; data will only be presented in aggregate. The data will be kept on secure, password-protected VA servers.

7.0.D. Data destruction

We will maintain the data files and all datasets created from the data files on the local, secure server at least as long as data analysis is ongoing, and for the period of time as required in the Record Control Schedule (RCS) for VA research records per the VA directives regarding retention of study data. At this time, VA research records do not have RCS – therefore all VA research records will be stored until disposition instructions are approved by the National Archives and Records Administration are published in VHA's Records Control Schedule (RCS 10- -1). When it is time to destroy the data, we will follow data disposition instructions approved by the RCS.

8.0 Communication Plan

8.0.A. Plan for ensuring all required local site approvals are obtained and notifying the Director of any facility where the research is being conducted but the facility is not engaged.

8.0.A.i. Plan for engaged facilities:

Upon approval of the PI/SC application Form 108, each local site will submit VA Central IRB Form 104 (Local Site Investigator Application), which must be signed by the Local Site Investigator, his/her supervisor, and the local site ACOS/R&D or Chief of Staff.

Upon VA Central IRB approval of the Form 104 Local Site Investigator Application, the local site R&D Committee must provide written approval for the research to be conducted at the local site before the research begins.

The Research Coordinator will maintain copies of the local site R&D Committee approvals in the main site regulatory binder.

Local site Investigators or their designated study team member Research Assistants (RAs) will maintain copies of the main site approval, as well as the local site R&D Committee approvals in their respective local site regulatory binders.

8.0.A.ii. Plan for non-engaged facilities:

Upon VA Central IRB approval of the PI/SC New Project Application, the Principal Investigator will notify the VISN 12 sites, to submit a request for approval to conduct research on this study to the local VA Facility Director and to the local site Research & Development Committee.

This research study will not take place at any other facility not engaged in the research (i.e., without a Local Site Investigator Project Application approval).

8.0.A.iii. Plan for notifying and obtaining local site approval of amendments and other administrative changes:

Upon VA Central IRB approval of all PI/SC Amendments and Local Site Amendments (including modifications to the protocol, the informed consent form, and the HIPAA authorization), the Research

Coordinator will send an electronic copy of the approval and all attachments via email to the Local Site Investigator to submit to the local site R&D Committee for approval.
The Research Coordinator will maintain copies of all local site R&D Committee approvals in the main site study binder
The local site Investigator or local site RA will maintain copies of their respective local site R&D Committee approvals in their local site study binder.

8.0.B. Plan for keeping all engaged sites informed of changes to the protocol, informed consent, and HIPAA authorization

See **8.0.A.iii**

8.0.B.i. Regular meetings and conference calls The PI will lead regular conference calls and meetings that will include discussions of changes to the protocol, informed consent process and the HIPAA authorization. Study team members will be notified through these conference calls and meetings of upcoming changes, as well as when the PI receives notification from the VA Central IRB of final approval of such changes. The PI will lead weekly meetings to discuss the study status with the study leadership team (select co-Investigators, Research Coordinator, Data Analysts, Biostatistician, and other study team members). Initial weekly meetings will be devoted to training local site investigators and staff on informed consent procedures. The PI will also lead quarterly conference calls to host status update/discussions with all co-Investigators, Local Site Investigators, and all local site study team members.

8.0.B.ii. Shared drive The Research Coordinator will maintain a shared drive on the Houston VA HSR&D IQuEST secure server (that resides behind the VA firewall) that is accessible to local site study team members **(see §7.0.B.).** The Research Coordinator will maintain the most current version of all IRB approved documents on this shared drive.
When new or revised documents are submitted for approval, the Research Coordinator will notify the Local Site Investigator and his/her study team that changes have been submitted for approval and are under review by the VA Central IRB.
Upon VA Central IRB approval of a new or revised form, the Research Coordinator will notify by telephone and by email each Local Site Investigator and his/her study team that the new form has been approved.
All local site personnel will be asked to do the following:

- File a printed copy of the VA Central IRB approval, and all newly approved documents, in the local site study binder.
- Destroy all copies of previously approved versions of ICF, HIPAA, or other study forms.
- Begin using the new form, or applying the newly approved procedure, immediately.

The PI or the Research Coordinator will provide training on newly approved procedures to all local site study team members.

8.0.C. Plan for informing local sites of any Serious Adverse Events (SAEs), Unanticipated Problems, Protocol Deviations, or interim results that may impact conduct of the study

The Research Coordinator will notify all participating sites immediately of any SAEs, Unanticipated problems, or interim results that have the potential to affect implementation of the study. A copy of the SAE report or Protocol Deviation report that is submitted to the VA Central IRB will be sent to the Local Site Investigator, as well as their local site study team members via encrypted email. Additional copies will be sent to the local site R&D Committees.

- The PI will discuss SAEs, Unanticipated Problems, Protocol Deviations, and interim results that may affect the conduct of the study on the regular conference calls.

8.0.D. Plan for ensuring the study is conducted according to the IRB-approved protocol.

- The importance of conducting the study according to the IRB-approved protocol is emphasized by the PI to all study team members on a regular basis. In particular, all research team members are required to read the IRB-approved protocol (and any subsequent amendments), and research staff will receive specific training from the PI or Research Coordinator regarding protocol elements relevant to their study role before their involvement in the study begins. This study-specific training is over and above the mandatory trainings that all research staff receives.
- During weekly and monthly conference calls, the PI will follow-up with the LSIs to ensure that they continue to adhere to the protocol and to standard research compliance procedures as required by the VA.
- The PI will require the LSIs to hold weekly or bi-weekly meetings with their respective local site study teams

8.0.E. Plan for notifying all local facility directors and LSIs when a multi-site study reaches the point that it no longer requires engagement of the local facility (e.g., all subsequent follow-up of subjects will be performed by the PI from another facility).

- The PI will notify the LSIs when the study reaches the point at which it no longer requires engagement of the local facility.
- The LSIs will notify their respective local site Facility Directors and R&D Committees that their facilities will no longer be engaged in the research.

9.0 References

1. VA DM-QUERI. Diabetes Mellitus Fact Sheet. The DM-QUERI Executive Committee, editor. 2011. VA QUERI. 10-28-0011.
2. Snow V, Weiss KB, Mottur-Pilson C, for the Clinical Efficacy Assessment Subcommittee of the American College of Physicians*. The Evidence Base for Tight Blood Pressure Control in the Management of Type 2 Diabetes Mellitus. Annals of Internal Medicine. 2003;138:587-592.
3. Norris SL, Lau J, Smith SJ, Schmid CH, Engelgau MM. Self-management education for adults with type 2 diabetes: a meta-analysis of the effect on glycemic control. Diabetes Care. 2002;25:1159-1171.
4. Bodenheimer T, Wagner EH, Grumbach K. Improving primary care for patients with chronic illness: the chronic care model, Part 2. JAMA. 2002;288:1909-1914.
5. Funnell MM, Anderson RM. Working toward the next generation of diabetes self-management education. Am J Prev Med. 2002;22:3-5.
6. Naik AD, Palmer N, Petersen NJ, Street RJ, Suarez-Almazor M, Haidet P. Comparative effectiveness of goal setting in diabetes mellitus group clinics: randomized clinical trial. Arch Intern Med. 2011;171:453-459.
7. Locke EA, Latham GP. Building a practically useful theory of goal setting and task motivation. A 35-year odyssey. Am Psychol. 2002;57:705-717.
8. Strecher VJ, Seijts GH, Kok GJ, Glasgow RE. Goal Setting as a Strategy for Health Behavior Change. Health Education & Behavior. 1995;22:190-200.
9. Bodenheimer T, Handley MA. Goal-setting for behavior change in primary care: an exploration and status report. Patient Educ Couns. 2009;76:174-180.

10. Naik AD, Teal CR, Rodriguez E, Haidet P. Knowing the ABCs: A comparative effectiveness study of two methods of diabetes education. Patient Educ Couns. 2011; 85(3):383-9.
11. Gonzales R, Handley MA. Improving Glycemic Control When "Usual" Diabetes Care Is Not Enough: Comment on "The Effect of a Structured Behavioral Intervention on Poorly Controlled Diabetes" [published online ahead of print October 10 2011]. Arch Intern Med. 2011. http://archinte.ama-assn.org/cgi/content/full/archinternmed.2011.496v1. Accessed November 9, 2011.
12. Hibbard JH, Greene J, Tusler M. Improving the outcomes of disease management by tailoring care to the patient's level of activation. Am J Manag Care. 2009;15:353-360.
13. Sudore RL, Landefeld CS, Pqrez-Stable EJ, Bibbins-Domingo K, Williams BA, Schillinger D. Unraveling the relationship between literacy, language proficiency, and patient-physician communication. Patient Education and Counseling. 2009;75:398-402.
14. Woodard LD, Landrum CR, Wang D, Petersen LA, Naik AD. Association between health literacy, patient activation, and glycemic control among diabetic Veterans with multimorbidity. In press, J Gen Intern Med.
15. Skyler JS, Bergenstal R, Bonow RO. Intensive Glycemic Control and the Prevention of Cardiovascular Events: Implications of the ACCORD, ADVANCE, and VA Diabetes Trials. Circulation. 2009;119:351-357.
16. Anderson RM, Funnell MM. Patient empowerment: reflections on the challenge of fostering the adoption of a new paradigm. Patient Educ Couns. 2005;57:153-157.
17. Funnell MM, Brown TL, Childs BP, et al. National Standards for Diabetes Self-Management Education. Diabetes Care. 2009;32:S87-S94.
18. Pogach LM, Brietzke SA, Cowan CL, Jr.. Development of evidence-based clinical practice guidelines for diabetes: the Department of Veterans Affairs/Department of Defense guidelines initiative. Diabetes Care. 2004;27 Suppl 2:B82-B89.
19. Diabetes QUERI Center. 2008 QUERI Strategic Plan. Kerr EA, editor. 2008. Department of Veterans Affairs Quality Enhancement Research Initiative (QUERI). 10-20-2011.
20. Bodenheimer T, Lorig K, Holman H, Grumbach K. Patient Self-management of Chronic Disease in Primary Care. JAMA: The Journal of the American Medical Association. 2002;288:2469-2475.
21. Ostbye T, Yarnall KSH, Krause KM, Pollak KI, Gradison M, Michener JL. Is There Time for Management of Patients With Chronic Diseases in Primary Care? The Annals of Family Medicine. 2005;3:209-214.
22. VA Patient Centered Primary Care Implementation Work Group. Patient Centered Medical Home Model Concept Paper. 2010. 10-13-0011.
23. Piette J, Holtz B, Beard A, et al. Improving chronic illness care for veterans within the framework of the Patient-Centered Medical Home: experiences from the Ann Arbor Patient-Aligned Care Team Laboratory. Translational Behavioral Medicine. 2011;1-9.
24. Locke EA, Latham GP. A Theory of Goal Setting and Task Performance. Englewood, NJ: Prentice Hall, 1990.
25. Schillinger D, Handley M, Wang F, Hammer H. Effects of self-management support on structure, process, and outcomes among vulnerable patients with diabetes: a three-arm practical clinical trial. Diabetes Care. 2009;32:559-566.
26. DeWalt D, Broucksou K, Hawk V, et al. Comparison of a one-time educational intervention to a teach-to-goal educational intervention for self-management of heart failure: design of a randomized controlled trial. BMC Health Services Research. 2009;9:99.
27. Schillinger D, Hammer H, Wang F, et al. Seeing in 3-D: Examining the Reach of Diabetes Self-Management Support Strategies in a Public Health Care System. Health Educ Behav. 2007;35:664-682.
28. Weinger K, Beverly EA, Lee Y, Sitnokov L, Ganda OP, Caballero AE. The Effect of a Structured Behavioral Intervention on Poorly Controlled Diabetes: A Randomized Controlled Trial [published online ahead of print October 10 2011]. Arch Intern Med. 2011. http://www.ncbi.nlm.nih.gov/pubmed/21986346. Accessed November 4, 2011.

29. Frosch DL, Uy V, Ochoa S, Mangione CM. Evaluation of a Behavior Support Intervention for Patients With Poorly Controlled Diabetes [published online ahead of print October 10 2011]. Arch Intern Med. 2011; http://archinte.ama-assn.org/cgi/content/full/archinternmed.2011.497v1. Accessed November 5, 2011.
30. Naik AD, Schulman-Green D, McCorkle R, Bradley EH, Bogardus ST, Jr. Will older persons and their clinicians use a shared decision-making instrument? J Gen Intern Med. 2005;20:640-643.
31. Schulman-Green DJ, Naik AD, Bradley EH, McCorkle R, Bogardus ST. Goal setting as a shared decision making strategy among clinicians and their older patients. Patient Educ Couns. 2006;63:145-151.
32. Naik AD, Issac TT, Street RL, Jr., Kunik ME. Understanding the quality chasm for hypertension control in diabetes: a structured review of "co-maneuvers" used in clinical trials. J Am Board Fam Med. 2007;20:469-478.
33. Naik AD, Kallen MA, Walder A, Street RL, Jr. Improving hypertension control in diabetes mellitus: the effects of collaborative and proactive health communication. Circulation. 2008;117:1361-1368.
34. Parchman ML, Zeber JE, Palmer RF. Participatory Decision Making, Patient Activation, Medication Adherence, and Intermediate Clinical Outcomes in Type 2 Diabetes: A STARNet Study. The Annals of Family Medicine. 2010;8:410-417.
35. Lorig K. Action Planning: A Call To Action. The Journal of the American Board of Family Medicine. 2006;19:324-325.
36. Remmers C, Hibbard J, Mosen DM, Wagenfield M, Hoye RE, Jones C. Is Patient Activation Associated With Future Health Outcomes and Healthcare Utilization Among Patients With Diabetes? The Journal of Ambulatory Care Management. 2009;32.
37. Schillinger D, Grumbach K, Piette J, et al. Association of Health Literacy With Diabetes Outcomes. JAMA: The Journal of the American Medical Association. 2002;288:475-482.
38. Fransen MP, von Wagner C, Essink-Bot ML. Diabetes self-management in patients with low health literacy: Ordering findings from literature in a health literacy framework. Patient Educ Couns. 2011 Dec 23.
39. Cavanaugh K, Wallston KA, Gerbretsadik T, et al. Addressing literacy and numeracy to improve diabetes care: two randomized controlled trials. Diabetes Care. 2009 Dec;32(12):2149-55.
40. Naik AD, Street RL, Jr., Castillo D, Abraham NS. Health literacy and decision making styles for complex antithrombotic therapy among older multimorbid adults. Patient Educ Couns. 2011;85:499-504.
41. Lindau ST, Basu A, Leitsch SA. Health Literacy as a Predictor of Follow-Up After an Abnormal Pap Smear: A Prospective Study. Journal of General Internal Medicine. 2006;21:829-834.
42. Bass PF, III, Wilson JF, Griffith CH, Barnett DR. Residents' ability to identify patients with poor literacy skills. Acad Med. 2002;77:1039-1041.
43. Seligman HK, Wang FF, Palacios JL, et al. Physician Notification of Their Diabetes Patients' Limited Health Literacy. J Gen Intern Med. 2005;20:1001-1007.
44. Woodard LD, Petersen LA. Improving the performance of performance measurement. J Gen Intern Med. 2010;25:100-101.
45. Curran GM, Bauer M, Mittman B, Pyne JM, Stetler C. Effectiveness-implementation hybrid designs: combining elements of clinical effectiveness and implementation research to enhance public health Impact. Med Care 2012, 50: 217-226.
46. Petersen LA, Woodard LD, Henderson LM, Urech TH, Pietz K. Will hypertension performance measures used for pay-for-performance programs penalize those who care for medically complex patients? Circulation. 2009;119:2978-2985.
47. Woodard LD, Landrum CR, Urech TH, Profit J, Petersen LA. Treating chronically ill diabetic patients with limited life expectancy: implications for performance measurement. J Am Geriatr Soc. 2011;60(2):193-201.
48. Teal CR, Haidet P, Balasubramanyam AS, Rodriguez E, Naik AN. Measuring the Quality of Patients' Goals and Action Plans: Development and Validation of a Novel Tool. BMC Medical Informatics and Decision Making. 2012; Under review.

49.Hibbard JH, Stockard J, Mahoney ER, and Tusler M. Development of the Patient Activation Measure (PAM): Conceptualizing and Measuring Activation in Patient and Consumers. Health Services Research. 2004; 39(4): 1005-1026.
50.Chew LD, Bradley KA, Boyko EJ. Brief questions to identify patients with inadequate health literacy. Fam Med. 2004;36:588-594.
51.Kitson AL, Rycroft-Malone J, Harvey G, McCormack B, Seers K, Titchen A. Evaluating the successful implementation of evidence into practice using the PARiHS framework: theoretical and practical challenges. Implement Sci. 2008;3:1.
52.Helfrich CD, Damschroder LJ, Hagedorn HJ, et al. A critical synthesis of literature on the promoting action on research implementation in health services (PARIHS) framework. Implement Sci. 2010;5:82.
53.Helfrich CD, Li YF, Sharp ND, Sales AE. Organizational readiness to change assessment (ORCA): development of an instrument based on the Promoting Action on Research in Health Services (PARIHS) framework. Implement Sci. 2009;4:38.
54.Stetler CB, Damschroder LJ, Helfrich CD, Hagedorn HJ. A Guide for applying a revised version of the PARIHS framework for implementation. Implement Sci. 2011;6:99.
55.Miles MB, Huberman AM. Qualitative Data Analysis: An expanded source book. 2nd ed. Thousand Oaks, CA: 1994.
56.Silverman D. Doing Qualitative Research: A Practical Handbook. Thousand Oaks, CA: 2000.
57.Strauss AL, Corbin JM. Grounded Theory in Practice. 2nd ed. Thousand Oaks, CA: 1998.
58.Denzin NK, Lincoln YS. The Sage handbook of Qualitative Research. 3rd ed. Thousand Oaks: Sage Publications, 2005.
59.Spencer L, Ritchie J, O'Conner W. Analysis: practices, principles and processes. In: Ritchie J, Lewis J, eds. Qualitative Research Practice: A guide for social science students and researchers. Thousand Oaks: Sage Publications; 2003;199-218.
60.Linstone HA, Turoff M. The Delphi Method: Techniques and Applications. [e-book]. Linstone HA, Turoff M; 2002. Available from: Findlay.edu. Accessed June 4, 2011.
61.Heisler M, Vijan S, Anderson RM, Ubel PA, Bernstein SJ, Hofer TP. When do patients and their physicians agree on diabetes treatment goals and strategies, and what difference does it make? J Gen Intern Med. 2003;18:893-902.
62.Glasgow RE, Wagner EH, Schaefer J, Mahoney LD, Reid RJ, Greene SM. Development and Validation of the Patient Assessment of Chronic Illness Care (PACIC). Medical Care. 2005; 43(5): 436-444.
63.Johnson JA, Coons SJ, Ergo A, Szava-Kovats G. Valuation of EuroQOL (EQ-5D) health states in an adult US sample. Pharmacoeconomics. 1998;13(4):421-33.
64.Herdman M, Gudex C, Lloyd A, et al. Development and preliminary testing of the new five-level version of EQ-5D (EQ-5D-5L). Qual Life Res. 2011; 20(10):1727-36.
65.Fisher L, Polonsky W, Parkin CG, Jelsovsky Z, Amstutz L, Wagner RS. The impact of blood glucose monitoring on depression and distress in insulin-naive patients with type 2 diabetes. Current Medical Research and Opinion. 2011; 3:39-46.
66.Shnurr PP, Freidman MJ, Engel CC, et al.Issues in the design of multisite clinical trials of psychotherapy: VA Cooperative Study No. 494 as an example. Contemporary Clinical Trials. 2005; 26: 626-636.
67.Raudenbush SW et al. (2011). Optimal Design Softare for Multilevel and Longitudinal Research (Version 3.01) [Software].
68.Heisler M, Vijan S, Makki F, Piette JD. Diabetes control with reciprocal peer support versus nurse care management: a randomized trial. Ann Intern Med. 2010;153:507-515.
69.West DS, Dilillo V, Bursac Z, Gore SA, Greene PG. Motivational interviewing improves weight loss in women with type 2 diabetes. Diabetes Care. 2007; 30:1081-1087.
70.Fischer HH, Eisert SL, Everhart RM, et al. Nurse-run, telephone-based outreach to improve lipids in people with diabetes. Am J Manag Care. 2012;18:77-84.

71.Hawkins SY. Improving glycemic control in older adults using a videophone motivational diabetes self-management intervention. Research and Theory for Nursing Practice: An International Journal. 2010;24:217-232.
72.Prochaska JO, DiClemente CC. Stages and processes of self-change of smoking: Toward an integrative model of change. Journal of Consulting and Clinical Psychology. 1983; 51: 390–395.
73.Prochaska, JO, DiClemente CC. The transtheoretical approach: Crossing the traditional boundaries of change. Homewood, IL: Irwin; 1984.
74.Chen EH, Thom DH, Hessler DM, et al. Using a teamlet model to improve chronic care in an academic primary care practice. J Gen Intern Med. 2010; 25:610-614.
75.Sharons A, Abramowitz MD, Flattery D, Franses K, Berry L. Linking a motivational interviewing curriculum to the chronic care model. J Gen Intern Med. 2010; 25: 620-626.
76.Bodenheimer T, Davis C, Holman H. Helping patients adopt healthier behaviors. Clinical Diabetes. 2007; 25:66-70.
77.DeWalt DA, Callahan LF, Hawk VH, et al. Health Literacy Universal Precautions Toolkit. (Prepared by North Carolina Network Consortium, The Cecil G. Sheps Center for Health Services Research, The University of North Carolina at Chapel Hill, under Contract No. HHSA290200710014.) AHRQ Publication No. 10-0046-EF) Rockville, MD. Agency for Healthcare Research and Quality. April 2010.
78.National Patient Safety Association (NPSA) Web site. Available at http://www.npsf.org/for-healthcare-professionals/programs/ask-me-3/. Accessed May 30, 2012
79.Cully JA, Mignogna J, Stanley MA, et al. Development and pilot testing of a standardized training program for a patient-mentoring intervention to increase adherence to outpatient HIV care. AIDS Patient Care STDS. 2012;26(3):165-72.
80.Cully JA, Teten AL, Benge JF, Sorocco KH, Kauth MR. Multidisciplinary cognitive-behavioral therapy training for the veterans affairs primary care setting. Prim Care Companion J Clin Psychiatry. 2010;12(3).
81. Larsen DL, Attkisson CC, Hargreaves WA, Nguyen TD. Assessment of client/patient satisfaction: Development of a general scale. Evaluation and Program Planning. 1979; 2, 197-207.
82. Attkisson CC, Zwick RJ.The Client Satisfaction Questionnaire: Psychometric properties and correlations with service utilization and psychotherapy outcome. Evaluation and Program Planning. 1982; 5(3), 233-237.
83..Callahan CM, Unverzagt FW, Hui SL, Perkins AJ, Hendrie HC. Six-item screener to identify cognitive impairment among potential subjects for clinical research. Med Care. 2002;40:771-781.
84.Sheehan DV, Lecrubier Y, Sheehan KH, et al. The Mini-International Neuropsychiatric Interview (M.I.N.I.): the development and validation of a structured diagnostic psychiatric interview for DSM-IV and ICD-10. J Clin Psychiatry. 1998;59 Suppl 20:22-33.
85.Rohde P, Lewinsohn PM, Seeley JR. Comparability of telephone and face-to-face interviews in assessing axis I and II disorders. Am J Psychiatry. 1997;154:1593-1598.
86.Flegel K. Controlling the complications of diabetes: It's about the sugar. Canadian Medical Association Journal. 2009;181:357.
87.Polonsky WH, Fisher L, Earles J, et al. Assessing psychosocial distress in diabetes: development of the diabetes distress scale. Diabetes Care. 2005;28:626-631.
88.Fisher L, Skaff MM, Mullan JT, et al. Clinical depression versus distress among patients with type 2 diabetes: not just a question of semantics. Diabetes Care. 2007;30:542-548.
89.Lorig KR, Ritter P, Stewart AL, et al. Chronic disease self-management program: 2-year health status and health care utilization outcomes. Med Care. 2001;39:1217-1223.
90.Chew LD, Griffin JM, Partin MR, et al. Validation of screening questions for limited health literacy in a large VA outpatient population. J Gen Intern Med. 2008;23:561-566.
91.Rothman RL, Malone R, Bryant B. et al. The spoken knowledge in low literacy in diabetes scale: a diabetes knowledge scale for vunerable patients.The Diabetes EDUCATOR. 2005;31(2):215-224.
92.Jeppesen KM, Coyle JD, Miser WF. Screening Questions to Predict Limited Health Literacy: A Cross-Sectional Study of Patients With Diabetes Mellitus. Ann Fam Med. 2009; 7(1): 24-31.

93. Morisky DE, Ang A., Krousel-Wood M, Ward H. Predictive Validity of Medication Adherence Measure for Hypertension Control. Journal of Clinical Hypertension.2008; 10: 348-354.
94. Kroenke K, Strine TW, Spritzer RL, Williams JB, Berry JT, Mokdad AH. The PHQ-8 as a measure of current depression in the general population. J Affect Disord. 2009; 114(1-3):163-73.
95. Razykov I, Ziegelstein RC, Whooley MA, Thombs BD. The PHQ-9 versus the PHQ-8--is item 9 useful for assessing suicide risk in coronary artery disease patients? Data from the Heart and Soul Study. J Psychosom Res. 2012; 73(3):163-168.
96. Lorig K, Stewart A, Ritter P, González V, Laurent D, & Lynch J, Outcome Measures for Health Education and other Health Care Interventions. Thousand Oaks CA: Sage Publications, 1996;25:37-38.
97.Toobert DJ, Hampson SE, Glasgow RE. The summary of diabetes self-care activities measure: results from 7 studies and a revised scale. Diabetes Care. 2000;23:943-950.
98.Bendel RB, Afifi AA. Comparisons of stopping rules in forward regression. J Am Stat Assoc. 1977;72:46-53.
99.Rubin DB. Inference and missing data. Biometrika. 1976;63:581-590.
100.Shrout PE, Bolger N. Mediation in experimental and nonexperimental studies: New procedures and recommendations. Psychological Methods. 2002; 7(4): 422-445.
101.Muthén LK, Muthén BO. Mplus User's Guide. Sixth Edition. 1998-2010. Los Angeles, CA: Muthén & Muthén.

Version	Date	File name	Notes
Initial Protocol	11/7/2014		
Initial Approval Letter	3/3/2015	Final PI Approval 14-24	
Initial Patient ICF	3/3/2015	Oct162014_ICF-VA-1086 Update Patients_2015Feb20	
Initial Provider ICF	3/3/2015	Nov62014_ICF-VA-1086 Update Providers 2015Feb2	
Amendment 1-Form 116	4/21/2015	116 Request to Amend or Modify 080213_CIRB 14-24_LW	revised the EUC protocol; revised signer on the EUC recruitment letter to be LSI
Amendment 2- Form 116	4/30/2015	116 2015April29 Amend CIRB 14-24_ Amendment 2	Blinded staff for data collection; new FHL/PAM matrix; added satisfaction survey for patient perceptions of satisfaction with providers; added ability to recruit more provider participants after Phase 1; amended procedure tocreate the recruitment cohort; amended the survey abotuproior exposure to diabetes education; amended EUC protocol
Amendments 1 and 2 Approval Letter	6/3/2015	PISC Amendments 1 and 2 Letter 14-24	approved together given the short time between submissions
Amendment 1 and 2 Protocol	4/16/2015	2015April20_Amendment1_EPICprotocol_clean	
Amendment 1 and 2 Patient ICF	6/3/2015	2015April21_ICF-VA-1086 Update Patients_	
Amendment 3- Form 116	2/12/2016	FinalSignedForm116_2016Feb17_116 Request to Amend or Modify_CIRB 14-24	added Houston as an enrollment site; added the PAM as a measure to be collected at both follow up timepoints; amended the follow up data collection timepoints to allowfor the implementation time between screening/baseline and the start of the intervention; amended the patient payment procedures
Amendment 3 Approval Letter	3/1/2016	14-24 PI Amendment 3 Approval Letter	
Amendment 3 Protocol	2/17/2016	Final_2016Feb17_Amendment3_EPICprotocol_Clean	
Amendment 3 Patient ICF	2/29/2016	Final_2016Feb17_ICF-VA-1086 Update Patients_Amend3_	
Amendment 4- Form 116	3/8/2016	2016Mar8_Form116	revised the Providers IFC to reflect Houston as an enrollment site
Amendment 4 Approval Letter	3/15/2016	14-24 PI Amendment 4 Letter	
Amendment 4 Protocol	N/A	only an update to the ICF form	
Amendment 4 Providers ICF	3/15/2016	2016Mar8_ICF-VA-1086 Update Providers_	
Amendment 4 Providers ICF (updated page numbers in headers)	3/15/2016	2016Mar17_ICF-VA-1086_Providers(update)_032216	
Amendment 5- Form 116	5/5/2016	2016May6_Form116	revised the Hines and Jesse Brown LSI ICF for providers to reflect the correct name of the LSI and not the PISC
Amendment 5 Approval Letter	5/22/2016	14-24 PI Amendment 5 Approval Letter dated 5.20.16	
Amendment 5 Protocol	N/A	only an update to the local site ICF forms	
Amendment 5 ICF	N/A	no update to PISC model IFC	
Amendment 6- Form 116	11/7/2016	Form116_Signed_Amendment6	increased enrollment target to include screen failures who were enrolled, but did not participate; increased target for enrolled providers to meet interest of local staff
Amendment 6 Approval Letter	11/22/2016	Form116_Signed_Amendment6	
Amendment 6 Protocol	2/17/2016	02. Study Protocol v.3_021716	

	Amendment 6 ICF	N/A	did not require an update to the ICF	
2237	2015 PISC Renewal- Form 115b	11/2/2015	WoodardL_115bPISCApplicationForContinuingReview_01Sept2015	
	2015 PISC Renewal Approval Letter	12/28/2015	2015Dec28_CR PISC Approval Expedited	renewed until 1/8/2017
	2016 PISC Renewal- Form 115b	11/7/2016	Woodard.Form115b.2016Nov7	
	Houston LSI Renewal 2016- Form 115a	11/7/2016	Woodard.Form115a.2016Nov7	
	2016 PISC Renewal Approval Letter	12/27/2016	2016Dec27_Continuing Review PI Approval	renewed until 1/8/2018
	2017 PISC Renewal- Form 115a	11/2/2017	Woodard.115a.2017Nov2	
	Houston LSI Renewal 2017- Form 115b	11/3/2017	Woodard.115b.2017Nov3	
	PISC Renewal Approval Letter 2017	12/12/2017	2017Dec13_Continuing Review PI Approval	renewed until 1/8/2019
	2018 PISC Renewal- Form 115a	11/7/2018	14-24_115a_2018Nov7 (003)_LW	
	Houston LSI Renewal 2018- Form 115b	11/7/2018	14-24_115b_Houston_Nov7 (003)_LW	
	PISC Renewal Approval Letter 2018	12/18/2018	CR PISC Approval Expedited	renewed until 1/8/2020
	Initial Houston LSI Application- Form 104	3/14/2016	104 Woodard_LSI_104_3.14	
	Initial Houston LSI Approval Letter	4/7/2016	Initial Review Houston LSI Approval Letter 14-24	
	Initial Houston LSI Patient ICF	4/7/2016	2016Feb17_ICF-VA-1086_Patients_Houston	
	Initial Houston LSI Provider ICF	4/7/2016	2016Mar17_ICF-VA-1086_Providers_Houston	
	2017 LSI Houston Renewal- Form 115b	11/3/2017	Woodard.115b.2017Nov3	
	2017 LSI Houston Approval Letter	12/12/2017	CR LSI Approval Houston	renewed until 1/8/2019
	2018 LSI Houston Renewal- Form 115b	11/7/2018	14-24_115b_Houston_Nov7 (003)_LW	
	2018 LSI Houston Approval Letter	12/18/2018	CR LSI Approval Houston	renewawed until 1/8/2020
