# Supplementary material for: Comparison of Collaborative Goal Setting With Enhanced Education for Managing Diabetes-Associated Distress and Hemoglobin A1c Levels: A Randomized Clinical Trial
Source: JAMA Netw Open. 2022 May 4;5(5):e229975. doi: 10.1001/jamanetworkopen.2022.9975 (PMC9069258; doi:10.1001/jamanetworkopen.2022.9975)
Supplement: Supplement 2. — eFigure 1. Framework for Empowering Patients in Chronic Care (EPICC) eFigure 2. Hemoglobin A1c Over Time for Enhanced Usual Care (EUC) by Exposure to an EPICC Clinician Between Postintervention and Maintenance eTable. Differences in Baseline Characteristics of Participants by Completer Status [file jamanetwopen-e229975-s002.pdf]

Supplementary Online Content

Woodard L, Amspoker AB, Hundt NE, et al. Comparison of collaborative goal setting with enhanced education for managing diabetes-associated distress and hemoglobin A_{1c} levels: a randomized clinical trial. *JAMA Netw Open*. 2022;5(5):e229975. doi:10.1001/jamanetworkopen.2022.9975

eFigure 1. Framework for Empowering Patients in Chronic Care (EPICC)

eFigure 2. Hemoglobin A_{1c} Over Time for Enhanced Usual Care (EUC) by Exposure to an EPICC Clinician Between Postintervention and Maintenance

eTable. Differences in Baseline Characteristics of Participants by Completer Status

This supplementary material has been provided by the authors to give readers additional information about their work.

© 2022 Woodard L et al. *JAMA Network Open*.

eFigure 1. Framework for Empowering Patients in Chronic Care (EPICC)

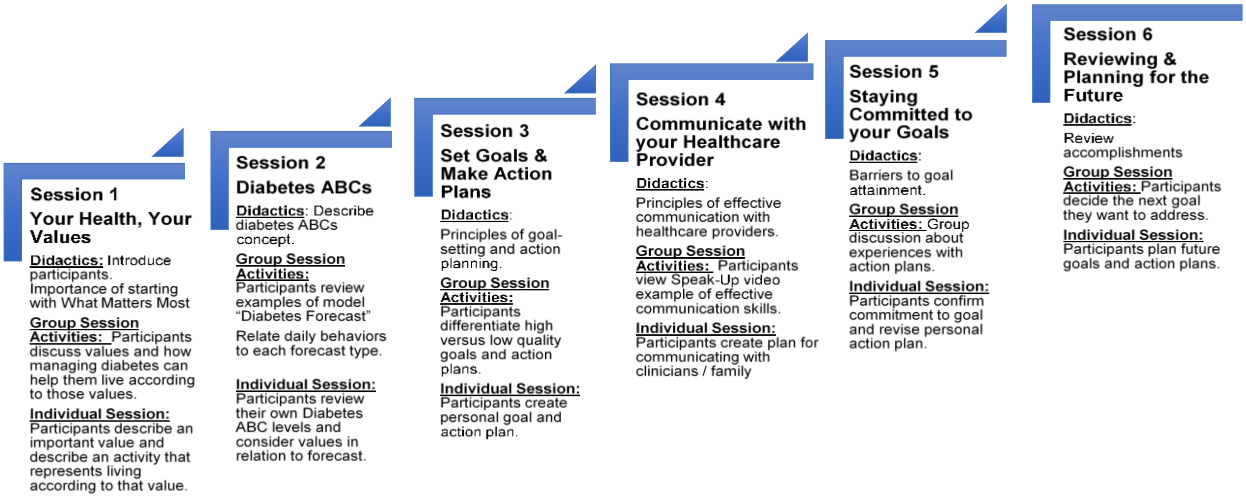

Group Sessions:

Each Session was an hour in duration, led by 1-2 clinic based health professionals. The first 20 minutes included an introduction and review of brief didactic materials. The middle portion of each session reviewed case examples and group discussion. The final 20 minutes provided group discussion that allow participants to begin to personalize the content their own lives.

Individual Sessions:

Health professionals used motivational interviewing techniques to help participants create a personal plan related to the session contents, review progress from prior visits, and help the participant with problem-solving tasks. Individual sessions had an average duration of 10 minutes per participant following each group session.

© 2022 Woodard L et al. *JAMA Network Open*.

eFigure 2. Hemoglobin A1c Over Time for Enhanced Usual Care (EUC) by Exposure to an EPICC Provider Between Postintervention and Maintenance

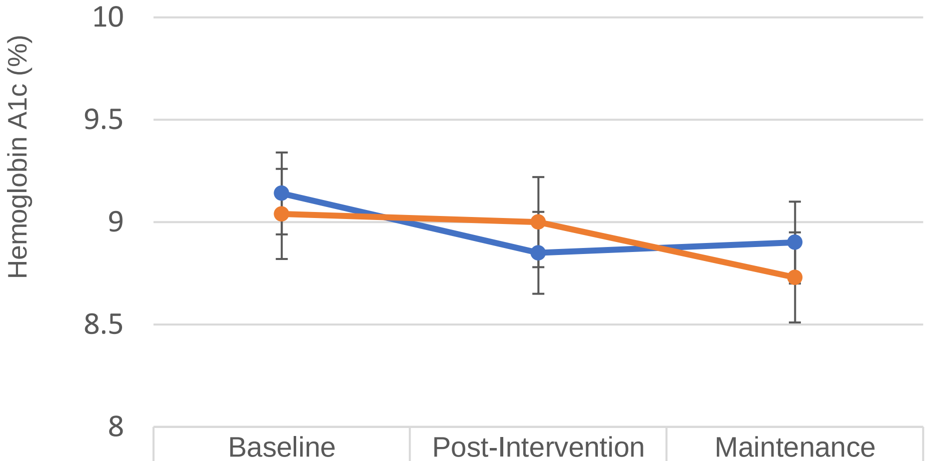

	Baseline	Post-Intervention	Maintenance
EUC- No visits with a Provider between Post-Intervention and Maintenance (n = 51)	9.14	8.85	8.9
EUC- At least 1 visit with a Provider between Post-Intervention and Maintenance (n = 53)	9.04	9	8.73

Assessment Period

© 2022 Woodard L et al. *JAMA Network Open*.

eTable. Differences in Baseline Characteristics of Participants by Completer Status			
	Non-Completer (n = 50)	Completer (n = 230)	p-value[a]
Site, N (%)			0.50
Community Clinic A	13 (26.0)	66 (28.7)	
Community Clinic B	6 (12.0)	48 (20.9)	
Facility Clinic C	14 (28.0)	50 (21.7)	
Facility Clinic D	8 (16.0)	37 (16.1)	
Facility Clinic E	9 (18.0)	29 (12.6)	
Gender, N (%)			0.15
Female	45 (90.0)	219 (95.2)	
Male	5 (10.0)	11 (4.8)	
Age in years, mean (SD)	67.3 (8.4)	67.1 (8.5)	0.93
Race/Ethnicity, N (%)			0.82[c]
Black	21 (42.0)	86 (37.4)	
Hispanic	7 (14.0)	26 (11.3)	
Non-Hispanic White	21 (42.0)	113 (49.1)	
Other[b]	1 (2.0)	5 (2.2)	
Education, N (%)			0.21
High school grad or less	16 (32.0)	54 (23.5)	
Some college or more	34 (68.0)	176 (76.5)	
Annual income, N (%), N = 258			0.63
< $20,000	12 (27.9)	68 (31.6)	
$20,000-$39,999	11 (25.6)	64 (29.8)	
≥ $40,000	20 (46.5)	83 (38.6)	
Employment, N (%), N = 270			0.51
Any employment	5 (10.6)	35 (15.7)	
Unemployed	38 (80.9)	176 (78.9)	
Retired or disabled	4 (8.5)	12 (5.4)	
Married/Living With Someone, N (%), n = 277	26 (52.0)	120 (52.9)	0.91
Lives Alone, N (%), N = 278	20 (40.0)	69 (30.3)	0.18
Perceived health, mean (SD)	3.5 (0.7)	3.46 (0.9)	0.77
Prior diabetes education, N (%)	29 (58.0)	133 (57.8)	0.98
Hemoglobin (Hb) A1c, mean (SD)	9.1 (1.6)	9.08 (1.4)	0.89
Diabetes Distress Score, mean (SD), n= 273	2.6 (1.1)	2.38 (1.0)	0.14
Diabetes Distress Score cut-points, n (%), N = 273			0.29
Little to No Distress (< 2.0)	16 (33.3)	88 (39.1)	
Moderate Distress (2.0 – 2.9)	15 (31.3)	82 (36.4)	
High Distress (≥ 3.0)	17 (35.4)	55 (24.4)	
Adherence, mean (SD), N = 274	3.7 (2.2)	3.5 (2.1)	0.53
Self-efficacy, mean (SD), N = 275	5.4 (2.2)	5.7 (2.4)	0.31

[a] For comparison between completers and non-completers, using independent samples t-tests (for interval variables) or chi-square tests (for ordinal variables)
[b] Multiracial (White, American Indian, and Other endorsed n = 4), Other – Not Specified (n = 2)
[c] Comparison of Non-Hispanic White versus others

© 2022 Woodard L et al. *JAMA Network Open*.
