# Supplementary material for: Comparison of Collaborative Goal Setting With Enhanced Education for Managing Diabetes-Associated Distress and Hemoglobin A1c Levels: A Randomized Clinical Trial
Source: JAMA Netw Open. 2022 May 4;5(5):e229975. doi: 10.1001/jamanetworkopen.2022.9975 (PMC9069258; doi:10.1001/jamanetworkopen.2022.9975)
Supplement: Supplement 3. — Data Sharing Statement [file jamanetwopen-e229975-s003.pdf]

Data Sharing Statement

Woodard. Comparison of Collaborative Goal Setting With Enhanced Education for Managing Diabetes-Associated Distress and Hemoglobin A_{1c} Levels. *JAMA Netw Open*. Published May 04, 2022. doi:10.1001/jamanetworkopen.2022.9975

Data
Data available: No

Additional Information
Explanation for why data not available: VA privacy rules limit availability of patient data
